# Causal effects of cardiovascular health on five epigenetic clocks

**DOI:** 10.1186/s13148-024-01752-5

**Published:** 2024-09-27

**Authors:** Hsien-Liang Sung, Wan-Yu Lin

**Affiliations:** 1 Institute of Health Data Analytics and Statistics, College of Public Health, National Taiwan University, Room 501, No. 17, Xu-Zhou Road, Taipei, 100 Taiwan; 2 Master of Public Health Degree Program, College of Public Health, National Taiwan University, Taipei, Taiwan

## Abstract

**Background:**

This work delves into the relationship between cardiovascular health (CVH) and aging. Previous studies have shown an association of ideal CVH with a slower aging rate, measured by epigenetic age acceleration (EAA). However, the causal relationship between CVH and EAA has remained unexplored.

**Methods and results:**

We performed genome-wide association studies (GWAS) on the (12-point) CVH score and its components using the Taiwan Biobank data, in which weighted genetic risk scores were treated as instrumental variables. Subsequently, we conducted a one-sample Mendelian Randomization (MR) analysis with the two-stage least-squares method on 2383 participants to examine the causal relationship between the (12-point) CVH score and EAA. As a result, we observed a significant causal effect of the CVH score on GrimAge acceleration (GrimEAA) (*β* [SE]: − 0.993 [0.363] year; *p* = 0.0063) and DNA methylation-based plasminogen activator inhibitor-1 (DNAmPAI-1) (β [SE]: − 0.294 [0.099] standard deviation (sd) of DNAmPAI-1; *p* = 0.0030). Digging individual CVH components in depth, the ideal total cholesterol score (0 [poor], 1 [intermediate], or 2 [ideal]) was causally associated with DNAmPAI-1 (*β* [SE]: − 0.452 [0.150] sd of DNAmPAI-1; false discovery rate [FDR] *q* = 0.0102). The ideal body mass index (BMI) score was causally associated with GrimEAA (β [SE]: − 2.382 [0.952] years; FDR *q* = 0.0498) and DunedinPACE (*β* [SE]: − 0.097 [0.030]; FDR *q* = 0.0044). We also performed a two-sample MR analysis using the summary statistics from European GWAS. We observed that the (12-point) CVH score exhibits a significant causal effect on Horvath’s intrinsic epigenetic age acceleration (*β* [SE]: − 0.389 [0.186] years; *p* = 0.036) and GrimEAA (β [SE]: − 0.526 [0.244] years; *p* = 0.031). Furthermore, we detected causal effects of BMI (*β* [SE]: 0.599 [0.081] years; *q* = 2.91E-12), never smoking (*β* [SE]: − 2.981 [0.524] years; *q* = 1.63E-7), walking (*β* [SE]: − 4.313 [1.236] years; *q* = 0.004), and dried fruit intake (*β* [SE]: − 1.523 [0.504] years; *q* = 0.013) on GrimEAA in the European population.

**Conclusions:**

Our research confirms the causal link between maintaining an ideal CVH and epigenetic age. It provides a tangible pathway for individuals to improve their health and potentially slow aging.

**Supplementary Information:**

The online version contains supplementary material available at 10.1186/s13148-024-01752-5.

## Introduction

The American Heart Association defined cardiovascular health (CVH) as a combination of three clinical factors (total cholesterol level, fasting glucose, and blood pressure) and four lifestyle factors (body mass index [BMI], smoking, physical activity, and dietary habits) [1]. Previous studies have shown that ideal CVH is inversely associated with the risk of several illnesses, including cardiovascular disease (CVD), chronic diseases, stroke, and type 2 diabetes mellitus (T2DM), as well as all-cause mortality [2–5]. Moreover, ideal CVH is associated with a longer lifespan and a better quality of life [6].

Epigenetic age is a promising molecular measure of biological age. It is constructed by DNA methylation (DNAm) levels at critical cytosine–phosphate–guanine sites (CpGs) [7–10], and it can dynamically reflect physiological conditions associated with human aging [11–13]. By regressing the “DNAm age” (or epigenetic age) on chronological age, we may obtain residuals serving as epigenetic age acceleration (EAA). With the advancement of epigenetics, EAA gradually becomes a popular measure of the human aging rate [14–18].

Epigenetic clocks can be roughly divided into three generations. The HannumAge [19] and HorvathAge [8] were called the first-generation epigenetic clocks, estimated by 71 and 353 CpGs, respectively. Both highly predict chronological age [9, 20] but are less related to CVH [21]. On the other hand, PhenoAge [22] and GrimAge [23, 24] were regarded as the second-generation epigenetic clocks. PhenoAge [22] was estimated by 513 CpGs predictive of a "phenotypic age." GrimAge [23, 24] was calculated by 1,030 CpGs associated with several plasma proteins and smoking pack-years. The first-generation epigenetic clocks focus on estimating chronological age [9, 25], while the second-generation counterparts take a step further to provide a more comprehensive picture of individuals’ physiological well-being [9, 25, 26].

Plasminogen activator inhibitor-1 (PAI-1) is a protein regulating the fibrinolytic system, which is involved in the breakdown of blood clots. Elevated levels of PAI-1 are associated with an increased risk of CVD [27, 28]. PAI-1 is also involved in cellular senescence and aging [29]. Being one DNAm-based surrogate plasma protein of GrimAge, DNAm-based PAI-1 (DNAmPAI-1, predicted by 211 out of the 1,030 GrimAge CpGs) was shown to be more associated with lipid metabolism (such as triglyceride and high-density lipoprotein cholesterol levels) than GrimAge [23, 24].

Moreover, a novel pace of aging, DunedinPACE, was developed by Belsky et al. recently [17]. It was built based on the longitudinal data from the Dunedin Study 1972–1973 birth cohort [30]. While the above-mentioned four epigenetic clocks predict human biological age, DunedinPACE estimates the pace of aging based on DNAm levels. DunedinPACE was regarded as the third-generation epigenetic clock, and it was shown to be associated with more Taiwanese health outcomes than the four above-mentioned measures of EAA [31].

Recent research has established an association link between ideal CVH and “epigenetic age deceleration” (EAD) in individuals of European descent [32, 33]. It has also been replicated in Asian (specifically, Taiwan) populations [21]. However, the causal relationship between CVH and EAD (or, inversely, EAA) has not been explored. Therefore, in this work, we performed a Mendelian Randomization (MR) analysis to confirm the causal relationship between CVH and five epigenetic clocks. Because DNAmPAI-1 (a DNAm-based surrogate plasma protein of GrimAge) is critical to lipid metabolism [23, 24], we included it in addition to the five measures of EAA.

## Results

### Genome‐wide association studies

Table 1 presents the basic characteristics of the 116,525 Taiwan Biobank (TWB) participants without DNAm data and 2,383 participants with DNAm data. The characteristics of the 2,383 individuals were similar to those of the 116,525 participants. Because only 17% (= 19,246/116,525) and 57% (= 1,361/2,383) of participants provided their dietary information, we calculated the CVH scores based on six metrics (without the diet-type score), i.e., the “12-point CVH score” [34].

**Table 1:** Baseline characteristics of 116,525 TWB participants (without DNAm data) and 2383 TWB participants (with DNAm data)

	TWB without DNAm (N = 116,525)	TWB with DNAm (N = 2,383)
Age (sd)	49.40 (11.16)	49.79 (11.06)
Male (%)	43,563 (37.39%)	1194 (50.10%)
Drinking (%)	7268 (6.24%)	168 (7.05%)
Educational attainment (sd)	5.56 (0.95)	5.58 (0.92)
Total cholesterol score (sd)	1.46 (0.68)	1.48 (0.67)
Fasting glucose score (sd)	1.74 (0.53)	1.75 (0.52)
Blood pressure score (sd)	1.35 (0.74)	1.39 (0.72)
BMI score (sd)	1.31 (0.80)	1.29 (0.79)
Smoking status score (sd)	1.80 (0.59)	1.75 (0.65)
Physical activity score (sd)	0.82 (0.97)	0.91 (0.98)
CVH score (sd)	8.49 (2.03)	8.57 (2.05)

Educational attainment is an integer ranging from 1 to 7: 1 represented no formal education and illiterate; 2 represented self-study and literate; 3 represented primary school; 4 represented junior high school; 5 represented senior high school; 6 represented undergraduate; 7 represented graduate or above; CVH score: the cardiovascular health score calculated based on six metrics (without the diet-type score), ranging from 0 to 12

With the set of 116,525 individuals, we performed genome-wide association studies (GWAS) to identify 15, 74, 37, 31, 34, 1, and 0 single-nucleotide polymorphisms (SNPs) associated with the 12-point CVH score (Table 2), total cholesterol (TC) score (Supplementary Table S1), fasting glucose (FG) score (Table S2), blood pressure (BP) score (Table S3), BMI score (Table S4), smoking (SMK) score (Table S5), and physical activity (PA) score. The Manhattan and QQ plots for these seven GWAS are presented in Supplementary Figures S1, S2. The QQ plots show that the large observed* p*-values match the expected* p*-values. Association signals appear at the right tail of the QQ plots where the observed p-values differ from expected.

**Table 2:** 15 nearly independent SNPs (*r*^*2*^ < 0.01) associated with the 12-point CVH score (p < 5E-8)

CHR	SNP	A1	A2	MAF	BP	BETA	SE	*p*-value
1	rs629301	G	T	0.07	109275684	0.0941	0.0156	1.59E-09
2	rs13306194	A	G	0.14	21029662	0.0672	0.0115	5.61E-09
6	rs4709395	G	A	0.24	160057757	0.0522	0.0094	2.85E-08
6	rs73596816	A	G	0.05	160596331	-0.1027	0.0180	1.12E-08
7	rs2908286	T	C	0.20	44195138	-0.0561	0.0101	2.71E-08
9	rs2519093	T	C	0.18	133266456	-0.0620	0.0104	2.47E-09
11	rs662799	G	A	0.27	116792991	-0.0735	0.0090	2.57E-16
11	rs72643557	T	C	0.41	61811955	-0.0536	0.0082	7.98E-11
12	rs10550903	CAA	C	0.32	89696509	0.0503	0.0087	8.39E-09
16	rs72805612	A	G	0.13	53800696	-0.0972	0.0117	1.25E-16
18	rs11082764	G	A	0.41	49593209	-0.0450	0.0082	3.62E-08
19	rs12972970	A	G	0.08	44884339	-0.0927	0.0147	2.58E-10
19	rs141622900	A	G	0.07	44923535	0.1606	0.0153	1.24E-25
19	rs3745683	A	G	0.26	11237845	0.0641	0.0092	2.67E-12
19	rs7246757	G	A	0.06	45687273	-0.0960	0.0175	3.95e-08

CHR: chromosome; SNP: single-nucleotide polymorphism; MAF: minor allele frequency; BP: base pair

We used the 15 CVH-associated SNPs to form a weighted genetic risk score (wGRS) for the CVH score (denoted as “CVH-wGRS”) by weighting the genotypes (of the 2,383 individuals) according to the corresponding effect sizes (estimated from the 116,525 individuals). Then, the wGRSs for CVH components were computed similarly, generating TC-wGRS, FG-wGRS, BP-wGRS, and BMI-wGRS. SMK-associated SNPs (1 SNP) and PA-associated SNPs (0 SNP) were insufficient to construct wGRSs, so we did not include these two components in the following one-sample MR analysis.

### TWB MR analysis: the CVH score and the CVH components on EAA

Before performing MR, we evaluated three MR assumptions to verify the validity of instrumental variables (IVs). For assumptions (2) and (3), we examined the relationship between CVH-wGRS (or TC-wGRS, FG-wGRS, BP-wGRS, BMI-wGRS), four confounding factors (chronological age, sex, drinking status, and educational attainment) [21] and EAA. Checking assumption (2), we found that sex was associated with CVH-wGRS (*p* = 0.0176); chronological age was associated with FG-wGRS (*p* = 0.0130); educational attainment was associated with BMI-wGRS (*p* = 0.0071); while drinking status was not related to any wGRS (Supplementary Table S6).

Checking assumption (3), we identified that DNAmPAI-1 was associated with CVH-wGRS (*p* = 0.0488), TC-wGRS (*p* = 0.0032), and FG-wGRS (*p* = 0.0414), while HannumEAA was associated with BMI-wGRS (*p* = 0.0164) (Supplementary Table S7). SNPs with the lowest p-value were removed until wGRS was independent of the confounding factors and EAA. The removal process can be found in Supplementary Tables S8–S11.

Take CVH-wGRS as an example. After checking assumptions (2) and (3), the CVH-wGRS was composed of 14 or 13 (for DNAmPAI-1) SNPs (Supplementary Table S8). We calculated the F statistic through the first-stage model to examine assumption (1) and evaluate the strength of IVs. As shown in Table 3, all F values were larger than 20, suggesting that CVH-wGRS was considered a strong IV for all measures of EAA [35].

**Table 3:** MR results between the CVH score and different measures of EAA in TWB

Outcome	F	IV	β (SE)	95% CI	p-value
HannumEAA	21.9	CVH-wGRS (14 SNPs)	− 0.442 (0.380)	(− 1.189, 0.301)	0.2426
IEAA	21.9	CVH-wGRS (14 SNPs)	− 0.206 (0.387)	(-0.965, 0.552)	0.5938
PhenoEAA	21.9	CVH-wGRS (14 SNPs)	− 0.163 (0.503)	(− 1.150, 0.824)	0.7461
GrimEAA	21.6	CVH-wGRS (14 SNPs)	− **0.993 (0.363)**	**(**− **1.705, **− **0.280)**	**0.0063**
DNAmPAI-1	24.3	CVH-wGRS (13 SNPs)	− **0.294 (0.099)**	**(**− **0.488, **− **0.100)**	**0.0030**
DunedinPACE	21.9	CVH-wGRS (14 SNPs)	− 0.021 (0.011)	(− 0.043, 0.001)	0.0637

IEAA: Horvath’s intrinsic epigenetic age acceleration; bold font indicates significant MR results (*p*-value < 0.05)

As shown in Table 3, the two-stage least-squares result indicates that the CVH score has a causal effect on GrimEAA (*β* [SE]: − 0.993 [0.363] years; *p* = 0.0063) and DNAmPAI-1 (*β* [SE]: − 0.294 [0.099] standard deviation [SD] of DNAmPAI-1; *p* = 0.0030). Improving one point on the (12-point) CVH score can decrease GrimEAA to 0.993 years and 0.294 SD of DNAmPAI-1. We then decomposed the CVH score into six metrics, each representing the ideal score (0, 1, or 2) of a CVH component (TC, FG, BP, BMI, SMK, or PA). For the CVH components, we used the false discovery rate (FDR) procedure to correct for multiple metrics of CVH. As shown in Table 4, the TC score has a causal effect on DNAmPAI-1 (*β* [SE]: − 0.452 [0.150] SD of DNAmPAI-1; *q* = 0.0102). BMI score has a causal impact on GrimEAA (*β* [SE]: − 2.382 [0.952] years; *q* = 0.0498) and DunedinPACE (β [SE]: − 0.097 [0.030]; *q* = 0.0044). In summary, our results suggest that maintaining an ideal CVH may decelerate the aging rate measured by GrimEAA and reduce DNAmPAI-1.

**Table 4:** MR results between CVH metrics and different EAA in TWB

	IV	*F*	*β* (SE)	95%CI	*p*-value	*q*-value
HannumEAA						
TC score	TC-wGRS (74 SNPs)	122.6	0.122 (0.510)	(− 0.879, 1.122)	0.8118	0.8655
FG score	FG-wGRS (33 SNPs)	43.7	0.180 (1.065)	(− 1.908, 2.269)	0.8655	0.8655
BP score	BP-wGRS (31 SNPs)	15.5	0.254 (1.273)	(− 2.252, 2.743)	0.8417	0.8655
BMI score	BMI-wGRS (28 SNPs)	18.6	− 2.541 (1.069)	(− 4.636, -0.446)	0.0175	0.0698
IEAA						
TC score	TC-wGRS (74 SNPs)	121.3	− 0.178 (0.518)	(− 1.193, 0.838)	0.7319	0.9759
FG score	FG-wGRS (33 SNPs)	44.1	0.605 (1.077)	(− 1.507, 2.717)	0.5743	0.9759
BP score	BP-wGRS (31 SNPs)	15.1	− 0.011 (1.316)	(− 2.594, 2.569)	0.9936	0.9936
BMI score	BMI-wGRS (32 SNPs)	21.2	− 1.014 (1.014)	(− 3.002, 0.973)	0.3171	0.9759
PhenoEAA						
TC score	TC-wGRS (74 SNPs)	121.3	0.392 (0.677)	(− 0.936, 1.719)	0.5631	0.8429
FG score	FG-wGRS (33 SNPs)	43.9	− 0.275 (1.389)	(− 3.000, 2.449)	0.8429	0.8429
BP score	BP-wGRS (31 SNPs)	15.3	− 0.400 (1.703)	(− 3.742, 2.938)	0.8141	0.8429
BMI score	BMI-wGRS (32 SNPs)	21.3	− 2.729 (1.322)	(− 5.323, -0.136)	0.0392	0.1566
GrimEAA						
TC score	TC-wGRS (74 SNPs)	121.1	− 0.313 (0.482)	(− 1.258, 0.632)	0.5163	0.6884
FG score	FG-wGRS (33 SNPs)	44.0	− 0.721 (1.014)	(− 2.708, 1.267)	0.4771	0.6884
BP score	BP-wGRS (31 SNPs)	15.5	0.331 (1.230)	(− 2.084, 2.739)	0.7876	0.7876
BMI score	BMI-wGRS (32 SNPs)	21.2	− **2.382 (0.952)**	**(**− **4.250, -0.514)**	**0.0125**	**0.0498**
DNAmPAI-1 (a DNAm-based surrogate plasma protein of GrimAge)						
TC score	TC-wGRS (68 SNPs)	104.6	− **0.452 (0.150)**	**(**− **0.745, -0.158)**	**0.0026**	**0.0102**
FG score	FG-wGRS (32 SNPs)	41.2	− 0.123 (0.306)	(-0.723, 0.477)	0.6878	0.6878
BP score	BP-wGRS (31 SNPs)	15.1	0.186 (0.358)	(− 0.520, 0.885)	0.6102	0.6878
BMI score	BMI-wGRS (32 SNPs)	21.2	− 0.470 (0.279)	(− 1.016, -0.076)	0.0920	0.1840
DunedinPACE						
TC score	TC-wGRS (74 SNPs)	121.7	0.005 (0.015)	(− 0.244, 0.034)	0.7368	0.7368
FG score	FG-wGRS (33 SNPs)	43.5	− 0.013 (0.031)	(− 0.074, 0.048)	0.6792	0.7368
BP score	BP-wGRS (31 SNPs)	15.3	0.031 (0.038)	(− 0.044, 0.106)	0.4229	0.7368
BMI score	BMI-wGRS (32 SNPs)	20.9	− **0.097 (0.030)**	**(**− **0.154, -0.039)**	**0.0011**	**0.0044**

TC score: ideal total cholesterol score; FG score: ideal fasting glucose score; BP score: ideal blood pressure score; BMI score: ideal body mass index score; Bold font indicates that the MR results are significant after the FDR correction (*q* < 0.05)

### EUR MR analysis: the CVH score on EAA

In addition to the above one-sample MR analysis, we followed Kong et al.’s study [36] to perform a two-sample MR analysis using European data. Summary statistics for HannumEAA, IEAA, PhenoEAA, GrimEAA, and DNAmPAI-1 were provided by a GWAS incorporating 34,710 Europeans [37]. DunedinPACE, developed in 2022 [17], was not investigated by this GWAS (published in 2021) [37]. Seventeen independent CVH-associated SNPs (*p* < 5E-8) were extracted from a GWAS of the VA Million Veteran Program, in which ~ 83% were European Americans and ~ 12% were African Americans [5]. Most subjects of the EAA GWAS [37] and the CVH GWAS [5] were of European ancestry.

Among the 17 independent CVH-associated SNPs [5], 11 were also investigated in the EAA GWAS [37]. These 11 SNPs were used as IVs for the two-sample MR analysis. Importantly, we found no evidence of pleiotropy (in SNPs) or heterogeneity (for the inverse variance weighted [IVW] estimates) in the causal inference of the CVH score on the five epigenetic markers (Supplementary Table S12). This validation led us to adopt the result of the IVW method.

As shown in Table 5, the IVW result indicates a significant negative causal association of the CVH score with IEAA ($\beta$ [SE]: − 0.389 [0.186] year; *p* = 0.0360) and GrimEAA ($\beta$ [SE]: − 0.526 [0.244] year; *p* = 0.0310). The CVH GWAS also provided summary statistics when analyzing individuals without CVD [5]. The effect sizes of the 17 CVH-associated SNPs were similar to those obtained from the entire cohort [5]. We performed a sensitivity analysis for the two-sample MR study. The observed causal effects remained consistent even if we used the summary statistics based on the individuals without CVD, i.e., IEAA ($\beta$ [SE]: − 0.404 [0.196] year; *p* = 0.0389) and GrimEAA ($\beta$ [SE]: − 0.551 [0.258] year; *p* = 0.0323).

**Table 5:** EUR MR analysis result: the CVH score on EAA

	No. of SNPs	*F*	IVW		Weighted Median		MR-Egger		MR-PRESSO		
			*β* (SE)	*p*-value	*β *(SE)	*p*-value	*β* (SE)	*p*-value	No. of outliers	*β* (SE)	*p*-value
*HannumEAA (years)*											
CVH score	11	33	− 0.095 (0.180)	5.99E-01	− 0.352 (0.243)	1.48E-01	− 0.040 (0.630)	9.51E-01	0	− 0.095 (0.176)	6.02E-01
CVH score (without CVD)	11	30	− 0.098 (0.190)	6.04E-01	− 0.325 (0.245)	2.00E-01	− 0.032 (0.589)	9.58E-01	0	− 0.098 (0.186)	6.08E-01
*IEAA (years)*											
CVH score	11	33	− **0.389 (0.186)**	**3.60E-02**	− 0.357 (0.239)	1.36E-01	0.265 (0.628)	6.83E-01	0	− **0.389 (0.116)**	**7.41E-03**
CVH score (without CVD)	11	30	− **0.404 (0.196)**	**3.89E-02**	− 0.377 (0.251)	1.33E-01	0.224 (0.587)	7.11E-01	0	− **0.404 (0.125)**	**8.85E-03**
*PhenoEAA (years)*											
CVH score	11	33	− 0.450 (0.248)	7.00E-02	− 0.376 (0.314)	2.31E-01	0.355 (0.869)	6.92E-01	0	− 0.450 (0.248)	1.00E-01
CVH score (without CVD)	11	30	− 0.461 (0.265)	8.18E-02	− 0.395 (0.341)	2.47E-01	0.371 (0.811)	6.58E-01	0	− 0.461 (0.265)	1.21E-01
*GrimEAA (years)*											
CVH score	11	33	− **0.526 (0.244)**	**3.10E-02**	− 0.466 (0.273)	8.70E-02	− 0.698 (0.868)	4.42E-01	0	− 0.526 (0.244)	5.64E-02
CVH score (without CVD)	11	30	− **0.551 (0.258)**	**3.23E-02**	− **0.592 (0.264)**	**2.47E-02**	− 0.610 (0.816)	4.73E-01	0	− 0.551 (0.258)	5.80E-02
*DNAm PAI-1 (pg/ml)*											
CVH score	11	33	− 0.586 (0.507)	2.48E-01	− 0.874 (0.640)	1.76E-01	− 2.110 (2.335)	3.90E-01	0	− 0.586 (0.507)	2.75E-01
CVH score (without CVD)	11	30	− 0.618 (0.541)	2.53E-01	− 0.727 (0.688)	2.91E-01	− 1.718 (2.218)	4.59E-01	0	− 0.618 (0.541)	2.80E-01

Bold font indicates significant MR results (*p* < 0.05)

### EUR MR analysis: the CVH factors on EAA

**Fig. 1:**
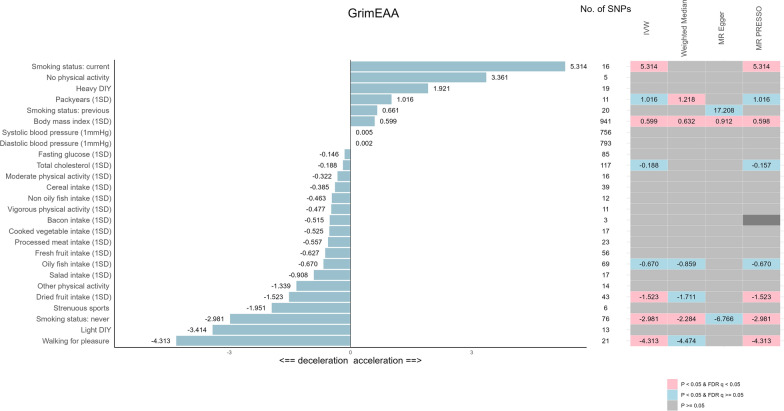
Causal effects of CVH factors on GrimEAA in EUR. For the plot in the left panel, the numbers shown around the blue bars are the causal effect sizes. The number of SNPs between the two-panel plots represents the number of IVs. For the plot in the right panel, red boxes indicate significant causal effects (*p*-value < 0.05 & FDR *q*-value < 0.05). Blue boxes indicate suggestive causal effects (*p*-value < 0.05 & FDR *q*-value ≥ 0.05). Gray boxes indicate insignificant causal effects (*p*-value ≥ 0.05). The dark gray box indicates insufficient IVs for the MR analysis. The box numbers represent the causal effect sizes achieving significant or suggestive associations

The three clinical factors (TC [38], FG [39], and BP [40]) and four lifestyle factors (BMI [41], SMK [42], PA [42], and dietary habits [42]) have been investigated by several GWAS (Supplementary Table S13). We performed the two-sample MR analysis to assess the causal effects of the CVH factors on the five epigenetic markers (Table 6 and Supplementary Tables S14–S17; Fig. 1 and Supplementary Figures S3–S6). Moreover, pleiotropy (in SNPs) and heterogeneity (for the IVW estimates) are examined in Supplementary Tables S18–S22.

**Table 6:** EUR MR analysis result: the CVH factors on GrimEAA

	No. of SNPs	F	IVW			Weighted median			MR-Egger			MR-PRESSO			
CVH factors			*β* (SE), year	*p* Value	*q* Value	*β* (SE), year	*p* Value	*q* Value	*β* (SE), year	*p* Value	*q* Value	No. of outliers	*β* (SE), years	*p* Value	*q* Value
*Clinical factors*															
Total cholesterol (1SD)	117	125	− 0.188 (0.084)	2.47E-02	9.18E-02	− 0.104 (0.128)	4.16E-01	8.77E-01	− 0.085 (0.137)	5.39E-01	8.72E-01	2	− 0.157 (0.078)	4.82E-02	1.51E-01
Fasting glucose (1SD)	85	109	− 0.146 (0.226)	5.18E-01	6.64E-01	− 0.003 (0.301)	9.93E-01	9.93E-01	0.368 (0.431)	3.95E-01	8.22E-01	1	− 0.106 (0.214)	6.21E-01	7.06E-01
DBP (1 mmHg)	793	65	0.002 (0.008)	8.23E-01	8.23E-01	− 0.002 (0.013)	8.62E-01	9.29E-01	− 0.009 (0.021)	6.71E-01	8.72E-01	2	0.006 (0.008)	4.99E-01	6.56E-01
SBP (1 mmHg)	756	64	0.005 (0.005)	3.51E-01	5.38E-01	− 0.002 (0.007)	7.39E-01	8.85E-01	− 0.013 (0.013)	3.26E-01	7.71E-01	4	0.005 (0.005)	2.80E-01	4.72E-01
*Lifestyle factors*															
BMI (1SD)	941	58	**0.599 (0.081)**	**1.12E-13**	**2.91E-12**	**0.632 (0.124)**	**3.58E-07**	**9.31E-05**	**0.912 (0.249)**	**2.60E-04**	**6.77E-03**	2	**0.598 (0.080)**	**1.48E-13**	**3.70E-12**
*Smoking status*															
Current	16	40	**5.314 (1.658)**	**1.35E-03**	**8.76E-03**	4.293 (2.224)	5.35E-02	1.99E-01	8.568 (7.513)	2.73E-01	7.71E-01	0	**5.314 (1.491)**	**2.83E-03**	**1.77E-02**
Previous	20	38	0.661 (1.357)	6.26E-01	6.79E-01	1.791 (1.478)	2.26E-01	5.87E-01	17.208 (7.011)	2.45E-02	2.12E-01	1	1.305 (1.180)	2.83E-01	4.72E-01
Never	76	41	− **2.981 (0.524)**	**1.25E-08**	**1.63E-07**	− **2.284 (0.746)**	**2.19E-03**	**2.84E-02**	− 6.766 (2.292)	4.22E-03	5.49E-02	0	− **2.981 (0.524)**	**2.30E-07**	**2.88E-06**
*Amount of smoking *															
Pack-years (1SD)	11	73	1.016 (0.413)	1.40E-02	6.06E-02	**1.218 (0.439)**	**5.49E-03**	**4.76E-02**	1.935 (0.950)	7.20E-02	4.68E-01	0	1.016 (0.413)	3.38E-02	1.27E-01
*Number of days/week of physical activity 10 + minutes*															
Moderate (1SD)	16	36	− 0.322 (0.277)	2.45E-01	5.38E-01	0.128 (0.364)	7.24E-01	8.85E-01	− 1.418 (1.795)	4.43E-01	8.22E-01	0	− 0.322 (0.277)	2.63E-01	4.72E-01
Vigorous (1SD)	11	40	− 0.477 (0.438)	2.76E-01	5.38E-01	− 0.272 (0.489)	5.79E-01	8.85E-01	− 3.032 (3.634)	4.26E-01	8.22E-01	0	− 0.477 (0.438)	3.02E-01	4.72E-01
*Types of physical activity in the last 4 weeks*															
Heavy DIY	19	35	1.921 (1.189)	1.06E-01	2.77E-01	2.136 (1.516)	1.59E-01	5.17E-01	0.321 (7.776)	9.68E-01	9.68E-01	0	1.921 (1.049)	8.38E-02	2.33E-01
Light DIY	13	40	− 3.414 (1.998)	8.75E-02	2.53E-01	0.270 (2.017)	8.93E-01	9.29E-01	10.396 (7.390)	1.87E-01	6.68E-01	1	− 2.322 (1.706)	2.01E-01	4.56E-01
Strenuous sports	6	39	− 1.951 (4.816)	6.85E-01	7.13E-01	− 2.548 (4.574)	5.78E-01	8.85E-01	3.982 (25.233)	8.82E-01	9.68E-01	0	− 1.951 (4.816)	7.02E-01	7.63E-01
Walking	21	34	− **4.313 (1.236)**	**4.85E-04**	**4.21E-03**	− 4.474 (1.825)	1.43E-02	7.41E-02	1.831 (12.868)	8.88E-01	9.68E-01	0	− **4.313 (1.218)**	**2.05E-03**	**1.71E-02**
Other physical activity	14	38	− 1.339 (1.385)	3.34E-01	5.38E-01	− 0.377 (1.851)	8.39E-01	9.29E-01	− 1.190 (10.917)	9.15E-01	9.68E-01	0	− 1.339 (1.385)	3.51E-01	4.88E-01
No physical activity	5	33	3.361 (5.439)	5.37E-01	6.64E-01	− 2.561 (6.426)	6.90E-01	8.85E-01	− 70.264 (59.182)	3.21E-01	7.71E-01	0	3.361 (5.439)	5.70E-01	6.79E-01
*Food intake*															
Dried fruit (1SD)	43	42	− **1.523 (0.504)**	**2.51E-03**	**1.31E-02**	− 1.711 (0.646)	8.04E-03	5.23E-02	− 3.149 (2.299)	1.78E-01	6.68E-01	0	− **1.523 (0.504)**	**4.27E-03**	**2.13E-02**
Fresh fruit (1SD)	56	45	− 0.627 (0.600)	2.96E-01	5.38E-02	− 0.804 (0.783)	3.05E-01	7.20E-01	1.126 (2.237)	6.17E-01	8.72E-01	1	− 0.847 (0.566)	1.55E-01	3.88E-01
Salad (1SD)	17	37	− 0.908 (1.222)	4.57E-01	6.60E-01	− 0.549 (1.312)	6.75E-01	8.85E-01	3.574 (6.274)	5.77E-01	8.72E-01	1	0.138 (1.067)	8.99E-01	8.99E-01
Cooked vegetable (1SD)	17	38	− 0.525 (1.061)	6.20E-01	6.79E-01	0.632 (1.098)	5.65E-01	8.85E-01	− 1.299 (11.902)	9.15E-01	9.68E-01	1	0.179 (0.838)	8.34E-01	8.69E-01
Oily fish (1SD)	69	44	− 0.670 (0.313)	3.21E-02	1.04E-01	− 0.859 (0.436)	4.86E-02	1.99E-01	− 0.621 (1.364)	6.50E-01	8.72E-01	0	− 0.670 (0.313)	3.57E-02	1.27E-01
Nonoily fish (1SD)	12	44	− 0.463 (0.819)	5.72E-01	6.76E-01	0.787 (1.123)	4.83E-01	8.85E-01	-5.450 (4.026)	2.06E-01	6.68E-01	0	-0.463 (0.786)	5.68E-01	6.79E-01
Cereal (1SD)	39	45	− 0.385 (0.411)	3.49E-01	5.38E-01	− 0.191 (0.596)	7.49E-01	8.85E-01	-2.646 (1.744)	1.38E-01	6.68E-01	0	− 0.385 (0.393)	3.33E-01	4.88E-01
Bacon (1SD)	3	32	− 0.515 (0.834)	5.37E-01	6.64E-01	− 0.782 (1.010)	4.39E-01	8.77E-01	− 0.107 (1.736)	9.61E-01	9.68E-01	–	–	–	–
Processed meat (1SD)	23	39	− 0.557 (0.518)	2.82E-01	5.38E-01	− 0.927 (0.708)	1.91E-01	5.51E-01	− 1.905 (2.698)	4.88E-01	8.46E-01	0	− 0.557 (0.518)	2.94E-01	4.72E-01

DBP: diastolic blood pressure; SBP: systolic blood pressure; BMI: body mass index; Moderate: number of days/week of moderate physical activity 10 + minutes; Vigorous: Number of days/week of vigorous physical activity 10 + minutes; Heavy DIY: e.g., weeding, lawn mowing, carpentry, digging; Light DIY: e.g., pruning, watering the lawn; Walking: walking for pleasure (not as a means of transport); Bold font indicates that the MR results are significant after FDR correction (*q* < 0.05)

The CVH factor analysis showed that BMI had a significant causal effect on GrimEAA (Table 6; Fig. 1; *β* per 1-SD increase in BMI [SE]: 0.599 [0.081] years; *q* = 2.91E-12), which was in line with the TWB analysis result (Table 4; *β* per 1-point increase in the BMI score [SE]: − 2.382 [0.952] years; *q* = 0.0498).

The BMI score is an integer ranging from 0 to 2; a higher score represents a more ideal BMI. Therefore, the causal effect of the BMI score on GrimEAA is negative (− 2.382 years, Table 4). In contrast, the BMI GWAS based on ∼700,000 individuals of European ancestry [41] treated BMI as a continuous metric. Hence, the causal effect of BMI on GrimEAA is positive (0.599 years, Table 6).

Moreover, several lifestyle factors presented significant causal effects on GrimEAA (Table 6; Fig. 1). Current smoking accelerated GrimAge ($\beta$ [SE]: 5.314 [1.658] years; *q* = 8.76E-3), while never smoking decelerated GrimAge ($\beta$ [SE]: − 2.981 [0.524] years; *q* = 1.63E-7). Moreover, walking for pleasure (not as a means of transport) in the last four weeks ($\beta$ [SE]: − 4.313 [1.236] years; *q* = 4.21E-3) and dried fruit intake ($\beta$ [SE] per 1-SD: − 1.523 [0.504] year; *q* = 0.0131) (Table 6; Fig. 1) also decelerated GrimAge.

Cochran's Q test suggested possible heterogeneity for BMI (p = 0.015) and dried fruit intake (*p* = 0.049; Supplementary Table S21). The MR pleiotropy residual sum and outlier (MR-PRESSO) analysis detected and excluded two outlier SNPs from the 941 BMI-associated SNPs (Table 6). Nonetheless, the updated result provided by MR-PRESSO was similar to those of the IVW method (Table 6; *β* per 1-SD increase in BMI [SE]: 0.598 [0.080] years; *q* = 3.70E-12). On the other hand, no outlier SNP was detected from the 43 SNPs associated with dried fruit intake.

The CVH factors’ causal effects on other EAA measures are presented in Supplementary Tables S14–S17 and Supplementary Figures S3–S6. Compared with five significant factors for GrimEAA (Fig. 1, *p* < 0.05 & FDR *q* < 0.05 under the IVW method), only 2, 1, 0, and 0 significant factors for PhenoEAA (Figure S5), IEAA (Figure S4), HannumEAA (Figure S3), and DNAmPAI-1 (Figure S6), respectively.

### EUR multivariable MR analysis: BMI and lifestyle factors on GrimEAA

**Fig. 2:**
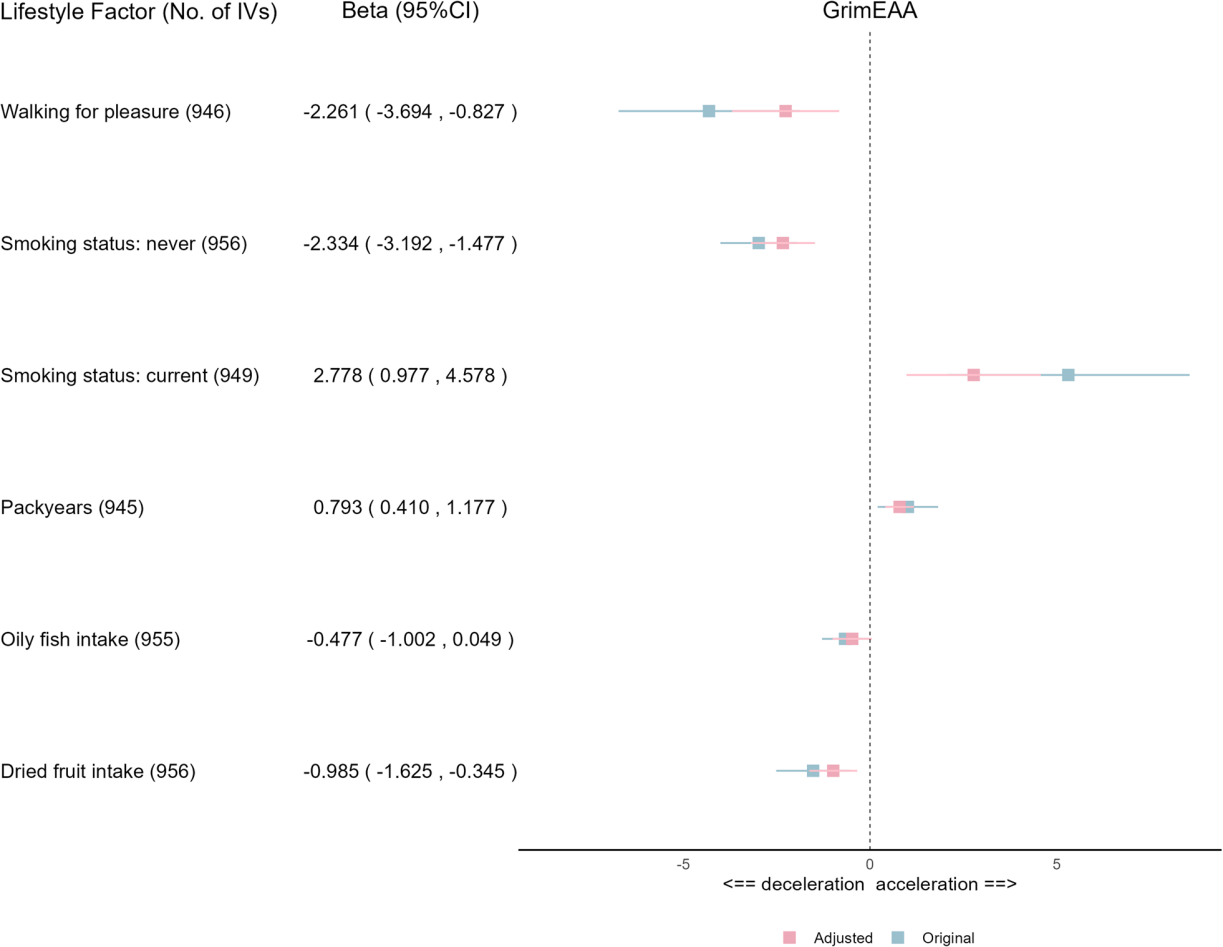
EUR Multivariable MR analysis to assess the effects of lifestyle factors on GrimEAA while adjusting for BMI Causal estimates are Beta (95% CI) in years. Blue boxes mark the original Beta (95% CI) from the IVW method, while red boxes denote the adjusted Beta (95% CI) from the IVW method. “No. of IVs” indicates the number of instruments used in the multivariable MR analysis

Our MR analysis observed a significant causal effect of BMI on GrimEAA (*q* = 2.91E-12; Table 6), PhenoEAA (*q* = 1.52E-7; Supplementary Table S16), and IEAA (*q* = 0.0477; Supplementary Table S15). After adjusting for BMI, we evaluated whether the six significant lifestyle factors (*p* < 0.05) in Table 6 had independent causal effects on GrimEAA. Adjusting BMI made the causal effects of significant lifestyle factors less remarkable (Fig. 2). “Oily fish intake” lost significance (Table 6, before adjusting for BMI, *p* = 0.0321) in the causal relationship (Fig. 2; after adjusting for BMI, 95% C.I. = [− 1.002, 0.049]). The other five significant lifestyle factors, including current smoking, never smoking, pack-years of smoking, walking for pleasure in the last four weeks, and dried fruit intake, remained significantly causally associated with GrimEAA (Fig. 2). This lends greater robustness to the causal relationship between these five lifestyle factors and GrimEAA.

**Fig. 3:**
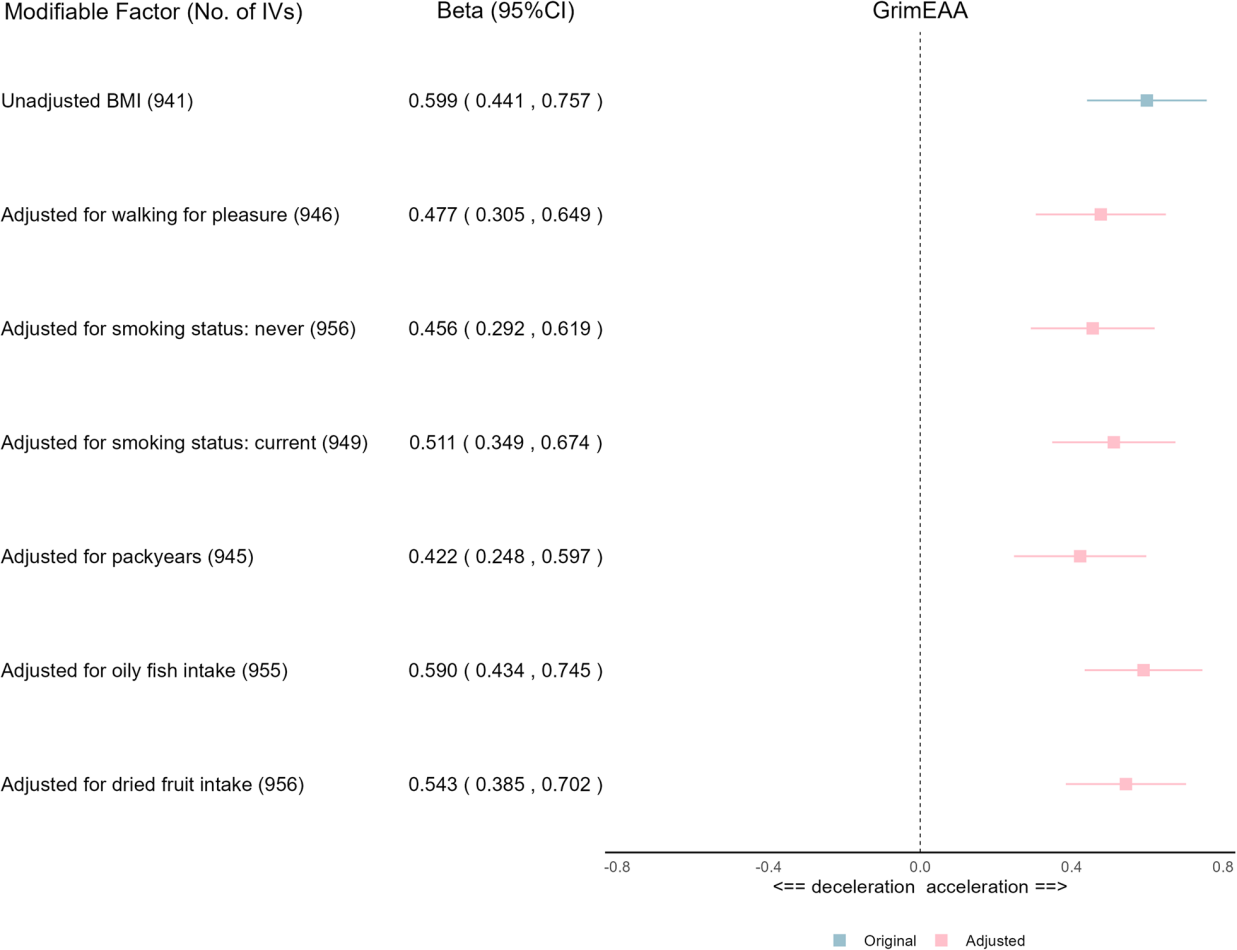
EUR Multivariable MR analysis to assess the effects of BMI on GrimEAA while adjusting for lifestyle factors Causal estimates are Beta (95% CI) in years. Blue boxes mark the original Beta (95% CI) from the IVW method, while red boxes denote the adjusted Beta (95% CI) from the IVW method. “No. of IVs” indicates the number of instruments used in the multivariable MR analysis

Despite adjusting for these lifestyle variables, BMI still presented a significant causal effect on GrimEAA ($\beta$: per 1-SD increase in BMI: 0.422–0.599 years; Fig. 3). This result reassures the causal effect of BMI on GrimEAA.

## Discussion

Previous studies have shown that ideal CVH is significantly associated with EAD [21, 32, 33, 43–47]. With the Taiwanese individual-level data and European summary statistics, we used the one- and two-sample MR approaches to show that maintaining ideal CVH was causally associated with GrimAge deceleration. When digging into CVH factors, the difference between first-generation and second-generation epigenetic clocks became even more apparent. The number of significant CVH factors in GrimEAA (5 significant factors with* q *< 0.05, Table 6) far exceeds that in the first-generation clocks (only one significant factor with *q* < 0.05 in Supplementary Tables S14, S15), indicating that GrimEAA is more likely to be affected by CVH than the first-generation clocks. It also shows that a healthy lifestyle may help slow the aging process, including maintaining an ideal BMI (*q* = 2.91E-12 for GrimEAA [Table 6] and *q* = 0.0477 for IEAA [Table S15), never smoking (*q* = 1.63E-7 for GrimEAA [Table 6]), walking for pleasure (*q* = 4.21E-3 for GrimEAA [Table 6]), and eating dried fruit (*q* = 0.0131 for GrimEAA [Table 6]).

Maintaining an ideal BMI provided the most significant causal relationship with GrimEAA (*q* = 2.91E-12 [Table 6]). BMI is a convenient measurement for general obesity. Obesity increases the risk of age-related conditions such as CVD, hypertension, T2DM, and cancer [48]. Additionally, it can reduce life expectancy by as much as 20 years [49]. Our previous work has indicated the association of obesity with GrimEAA and PhenoEAA [16], whereas we here confirm the causal relationship between them (BMI on GrimEAA [*q* = 2.91E-12, Table 6]; BMI on PhenoEAA [*q* = 1.52E-7, Table S16]).

Regarding dietary habits, only dried fruit intake presented a causal effect on GrimEAA. In the UK Biobank’s shortened food frequency touchscreen questionnaire, participants were asked, “About how many pieces of DRIED fruit would you eat per DAY? (Count one prune, one dried apricot, and ten raisins as one piece; put ‘0’ if you do not eat any).” [50] The responses had a mean of 0.899 pieces and a standard deviation of 1.826 pieces (https://biobank.ndph.ox.ac.uk/ukb/field.cgi?id=1319). We found that an increase of 1 SD (i.e., 1.826 pieces) in dried fruit intake is associated with a reduction of 1.523 years in GrimEAA (*q* = 0.0131; Table 6). The beneficial impact of eating dried fruit on slowing aging warrants further investigation.

With drying technologies, fresh fruits shrink to smaller and energy-dense dried fruits. Dried fruits such as raisins and dates provide various nutrients, including vitamins, minerals, antioxidants, and dietary fiber [51–54]. These elements may help reduce oxidative damage, regulate blood sugar, and lower the risk of T2DM and heart diseases [52, 55, 56]. A systematic review of observational studies showed that consuming dried fruits was associated with a lower cancer incidence or mortality. Eating raisins and other dried fruits may help prevent cancers related to the digestive system [57].

Recently, Kong et al*.* [36] performed a two-sample MR analysis to investigate causal associations of 19 lifestyle and metabolic factors with PhenoEAA and GrimEAA. Three of the 19 factors were also investigated in this work: BMI, diastolic blood pressure, and systolic blood pressure. Our results for these three factors were consistent with those of Kong et al*.* [36]. We also found that a larger BMI increased GrimEAA and PhenoEAA, and higher blood pressure levels enlarged PhenoEAA (but not GrimEAA).

Although the causality between ideal CVH and EAA has been demonstrated in this work, the pathways from lifestyle through body functions and how they affect aging are complicated and require additional investigation. The limitation of this study is that the power of our one-sample MR analysis may be compromised due to the relatively small sample size of DNAm data in TWB (compared with the EAA GWAS [37] in the two-sample MR analysis). Moreover, we did not identify sufficient genome-wide significant SNPs for the IVs of the SMK and PA scores. As a result, the causal effects of SMK and PA on EAA can only be inferred from the European data with the two-sample MR analysis.

In this work, we investigated the causal effects of cardiovascular health on five epigenetic clocks and one DNAm-based surrogate plasma protein of GrimAge, DNAmPAI-1. Although we have analyzed many DNAm measures, only GrimEAA and DNAmPAI-1 were found to be causally linked with the CVH score (Table 3). The subsequent analysis of individual CVH factors may be restricted to GrimEAA and DNAmPAI-1 to reduce the number of tests. Nonetheless, to provide a more complete analysis, we still put the results of insignificant EAA measures in the supplementary materials.

## Conclusions

This work looks deeply into the relationship between the CVH score and EAA. The findings indicate that the CVH score is causally related to the deceleration for GrimEAA (Table 3; *β* [SE] for each point of the CVH score: − 0.993 [0.363] years; *p* = 0.0063) and DNAmPAI-1 (Table 3; *β* [SE] for each point of the CVH score: − 0.294 [0.099] SD of DNAmPAI-1; *p* = 0.0030) in TWB, IEAA (Table 5; *β* [SE] for each point of the CVH score: -0.389 [0.186] years; *p* = 0.0360) and GrimEAA (Table 5; *β* [SE] for each point of the CVH score: − 0.526 [0.244] years; *p* = 0.0310) in European populations.

Through further TWB analysis for four CVH components, we found that the BMI score presented a causal effect on GrimEAA (Table 4; *β* [SE] for each point of the BMI score: − 2.382 [0.952] years; *q* = 0.0498) and DunedinPACE (Table 4; *β* [SE] for each point of the BMI score: − 0.097 [0.030]; *q* = 0.0044). Furthermore, the TC score exhibited a causal effect on DNAmPAI-1 (Table 4; *β* [SE] for each point of the TC score: − 0.452 [0.150] SD of DNAmPAI-1; *q* = 0.0102), which was in line with the abundant research on the relationship between TC and PAI-1 [58].

In our two-sample MR using the European data, the effect of BMI on GrimEAA did not change much after adjusting for other lifestyle factors (Fig. 3). This result indicates that maintaining an ideal BMI is particularly important for slowing aging. Furthermore, lifestyle factors such as walking for pleasure, smoking status, and dried fruit intake have a significant causal effect on GrimEAA, even after adjusting for BMI (Fig. 2). This result reveals the critical role of a healthy lifestyle in slowing aging.

## Methods

### Study Design

To assess the causality of ideal CVH and EAA, we first performed GWAS on the CVH score and individual CVH components using the TWB data. Then, a wGRS was calculated as an IV, and a one-sample MR analysis using a two-stage least-squares method was conducted [59]. Here, the CVH score and its components were considered “exposures,” whereas measures of EAA were regarded as “outcomes.” In the first stage, the exposure (i.e., the CVH score [an integer from 0 to 12], the TC score [0, 1, or 2], the FG score [0, 1, or 2], the BP score [0, 1, or 2], or the BMI score [0, 1, or 2]) was regressed on the corresponding IV (i.e., CVH-wGRS, TC-wGRS, FG-wGRS, BP-wGRS, or BMI-wGRS) using linear regression. Through this, we obtained the predicted exposure value (i.e., predicted CVH score, predicted TC score, predicted FG score, predicted BP score or predicted BMI score). In the second stage, we regressed the outcome (i.e., EAA) on the predicted exposure value (i.e., predicted CVH score, predicted TC score, predicted FG score, predicted BP score or predicted BMI score) through linear regression.

To appropriately infer the causality between the exposure (i.e., the CVH score as well as its components) and the outcome (i.e., EAA) with the MR analysis, we examined the three core assumptions: (1) relevance assumption: the IV is associated with the exposure; (2) independence assumption: the IV is not associated with any factors confounding the exposure-outcome association; and (3) exclusion restriction assumption: the IV influences the outcome only through the exposure [60]. In addition to the one-sample MR analysis, we performed a two-sample MR analysis using summary statistics from European GWAS [5, 37–42].

### Data for the one-sample MR analysis

From 2012 to 2023, the TWB recruited approximately 189,132 community-based volunteers from Taiwan’s residents. To join the TWB study, participants had to provide written informed consent. The TWB performed physical examinations for participants and collected their urine and blood samples. Among the 189,132 individuals, 147,836 had whole-genome genotyping data available. Furthermore, the lifestyle factors of each participant were recorded by a face-to-face interview with the TWB healthcare professionals [61].

The TWB performed pre-phasing and genotype imputation with SHAPEIT2 and IMPUTE2 (v2.3.1), respectively [62–64]. The reference panel included 504 East Asians (EAS) from the 1000 Genomes Phase 3 v5 and 1,451 TWB participants undergoing whole-genome sequencing. After genotype imputation, TWB researchers performed quality control procedures, including removing SNPs with missing rates > 5%, minor allele frequencies (MAF) < 0.01%, and imputation information scores < 0.3. Through these steps, ~ 9.8 million genetic variants were left in analysis.

All 147,836 TWB individuals with whole-genome genotyping data were kept in our one-sample MR analysis because their missing genotype rates were less than 10%. We excluded SNPs with the Hardy–Weinberg equilibrium test *p* < 5.7E-7 or genotyping rates < 95%. Finally, 9,804,794 SNPs passed the quality control filtering. We analyzed 3,639,571 SNPs with MAFs > 1%, a commonly used MAF cutoff in many GWAS [65]. The TWB researchers used the software “KING” (Kinship-based INference for GWAS) [66] to estimate the kinship coefficients between any two TWB individuals. We excluded individuals with more missing genotypes from each first- or second-degree relative pair. Through this procedure, 118,908 TWB participants remained in the analysis.

From 2016 to 2021, the TWB randomly selected 2,474 individuals among all TWB participants to quantify the DNAm levels from peripheral blood. This selection was based on the overall sex ratio and the population size in each region of Taiwan. Our previous work described the quality control and normalization of the DNAm data [16]. Among the 118,908 individuals with whole-genome genotyping, 2,383 had DNAm data.

For the one-sample MR analysis, the base data (used to calculate the effect sizes for the wGRS) and the target data (where the wGRS is applied) should be independent, or there may be a risk of overfitting [67]. Therefore, we used the 116,525 (= 118,908–2,383) participants (without DNAm data) as the base data and the 2,383 participants (with DNAm data) as the target data.

There are two versions of TWB questionnaires: the original version and a simplified version. Most individuals selected the simplified questionnaire to save time, in which dietary information was not collected. Therefore, in this work, we investigated the so-called “12-point CVH score” [34] based on six aspects, including TC, FG, BP, BMI, SMK, and PA. The definition for each factor is described in Table 7.

**Table 7:** Definition for the 12-point CVH score

	Poor: 0 point	Intermediate: 1 point	Ideal: 2 points
Lifestyle factors			
BMI (kg/m^2^)	BMI ≥ 27	24 ≤ BMI < 27	BMI < 24
SMK: Smoking status	Current	Former (quit < 6 months)	Never or former (quit ≥ 6 months)
PA: Physical activity (Regular exercise)	Never	Between never and regular	At least 30 min thrice a week (Regular exercise)
Clinical factors			
TC: Total cholesterol (mg/dL)	TC ≥ 240	200 ≤ TC < 240	TC < 200
FG: Fasting glucose (mg/dL)	Fasting Glucose ≥ 126	100 ≤ Fasting Glucose < 126	Fasting Glucose < 100
BP: Blood pressure (mmHg)	SBP ≥ 140 or DBP ≥ 90	(120 ≤ SBP < 140 and DBP < 90) or (80 ≤ DBP < 90 and SBP < 140)	SBP < 120 and DBP < 80

DBP: diastolic blood pressure; SBP: systolic blood pressure

To calculate epigenetic age, we uploaded the DNAm data to the Horvath laboratory's online DNAm age calculator (https://dnamage.genetics.ucla.edu/new). Five measures of epigenetic markers were used in the analysis: HannumEAA [19] (column “AgeAccelerationResidualHannum” from the DNAm Age Calculator output), IEAA [8] (column “IEAA”), PhenoEAA [22] (column “AgeAccelPheno”), GrimEAA [23, 24] (column “AgeAccelGrim”), and DNAmPAI-1 levels [23, 24] (column “DNAmPAI1”). DunedinPACE was calculated based on the R package “DunedinPACE” (https://github.com/danbelsky/DunedinPACE) [68, 69]. DNAmPAI-1 ranged from 7,292 pg/mL to 25,682 pg/mL, with a mean of 15,813 pg/mL and an SD of 2,514 pg/mL. To facilitate the interpretation of effect sizes, we performed the *z*-score transformation on DNAmPAI-1.

### Statistical Analysis for the one-sample MR analysis

We used PLINK v1.90 [70] to perform the GWAS for the CVH score and six CVH components (TC score, FG score, BP score, BMI score, SMK score, and PA score) under the common assumption of additive allelic effects of SNPs. The regression models were controlled for confounding factors of CVH-EAA association [21], including age (in years), sex (male vs. female), drinking status (yes vs. no), educational attainment (an integer from 1 to 7), and the first ten ancestry principal components. Then, we used the PLINK [70] clumping procedure to identify nearly independent significant SNPs (*p* < 5E-8) with linkage disequilibrium (LD) measure of *r*^*2*^ < 0.01 within 10,000 kilobases.

To avoid bias caused by weak IVs and to increase power [71], we combined genome-wide significant SNPs (*p* < 5E-8) to build the wGRS. We checked the three MR assumptions. F statistic > 10 is considered a strong IV for the relevance assumption [35]. Ensuring that the MR assumptions (2) and (3) hold is technically impossible. However, we can disprove them by testing the associations between wGRS, confounders, and EAA [60]. The MR assumption (2), the independence assumption, was tested using a two-sample t-test for binary confounders (sex and drinking status) or a Pearson correlation test for continuous confounders (chronological age and educational attainment) [21].

When checking the MR assumption (3), the exclusion restriction assumption, we regressed EAA on CVH-wGRS while adjusting for the corresponding exposure (i.e., the CVH score) and the known confounding factors (age, sex, drinking status, and educational attainment) [21]. We used the Wald statistic to test the significance of the CVH-wGRS’s regression coefficient. A significant regression coefficient implies that assumption (3) is violated because CVH-wGRS can affect EAA through paths other than the CVH score. Similar checks were performed for TC-wGRS, FG-wGRS, BP-wGRS, and BMI-wGRS.

We tested assumptions (2) or (3) at the standard significance level 0.05. Violations of assumptions (2) or (3) indicated that wGRS is associated with some confounding factors or the outcome. We used the Cochran-Armitage trend test [72] to assess the association between each SNP constructing wGRS and categorical confounders. For each continuous confounder, the analysis of variance was conducted to evaluate its association with SNPs. SNPs with the lowest p-value were removed until the wGRS met assumptions (2) and (3). Finally, the F statistic for checking assumption (1) was calculated.

With wGRS as an IV, we performed the one-sample MR using the two-stage least-squares method [59, 73]. The data came from the 2,383 TWB individuals with DNAm data. In the first stage, the exposure (CVH score or CVH components) was regressed on the IV (the corresponding wGRS). In the second stage, the outcome (EAA) was then regressed on the predicted exposure from the first stage. The coefficient of the predicted exposure from the second-stage regression was the causal effect of the exposure on the outcome.

### Data for the two-sample MR analysis

The summary statistics for the CVH score were obtained from a published GWAS based on the VA Million Veteran Program [5], in which the diet component was not considered because only 0.4% of the VA people had an ideal diet. Therefore, the CVH score ranged from 0 to 12, the same as our one-sample MR analysis using the TWB data. The GWAS identified 17 independent CVH-associated SNPs with *p* < 5E-8. The associations persisted even when individuals with CVD were excluded from the analysis [5].

Factors related to the three CVH clinical and four lifestyle factors were sourced from the MRC IEU Open GWAS Project (https://gwas.mrcieu.ac.uk/) [74]. Definitions of the CVH factors and the GWAS are shown in Supplementary Table S13. We extracted the summary statistics of EAA GWAS from a contemporary GWAS meta-analysis involving 34,710 European participants from 28 cohorts (Edinburgh DataShare https://datashare.ed.ac.uk/handle/10283/3645) [37].

### Statistical Analysis for the two-sample MR analysis

We used the IVW method to calculate causal estimates (β coefficients and SEs) between the CVH score and several measures of EAA for the European population. The IVW method employs a meta-analytical approach to construct a single causal estimate by combining the Wald ratio statistics from SNPs in the IV set [75]. To assess the robustness of IVW estimates and detect pleiotropy, we conducted the following analyses with three different assumptions: the Weighted Median [76], MR-Egger [77], and MR-PRESSO [78] methods.

The Weighted Median method ensures consistent causal predictions when more than half of the analytic weights are derived from valid IVs [76]. The MR-Egger method allows for intercept estimation; it can detect pleiotropy bias with limited precision [77]. The MR-PRESSO method identifies potential horizontal pleiotropy by identifying outlier SNPs. It corrects horizontal pleiotropy according to the impact of outliers on the causal estimate [78]. Cochran’s Q test was utilized to assess the heterogeneity of IVW estimates [79], and horizontal pleiotropy was identified using the p-value of the intercept in the MR-Egger model [77]. When the heterogeneity was present, a random-effects IVW model was utilized [80, 81]. Then, we did the same procedure to assess the causal effects of each CVH factor on EAA.

All analyses were performed using R (version 4.2.0). The two-sample MR analyses were conducted with the TwoSampleMR [82, 83] and MR-PRESSO [78] packages. In our selection criteria for independent genetic variants in European populations, we prioritized SNPs with a significant association exposure at a genome-wide level (*p* < 5E-08). We used the clump_data function in the TwoSampleMR package to prune dependent SNPs with a stringent LD measure of *r*^*2*^ < 0.01 within 10,000 kilobases. Multiple testing corrections were performed on CVH factors using the FDR strategy. The FDR q-values were calculated by the R command “p.adjust” with the Benjamini–Hochberg procedure [84].

To ensure the robustness of our results, we further conducted multivariable MR analysis to evaluate whether the causal effect of BMI on GrimEAA is independent of other lifestyle factors and whether the causal effect of lifestyle factors on GrimEAA is affected by BMI [85]. For example, to assess the impact of current smoking on GrimEAA while adjusting for BMI, we extracted SNPs associated with current smoking or BMI (*p* < 5E-8) as instruments. Then, we kept the independent SNPs in the sense that the LD measure of *r*^*2*^ < 0.01 within 10,000 kilobases and harmonized them to be on the same strand. These procedures were performed with the “mv_extract_exposures” and “mv_harmonise_data” functions in the TwoSampleMR [82, 83] package.

## Supplementary Information

Supplementary material 1.

## Data Availability

The datasets used and analyzed during the current study are available from https://www.twbiobank.org.tw/.

## Abbreviations

BMI: Body mass index
BP: Blood pressure
CVD: Cardiovascular disease
CpGs: Cytosine–phosphate–guanine sites
CVH: Cardiovascular health
DNAm: DNA methylation
DNAmPAI-1: DNAm-based plasminogen activator inhibitor-1
EAA: Epigenetic age acceleration
EAD: Epigenetic age deceleration
FDR: False discovery rate
FG: Fasting glucose
GrimEAA: GrimAge acceleration
GWAS: Genome-wide association studies
IEAA: Horvath’s intrinsic epigenetic age acceleration
IV: Instrumental variable
IVW: Inverse variance weighted
LD: Linkage disequilibrium
MAF: Minor allele frequency
MR: Mendelian randomization
MR-PRESSO: MR pleiotropy residual sum and outlier
PA: Physical activity
PAI-1: Plasminogen activator inhibitor-1
TWB: Taiwan Biobank
wGRS: Weighted genetic risk score
sd: Standard deviation
SMK: Smoking
SNP: Single-nucleotide polymorphism
T2DM: Type 2 diabetes mellitus
TC: Total cholesterol
