# Supplementary material for: Causal effects of cardiovascular health on five epigenetic clocks
Source: Clin Epigenetics. 2024 Sep 27;16:134. doi: 10.1186/s13148-024-01752-5 (PMC11438310; doi:10.1186/s13148-024-01752-5)
Supplement: Supplementary file 1 — Supplementary material 1. [file 13148_2024_1752_MOESM1_ESM.docx]

Supplementary Materials of

Causal effects of cardiovascular health on five epigenetic clocks

Hsien-Liang Sung ^1^, Wan-Yu Lin ^1,2^*

^1^ Institute of Health Data Analytics and Statistics, College of Public Health, National Taiwan University, Taipei, Taiwan

^2^ Master of Public Health Degree Program, College of Public Health, National Taiwan University, Taipei, Taiwan

* Corresponding author: Wan-Yu Lin, Ph.D.

Wan-Yu Lin, Ph.D. (<https://orcid.org/0000-0002-3385-4702>)

Room 501, No. 17, Xu-Zhou Road, Taipei 100, Taiwan

Phone/Fax: +886-2-33668106; E-mail: [linwy@ntu.edu.tw](mailto:linwy@ntu.edu.tw)

Contents

[Table S1. The 74 SNPs associated with the total cholesterol (TC) score (p < 5E-8) 3](#_Toc176893091)

[Table S2. The 37 SNPs associated with the fasting glucose (FG) score (p < 5E-8) 5](#_Toc176893092)

[Table S3. The 31 SNPs associated with the blood pressure (BP) score (p < 5E-8) 6](#_Toc176893093)

[Table S4. The 34 SNPs associated with the BMI score (p < 5E-8) 7](#_Toc176893094)

[Table S5. The SNP associated with the smoking (SMK) score (p < 5E-8) 7](#_Toc176893095)

[Table S6. Check for the MR assumption (2) 8](#_Toc176893096)

[Table S7. Check for the MR assumption (3) 10](#_Toc176893097)

[Table S8. The p-values of testing the associations between sex, DNAmPAI-1, and the 15 SNPs constructing CVH-wGRS originally. 13](#_Toc176893098)

[Table S9. The p-values of testing the associations between DNAmPAI1 and the 74 SNPs constructing TC-wGRS originally. 14](#_Toc176893099)

[Table S10. The p-values of testing the associations between chronological age, DNAmPAI-1 and the 37 SNPs constructing FG-wGRS originally. 16](#_Toc176893100)

[Table S11. The p-values of testing the associations between educational attainment, HannumEAA and 34 SNPs constructing BMI-wGRS originally. 17](#_Toc176893101)

[Table S12. Test for heterogeneity and pleiotropy in the causal inference of the CVH score on epigenetic markers in EUR 18](#_Toc176893102)

[Table S13. Definitions of the CVH factors in the two-sample MR analysis 19](#_Toc176893103)

[Table S14. EUR MR analysis result: the causal effects of the CVH factors on HannumEAA 20](#_Toc176893104)

[Table S15. EUR MR analysis result: the causal effects of the CVH factors on IEAA 22](#_Toc176893105)

[Table S16. EUR MR analysis result: the causal effects of the CVH factors on PhenoEAA 23](#_Toc176893106)

[Table S17. EUR MR analysis result: the causal effects of the CVH factors on DNAmPAI-1 24](#_Toc176893107)

[Table S18. Test for heterogeneity and pleiotropy in the causal inference of the CVH factors on HannumEAA in EUR 25](#_Toc176893108)

[Table S19. Test for heterogeneity and pleiotropy in the causal inference of the CVH factors on IEAA in EUR 26](#_Toc176893109)

[Table S20. Test for heterogeneity and pleiotropy in the causal inference of the CVH factors on PhenoEAA in EUR 27](#_Toc176893110)

[Table S21. Test for heterogeneity and pleiotropy in the causal inference of the CVH factors on GrimEAA in EUR 28](#_Toc176893111)

[Table S22. Test for heterogeneity and pleiotropy in the causal inference of the CVH factors on DNAmPAI-1 in EUR 29](#_Toc176893112)

[Figure S1. The Manhattan plots and the quantile-quantile (Q-Q) plots of the (12-point) CVH score, the total cholesterol (TC) score, the fasting glucose (FG) score, and the blood pressure (BP) score 30](#_Toc176893113)

[Figure S2. The Manhattan plots and the quantile-quantile (Q-Q) plots of the BMI score, the smoking (SMK) score, and the physical activity (PA) score 31](#_Toc176893114)

[Figure S3. The causal effects of CVH factors on HannumEAA in EUR. 32](#_Toc176893115)

[Figure S4. The causal effects of CVH factors on IEAA in EUR. 33](#_Toc176893116)

[Figure S5. The causal effects of CVH factors on PhenoEAA in EUR. 34](#_Toc176893117)

[Figure S6. The causal effects of CVH factors on DNAmPAI-1 in EUR. 35](#_Toc176893118)

[Reference 36](#_Toc176893119)

# Table S1. The 74 SNPs associated with the total cholesterol (TC) score (p < 5E-8)

| **CHR** | **SNP** | **A1** | **A2** | **MAF** | **BP** | **BETA** | **SE** | **p-value** |
| --- | --- | --- | --- | --- | --- | --- | --- | --- |
| 1 | rs10782936 | A | G | 0.08 | 92576349 | 0.0296 | 0.0052 | 1.03E-08 |
| 1 | rs2495500 | T | A | 0.43 | 55021995 | 0.0176 | 0.0028 | 5.50E-10 |
| 1 | rs3832016 | C | CT | 0.07 | 109275536 | 0.1020 | 0.0055 | 1.15E-75 |
| 1 | rs486142 | G | A | 0.22 | 234712862 | 0.0219 | 0.0034 | 6.24E-11 |
| 1 | rs557211 | G | T | 0.19 | 55048542 | 0.0282 | 0.0036 | 2.85E-15 |
| 1 | rs565436 | G | A | 0.10 | 55058928 | 0.0258 | 0.0046 | 2.03E-08 |
| 1 | rs6661533 | G | A | 0.27 | 25445224 | -0.0215 | 0.0032 | 1.11E-11 |
| 1 | rs867772 | A | G | 0.19 | 220799001 | 0.0282 | 0.0036 | 2.61E-15 |
| 1 | rs998403 | A | G | 0.21 | 62462878 | 0.0424 | 0.0034 | 4.32E-35 |
| 2 | rs10164853 | G | A | 0.31 | 157625480 | 0.0207 | 0.0030 | 6.94E-12 |
| 2 | rs11687710 | C | T | 0.08 | 21148196 | -0.0622 | 0.0051 | 2.54E-34 |
| 2 | rs13384733 | C | G | 0.01 | 43726614 | -0.0763 | 0.0130 | 4.07E-09 |
| 2 | rs57825321 | A | T | 0.15 | 21024193 | 0.0834 | 0.0040 | 2.77E-98 |
| 2 | rs6547692 | G | A | 0.49 | 27512105 | -0.0372 | 0.0028 | 3.32E-40 |
| 2 | rs6727888 | T | C | 0.27 | 62660749 | 0.0302 | 0.0031 | 6.67E-22 |
| 2 | rs77401561 | G | T | 0.09 | 21311543 | -0.0291 | 0.0049 | 2.86E-09 |
| 4 | rs35502934 | A | G | 0.17 | 99547078 | -0.0215 | 0.0038 | 1.13E-08 |
| 4 | rs9994887 | A | G | 0.42 | 68671757 | 0.0163 | 0.0029 | 1.28E-08 |
| 5 | rs28735189 | C | T | 0.27 | 156967082 | 0.0354 | 0.0032 | 4.14E-29 |
| 5 | rs3064191 | CTTGTA | C | 0.49 | 75343719 | 0.0410 | 0.0028 | 1.33E-48 |
| 6 | rs144194669 | C | G | 0.01 | 160375171 | -0.1032 | 0.0130 | 1.74E-15 |
| 6 | rs2859078 | G | A | 0.04 | 32734672 | -0.0455 | 0.0072 | 2.94E-10 |
| 6 | rs73596816 | A | G | 0.05 | 160596331 | -0.0636 | 0.0063 | 4.04E-24 |
| 6 | rs9380151 | C | T | 0.11 | 30052051 | 0.0247 | 0.0045 | 3.26E-08 |
| 7 | rs4917129 | C | T | 0.26 | 50283578 | -0.0186 | 0.0032 | 5.07E-09 |
| 8 | rs112784971 | T | C | 0.23 | 58493931 | 0.0274 | 0.0033 | 2.49E-16 |
| 8 | rs2737246 | C | G | 0.31 | 115647351 | 0.0216 | 0.0031 | 1.31E-12 |
| 8 | rs4921915 | A | G | 0.48 | 18414956 | 0.0195 | 0.0028 | 3.19E-12 |
| 8 | rs6982502 | C | T | 0.44 | 125467120 | -0.0301 | 0.0028 | 1.52E-26 |
| 9 | rs11789603 | T | C | 0.06 | 104884738 | -0.0434 | 0.0057 | 3.18E-14 |
| 9 | rs13289095 | T | G | 0.08 | 128704210 | 0.0318 | 0.0052 | 1.13E-09 |
| 9 | rs1883025 | T | C | 0.23 | 104902020 | 0.0491 | 0.0033 | 3.39E-50 |
| 9 | rs2519093 | T | C | 0.18 | 133266456 | -0.0525 | 0.0036 | 2.22E-47 |
| 9 | rs2740480 | G | A | 0.42 | 104800276 | 0.0204 | 0.0028 | 6.35E-13 |
| 9 | rs4149307 | C | T | 0.26 | 104827463 | 0.0328 | 0.0032 | 6.57E-25 |
| 9 | rs79638982 | G | T | 0.22 | 127963460 | -0.0236 | 0.0034 | 3.21E-12 |
| 10 | rs2001737 | T | C | 0.39 | 112236289 | 0.0201 | 0.0029 | 2.68E-12 |
| 10 | rs2297991 | T | C | 0.28 | 112153464 | -0.0233 | 0.0031 | 6.77E-14 |
| 10 | rs4919594 | G | C | 0.18 | 93063839 | -0.0204 | 0.0037 | 3.00E-08 |
| 11 | rs12718464 | A | G | 0.04 | 116836685 | 0.0463 | 0.0070 | 2.98E-11 |
| 11 | rs174570 | C | T | 0.41 | 61829740 | -0.0324 | 0.0029 | 1.10E-29 |
| 11 | rs2342387 | C | G | 0.36 | 5671209 | -0.0173 | 0.0030 | 6.41E-09 |
| 11 | rs662799 | G | A | 0.27 | 116792991 | -0.0580 | 0.0031 | 2.05E-76 |
| 11 | rs7130876 | G | A | 0.14 | 48029443 | -0.0263 | 0.0042 | 3.21E-10 |
| 11 | rs72085277 | T | TTCTG | 0.36 | 126371957 | -0.0179 | 0.0029 | 9.18E-10 |
| 12 | rs10846744 | G | C | 0.42 | 124827879 | 0.0175 | 0.0028 | 7.17E-10 |
| 12 | rs11066222 | T | G | 0.33 | 112263014 | -0.0228 | 0.0033 | 4.03E-12 |
| 12 | rs1568428 | C | T | 0.30 | 123254329 | -0.0242 | 0.0031 | 2.17E-15 |
| 12 | rs2373355 | A | G | 0.29 | 100528401 | -0.0187 | 0.0031 | 1.47E-09 |
| 13 | rs9567609 | G | A | 0.37 | 32371580 | 0.0173 | 0.0029 | 2.56E-09 |
| 14 | rs2296651 | A | G | 0.10 | 69778476 | 0.0399 | 0.0048 | 7.71E-17 |
| 15 | rs2070895 | A | G | 0.38 | 58431740 | -0.0376 | 0.0029 | 4.25E-39 |
| 15 | rs261291 | C | T | 0.41 | 58387979 | -0.0365 | 0.0028 | 1.58E-37 |
| 15 | rs6493996 | T | C | 0.19 | 58284097 | 0.0292 | 0.0036 | 3.73E-16 |
| 16 | rs117891475 | T | C | 0.03 | 72113517 | -0.1012 | 0.0086 | 5.17E-32 |
| 16 | rs183130 | T | C | 0.16 | 56957451 | -0.0414 | 0.0038 | 1.67E-27 |
| 16 | rs201375854 | A | AAGTT | 0.20 | 72862845 | 0.0202 | 0.0035 | 1.09E-08 |
| 16 | rs6499560 | C | A | 0.24 | 72113767 | -0.0231 | 0.0033 | 1.71E-12 |
| 17 | rs4794047 | T | A | 0.34 | 47685639 | -0.0211 | 0.0030 | 1.17E-12 |
| 17 | rs8077252 | C | T | 0.43 | 69088921 | -0.0225 | 0.0029 | 3.74E-15 |
| 18 | rs11082764 | G | A | 0.41 | 49593209 | -0.0373 | 0.0029 | 5.44E-39 |
| 18 | rs1943973 | G | A | 0.19 | 49653146 | 0.0313 | 0.0036 | 1.83E-18 |
| 19 | rs141622900 | A | G | 0.07 | 44923535 | 0.1743 | 0.0053 | 3.34E-232 |
| 19 | rs143020224 | G | C | 0.01 | 11076648 | 0.1056 | 0.0138 | 2.38E-14 |
| 19 | rs148673297 | A | ATTTTATTTTTTAT | 0.06 | 11068052 | -0.0491 | 0.0060 | 2.75E-16 |
| 19 | rs2278426 | T | C | 0.26 | 11239812 | 0.0543 | 0.0032 | 8.47E-65 |
| 19 | rs2738464 | G | C | 0.28 | 11131631 | 0.0523 | 0.0031 | 2.06E-62 |
| 19 | rs4802607 | C | G | 0.41 | 49456107 | 0.0212 | 0.0029 | 2.25E-13 |
| 19 | rs58542926 | T | C | 0.07 | 19268740 | 0.0310 | 0.0056 | 3.80E-08 |
| 19 | rs6857 | T | C | 0.08 | 44888997 | -0.0892 | 0.0051 | 2.56E-69 |
| 20 | rs2328223 | C | A | 0.20 | 17865277 | -0.0201 | 0.0035 | 1.01E-08 |
| 20 | rs6124341 | G | A | 0.24 | 41325964 | 0.0272 | 0.0033 | 7.05E-17 |
| 20 | rs6129629 | G | A | 0.45 | 40556638 | -0.0172 | 0.0028 | 9.63E-10 |
| 22 | rs2106430 | T | C | 0.11 | 30201146 | -0.0246 | 0.0045 | 3.19E-08 |
| 1 | rs10782936 | A | G | 0.08 | 92576349 | 0.0296 | 0.0052 | 1.03E-08 |

# Table S2. The 37 SNPs associated with the fasting glucose (FG) score (p < 5E-8)

| **CHR** | **SNP** | **A1** | **A2** | **MAF** | **BP** | **BETA** | **SE** | **p-value** |
| --- | --- | --- | --- | --- | --- | --- | --- | --- |
| 2 | rs1402837 | T | C | 0.40 | 168900844 | -0.0235 | 0.0021 | 4.49E-28 |
| 2 | rs243021 | G | A | 0.34 | 60357684 | 0.0123 | 0.0022 | 2.83E-08 |
| 2 | rs340515 | G | T | 0.44 | 44961231 | -0.0247 | 0.0021 | 1.97E-31 |
| 2 | rs6547692 | G | A | 0.49 | 27512105 | 0.0231 | 0.0021 | 4.27E-28 |
| 2 | rs75536691 | G | A | 0.04 | 164525008 | 0.0320 | 0.0056 | 9.48E-09 |
| 2 | rs78910115 | T | C | 0.47 | 172685102 | 0.0163 | 0.0021 | 8.95E-15 |
| 3 | rs140691790 | T | C | 0.20 | 152700541 | -0.0159 | 0.0026 | 1.90E-09 |
| 3 | rs34782298 | A | G | 0.24 | 185803208 | -0.0159 | 0.0025 | 1.18E-10 |
| 3 | rs7639266 | C | T | 0.30 | 23432840 | 0.0139 | 0.0023 | 1.24E-09 |
| 4 | rs72501962 | T | A | 0.34 | 1252250 | 0.0162 | 0.0022 | 2.93E-13 |
| 5 | rs465002 | C | T | 0.47 | 56512648 | 0.0124 | 0.0021 | 3.28E-09 |
| 6 | rs35261542 | A | C | 0.35 | 20675561 | -0.0318 | 0.0022 | 4.52E-47 |
| 6 | rs3765467 | A | G | 0.24 | 39065819 | 0.0148 | 0.0025 | 2.04E-09 |
| 7 | rs17168486 | T | C | 0.48 | 14858657 | -0.0213 | 0.0021 | 2.84E-24 |
| 7 | rs2191996 | G | C | 0.28 | 15117604 | 0.0128 | 0.0023 | 4.37E-08 |
| 7 | rs2908290 | A | G | 0.46 | 44176538 | -0.0180 | 0.0021 | 1.43E-17 |
| 7 | rs2971670 | T | C | 0.19 | 44186502 | -0.0282 | 0.0027 | 3.14E-26 |
| 7 | rs6967891 | T | C | 0.31 | 15025449 | 0.0242 | 0.0023 | 1.55E-26 |
| 7 | rs7778167 | A | G | 0.13 | 128211575 | -0.0236 | 0.0032 | 1.45E-13 |
| 8 | rs13262861 | A | C | 0.13 | 41651058 | 0.0192 | 0.0032 | 1.36E-09 |
| 8 | rs35859536 | T | C | 0.46 | 117179236 | 0.0275 | 0.0021 | 3.89E-39 |
| 9 | rs10811660 | A | G | 0.41 | 22134069 | 0.0304 | 0.0021 | 2.76E-46 |
| 9 | rs16920619 | C | T | 0.27 | 590735 | -0.0159 | 0.0024 | 2.05E-11 |
| 9 | rs4237150 | C | G | 0.45 | 4290085 | -0.0214 | 0.0021 | 2.52E-24 |
| 10 | rs10882106 | C | T | 0.19 | 92720577 | -0.0187 | 0.0027 | 2.40E-12 |
| 10 | rs10906100 | C | T | 0.44 | 12218020 | 0.0191 | 0.0021 | 1.05E-19 |
| 10 | rs7923442 | G | A | 0.27 | 26208775 | 0.0131 | 0.0024 | 2.85E-08 |
| 11 | rs10830963 | G | C | 0.43 | 92975544 | -0.0312 | 0.0021 | 1.66E-49 |
| 11 | rs17244499 | G | A | 0.06 | 72918901 | 0.0277 | 0.0043 | 1.91E-10 |
| 11 | rs2283228 | C | A | 0.36 | 2828300 | 0.0307 | 0.0022 | 5.82E-45 |
| 11 | rs231361 | G | A | 0.19 | 2670270 | 0.0173 | 0.0027 | 1.18E-10 |
| 11 | rs28456 | A | G | 0.42 | 61822009 | -0.0154 | 0.0021 | 6.98E-13 |
| 13 | rs2067296 | AAGAC | A | 0.22 | 32979946 | -0.0197 | 0.0025 | 1.07E-14 |
| 15 | rs16968809 | C | T | 0.42 | 77379776 | -0.0128 | 0.0021 | 1.59E-09 |
| 15 | rs8037894 | C | G | 0.34 | 62102065 | 0.0201 | 0.0022 | 9.76E-20 |
| 16 | rs8063057 | C | T | 0.12 | 53778521 | -0.0206 | 0.0032 | 9.00E-11 |
| 20 | rs11477526 | A | AT | 0.13 | 22574444 | 0.0226 | 0.0031 | 2.44E-13 |

# Table S3. The 31 SNPs associated with the blood pressure (BP) score (p < 5E-8)

| **CHR** | **SNP** | **A1** | **A2** | **MAF** | **BP** | **BETA** | **SE** | **p-value** |
| --- | --- | --- | --- | --- | --- | --- | --- | --- |
| 2 | chr2:61296261_AAAAT_A | AAAAT | A | 0.37 | 61296261 | 0.0174 | 0.0029 | 2.00E-09 |
| 2 | rs10200835 | C | A | 0.18 | 161831529 | 0.0205 | 0.0036 | 1.84E-08 |
| 2 | rs35021474 | G | C | 0.26 | 26693976 | 0.0259 | 0.0032 | 9.86E-16 |
| 2 | rs6547692 | G | A | 0.49 | 27512105 | -0.0159 | 0.0028 | 1.53E-08 |
| 2 | rs73029563 | C | G | 0.43 | 164151656 | 0.0367 | 0.0029 | 5.93E-38 |
| 2 | rs77559470 | G | T | 0.46 | 163587846 | 0.0166 | 0.0029 | 7.42E-09 |
| 3 | rs79991967 | G | GT | 0.27 | 53527269 | -0.0219 | 0.0032 | 5.96E-12 |
| 4 | rs16998073 | T | A | 0.42 | 80263187 | -0.0473 | 0.0028 | 7.48E-62 |
| 4 | rs3215177 | G | GA | 0.31 | 120268174 | 0.0177 | 0.0031 | 6.62E-09 |
| 4 | rs6817516 | A | C | 0.39 | 110434534 | -0.0208 | 0.0029 | 5.51E-13 |
| 5 | rs1036190 | G | A | 0.27 | 149008049 | 0.0178 | 0.0032 | 1.82E-08 |
| 5 | rs1173727 | T | C | 0.33 | 32830415 | 0.0204 | 0.0030 | 7.44E-12 |
| 5 | rs17149944 | G | A | 0.50 | 123120403 | 0.0257 | 0.0028 | 8.77E-20 |
| 6 | rs7692 | A | C | 0.30 | 43336269 | -0.0216 | 0.0031 | 1.64E-12 |
| 7 | rs13243033 | T | C | 0.38 | 27208754 | 0.0177 | 0.0029 | 1.47E-09 |
| 8 | rs17054793 | T | C | 0.39 | 26038894 | -0.0192 | 0.0029 | 5.33E-11 |
| 8 | rs62525059 | A | G | 0.30 | 142901545 | 0.0170 | 0.0031 | 3.55E-08 |
| 10 | chr10:94266427_CA_C | CA | C | 0.45 | 94266427 | -0.0198 | 0.0028 | 2.26E-12 |
| 10 | rs11191548 | C | T | 0.29 | 103086421 | 0.0298 | 0.0031 | 3.74E-22 |
| 10 | rs7075062 | C | T | 0.14 | 113967473 | -0.0253 | 0.0041 | 4.64E-10 |
| 11 | rs11230729 | T | C | 0.41 | 61511446 | 0.0208 | 0.0029 | 7.52E-13 |
| 11 | rs415895 | G | C | 0.42 | 9748015 | -0.0171 | 0.0028 | 1.80E-09 |
| 11 | rs4754698 | C | G | 0.48 | 100761177 | 0.0190 | 0.0028 | 1.53E-11 |
| 11 | rs7938342 | T | A | 0.22 | 1866576 | 0.0233 | 0.0034 | 5.54E-12 |
| 12 | rs10850432 | T | C | 0.06 | 109593246 | -0.0374 | 0.0062 | 1.23E-09 |
| 12 | rs12579302 | G | A | 0.31 | 89656726 | 0.0341 | 0.0030 | 3.03E-29 |
| 12 | rs2579290 | G | A | 0.44 | 20017604 | -0.0172 | 0.0028 | 1.50E-09 |
| 15 | rs1378940 | A | C | 0.18 | 74791153 | 0.0279 | 0.0037 | 3.61E-14 |
| 17 | rs28406364 | T | C | 0.24 | 49377145 | -0.0194 | 0.0033 | 5.36E-09 |
| 17 | rs9905385 | A | G | 0.28 | 61420889 | 0.0193 | 0.0031 | 6.36E-10 |
| 20 | rs6108787 | T | G | 0.45 | 10986566 | 0.0159 | 0.0028 | 1.91E-08 |

# Table S4. The 34 SNPs associated with the BMI score (p < 5E-8)

| **CHR** | **SNP** | **A1** | **A2** | **MAF** | **BP** | **BETA** | **SE** | **p-value** |
| --- | --- | --- | --- | --- | --- | --- | --- | --- |
| 1 | rs543874 | G | A | 0.15 | 177920345 | -0.0349 | 0.0044 | 4.03E-15 |
| 2 | rs5829949 | T | TC | 0.41 | 24913321 | -0.0263 | 0.0033 | 7.95E-16 |
| 2 | rs71417307 | G | T | 0.17 | 104138509 | -0.0237 | 0.0043 | 3.82E-08 |
| 2 | rs7576224 | G | A | 0.25 | 51065295 | 0.0218 | 0.0038 | 7.27E-09 |
| 2 | rs7585056 | A | G | 0.07 | 631528 | 0.0576 | 0.0062 | 1.94E-20 |
| 3 | rs1543683 | T | G | 0.09 | 61668052 | -0.0370 | 0.0056 | 3.88E-11 |
| 4 | rs13130484 | T | C | 0.26 | 45173674 | -0.0275 | 0.0036 | 4.58E-14 |
| 4 | rs6839738 | T | C | 0.09 | 151682691 | -0.0363 | 0.0055 | 4.75E-11 |
| 5 | rs10063803 | A | C | 0.44 | 177240326 | 0.0179 | 0.0032 | 3.52E-08 |
| 5 | rs34215404 | C | T | 0.40 | 96526002 | -0.0233 | 0.0033 | 1.28E-12 |
| 6 | rs10807036 | G | A | 0.09 | 28638620 | 0.0403 | 0.0056 | 7.80E-13 |
| 6 | rs2744475 | G | C | 0.35 | 50817167 | -0.0298 | 0.0034 | 7.83E-19 |
| 6 | rs67131976 | T | C | 0.35 | 20686647 | 0.0191 | 0.0034 | 1.34E-08 |
| 9 | rs10816778 | T | C | 0.24 | 109180729 | 0.0209 | 0.0038 | 2.87E-08 |
| 9 | rs10993127 | A | G | 0.39 | 94266397 | 0.0185 | 0.0033 | 1.67E-08 |
| 9 | rs5900713 | C | CT | 0.44 | 126636057 | 0.0219 | 0.0032 | 1.39E-11 |
| 10 | rs11191531 | C | G | 0.29 | 103047141 | -0.0224 | 0.0035 | 1.97E-10 |
| 10 | rs9415676 | G | A | 0.34 | 63250866 | 0.0191 | 0.0034 | 1.89E-08 |
| 11 | rs11030099 | A | C | 0.49 | 27656036 | 0.0177 | 0.0032 | 3.04E-08 |
| 12 | rs7132908 | A | G | 0.21 | 49869365 | -0.0219 | 0.0039 | 2.88E-08 |
| 13 | rs4477562 | T | C | 0.24 | 53530833 | -0.0204 | 0.0037 | 4.50E-08 |
| 14 | rs57933514 | A | G | 0.07 | 35560917 | 0.0432 | 0.0064 | 1.19E-11 |
| 15 | rs144047837 | T | C | 0.19 | 62084197 | 0.0266 | 0.0041 | 8.52E-11 |
| 15 | rs6493202 | A | G | 0.33 | 46249093 | -0.0202 | 0.0034 | 3.71E-09 |
| 15 | rs80032853 | CA | C | 0.42 | 67835505 | -0.0229 | 0.0033 | 3.12E-12 |
| 16 | rs12599169 | T | C | 0.27 | 20237087 | 0.0300 | 0.0036 | 1.67E-16 |
| 16 | rs1421085 | C | T | 0.13 | 53767042 | -0.0678 | 0.0048 | 1.29E-45 |
| 16 | rs2238435 | G | C | 0.32 | 3964281 | -0.0195 | 0.0034 | 1.31E-08 |
| 17 | rs7223643 | A | G | 0.34 | 67834166 | 0.0190 | 0.0034 | 2.20E-08 |
| 18 | rs17066842 | A | G | 0.02 | 60373391 | 0.0707 | 0.0105 | 1.81E-11 |
| 18 | rs6567160 | C | T | 0.18 | 60161902 | -0.0419 | 0.0042 | 6.28E-24 |
| 19 | rs2302382 | A | C | 0.06 | 45669311 | -0.0434 | 0.0070 | 5.43E-10 |
| 19 | rs4808136 | A | G | 0.23 | 18508057 | -0.0218 | 0.0038 | 7.48E-09 |
| 21 | rs13053080 | T | C | 0.43 | 38940283 | -0.0228 | 0.0032 | 1.98E-12 |

# Table S5. The SNP associated with the smoking (SMK) score (p < 5E-8)

| **CHR** | **SNP** | **A1** | **A2** | **MAF** | **BP** | **BETA** | **SE** | **p-value** |
| --- | --- | --- | --- | --- | --- | --- | --- | --- |
| 12 | rs11066132 | T | C | 0.28 | 112030402 | -0.0238 | 0.0034 | 3.35e-12 |

*Note:* CHR: chromosome; SNP: single-nucleotide polymorphism; MAF: minor allele frequency; BP: base pair.

# Table S6. Check for the MR assumption (2)

| P-value in each cell denotes the correlation between wGRS and the confounding factor | **Chronological age** | **SEX** | **Educational**  **attainment** | **Drinking status** |
| --- | --- | --- | --- | --- |
| CVH-wGRS  (15 SNPs) | 0.0607 | **0.0176** | 0.7126 | 0.2292 |
| CVH-wGRS  (14 SNPs) (Checking assumption (2), we removed rs13306194 from the original 15 SNPs) | 0.1258 | 0.0799 | 0.4984 | 0.2423 |
| CVH-wGRS  (13 SNPs) (Checking assumption (3), we removed rs662799 from the abovementioned 14 SNPs) | 0.2098 | 0.0688 | 0.5149 | 0.3131 |
| TC-wGRS  (74 SNPs) | 0.4193 | 0.0815 | 0.4703 | 0.4111 |
| TC-wGRS  (68 SNPs) (Checking assumption (3), we removed rs35502934, rs6982502, rs2297991, rs662799, rs2278426, and rs2106430 from the original 74 SNPs) | 0.5222 | 0.0691 | 0.4444 | 0.3574 |
| FG-wGRS  (37 SNPs) | **0.0130** | 0.2150 | 0.4360 | 0.1837 |
| FG-wGRS  (33 SNPs) (Checking assumption (2), we removed rs6547692, rs1402837, rs3765467, and rs8063057 from the original 37 SNPs) | 0.0640 | 0.1254 | 0.2008 | 0.1683 |
| FG-wGRS  (32 SNPs) (Checking assumption (3), we removed rs6967891 from the abovementioned 33 SNPs) | 0.0650 | 0.1540 | 0.1275 | 0.2484 |
| BP-wGRS  (31 SNPs) | 0.6433 | 0.4017 | 0.7256 | 0.9667 |
| BMI-wGRS  (34 SNPs) | 0.1421 | 0.0628 | **0.0071** | 0.7884 |
| BMI-wGRS  (32 SNPs) (Checking assumption (2), we removed rs67131976 and rs1421085 from the original 34 SNPs) | 0.3905 | 0.1139 | 0.0749 | 0.5031 |
| BMI-wGRS  (28 SNPs) (Checking assumption (3), we removed rs4477562, rs6567160, rs17066842, and rs4808136 from the abovementioned 32 SNPs) | 0.5023 | 0.1391 | 0.2520 | 0.9761 |
| *Note:* wGRS: the weighted genetic risk score; CVH: cardiovascular health; TC: total cholesterol; FG: fasting glucose; BP: blood pressure; BMI: body mass index; SMK: smoking status; DS: diet type; Educational attainment:1 represented no formal education and illiterate; 2 represented self-study and literate; 3 represented primary school; 4 represented junior high school; 5 represented senior high school; 6 represented undergraduate; 7 represented graduate or above; Bold fonts represent violations of the MR assumption (2).  The MR assumption (2) (the independence assumption) was tested using a two-sample t-test for known categorical confounders (sex and drinking status) and a Pearson correlation test for known continuous confounders (chronological age and educational attainment). | | | | |

# Table S7. Check for the MR assumption (3)

| **Exposure** | **Outcome** | **IV** | **Beta** | **SE** | **P-value** |
| --- | --- | --- | --- | --- | --- |
| CVH Score | HannumEAA | CVH-wGRS  (14 SNPs) (14 SNPs remaining through assumption (2)) | -0.3204 | 0.5160 | 0.5347 |
| CVH Score | IEAA | CVH-wGRS  (14 SNPs) (14 SNPs remaining through assumption (2)) | -0.0172 | 0.5262 | 0.9740 |
| CVH Score | PhenoEAA | CVH-wGRS  (14 SNPs) (14 SNPs remaining through assumption (2)) | 0.1359 | 0.6936 | 0.87447 |
| CVH Score | GrimEAA | CVH-wGRS  (14 SNPs) (14 SNPs remaining through assumption (2)) | -0.4686 | 0.4362 | 0.2828 |
| CVH Score | DNAmPAI-1 | CVH-wGRS  (14 SNPs) (14 SNPs remaining through assumption (2)) | -0.2356 | 0.1195 | **0.0488** |
| CVH Score | DNAmPAI-1 | CVH -wGRS  (13 SNPs) (rs662799 removed from the abovementioned 14 SNPs) | -0.1119 | 0.1262 | 0.3755 |
| CVH Score | DunedinPACE | CVH-wGRS  (14 SNPs) (14 SNPs remaining through assumption (2)) | -0.0024 | 0.0143 | 0.8695 |
| TC Score | HannumEAA | TC-wGRS  (74 SNPs) | -0.0144 | 0.4626 | 0.9751 |
| TC Score | IEAA | TC-wGRS  (74 SNPs) | -0.3069 | 0.4662 | 0.5104 |
| TC Score | PhenoEAA | TC-wGRS  (74 SNPs) | -0.0728 | 0.6193 | 0.9064 |
| TC Score | GrimEAA | TC-wGRS  (74 SNPs) | -0.1285 | 0.3988 | 0.7473 |
| TC Score | DNAmPAI-1 | TC-wGRS  (74 SNPs) | -0.3262 | 0.1104 | **0.0032** |
| TC Score | DNAmPAI-1 | TC-wGRS  (68 SNPs) (rs35502934, rs6982502, rs2297991, rs662799, rs2278426, rs2106430 removed from the original 74 SNPs) | -0.2190 | 0.1149 | 0.0568 |
| TC Score | DunedinPACE | TC-wGRS  (74 SNPs) | 0.0001 | 0.0131 | 0.9949 |
| FG Score | HannumEAA | FG-wGRS  (33 SNPs) (33 SNPs remaining through assumption (2)) | 0.9327 | 1.0050 | 0.3535 |
| FG Score | IEAA | FG-wGRS  (33 SNPs) (33 SNPs remaining through assumption (2)) | 1.0580 | 1.0220 | 0.3007 |
| FG Score | PhenoEAA | FG-wGRS  (33 SNPs) (33 SNPs remaining through assumption (2)) | 0.4450 | 1.3327 | 0.7385 |
| FG Score | GrimEAA | FG-wGRS  (33 SNPs) (33 SNPs remaining through assumption (2)) | 0.9434 | 0.8648 | 0.2754 |
| FG Score | DNAmPAI-1 | FG-wGRS  (33 SNPs) (33 SNPs remaining through assumption (2)) | 0.4862 | 0.2383 | **0.0414** |
| FG Score | DNAmPAI-1 | FG-wGRS  (32 SNPs) (rs6967891 removed from the abovementioned 33 SNPs) | 0.3826 | 0.2456 | 0.1194 |
| FG Score | DunedinPACE | FG-wGRS  (33 SNPs) (33 SNPs remaining through assumption (2)) | 0.0111 | 0.0280 | 0.6920 |
| BP Score | HannumEAA | BP-wGRS  (31 SNPs) | 0.2874 | 0.9055 | 0.7510 |
| BP Score | IEAA | BP-wGRS  (31 SNPs) | 0.0941 | 0.9243 | 0.9189 |
| BP Score | PhenoEAA | BP-wGRS  (31 SNPs) | 0.0011 | 1.2244 | 0.9993 |
| BP Score | GrimEAA | BP-wGRS  (31 SNPs) | 0.0028 | 0.8024 | 0.9972 |
| BP Score | DNAmPAI-1 | BP-wGRS  (31 SNPs) | 0.1455 | 0.2198 | 0.5080 |
| BP Score | DunedinPACE | BP-wGRS  (31 SNPs) | 0.0285 | 0.0260 | 0.2743 |
| BMI Score | HannumEAA | BMI-wGRS  (32 SNPs) (32 SNPs remaining through assumption (2)) | -2.1039 | 0.8761 | **0.0164** |
| BMI Score | HannumEAA | BMI-wGRS  (28 SNPs) (rs4477562, rs6567160, rs17066842, rs4808136 removed from the abovementioned 32 SNPs) | -1.8540 | 0.9577 | 0.0530 |
| BMI Score | IEAA | BMI-wGRS  (32 SNPs) (32 SNPs remaining through assumption (2)) | -0.5509 | 0.8896 | 0.5358 |
| BMI Score | PhenoEAA | BMI-wGRS  (32 SNPs) (32 SNPs remaining through assumption (2)) | -1.9393 | 1.1783 | 0.1000 |
| BMI Score | GrimEAA | BMI-wGRS  (32 SNPs) (32 SNPs remaining through assumption (2)) | -1.2663 | 0.7622 | 0.0968 |
| BMI Score | DNAmPAI-1 | BMI-wGRS  (32 SNPs) (32 SNPs remaining through assumption (2)) | 0.0403 | 0.2059 | 0.8447 |
| BMI Score | DunedinPACE | BMI-wGRS  (32 SNPs) (32 SNPs remaining through assumption (2)) | -0.0473 | 0.0244 | 0.0529 |
| *Note:* wGRS: the weighted genetic risk score; CVH score: cardiovascular health score; TC score: ideal total cholesterol score; FG score: ideal fasting glucose score; BP score: ideal blood pressure score; BMI score: ideal body mass index score; SMK score: ideal smoking status score; DS score: ideal diet type score; HannumEAA: the Hannum clock's measurement of epigenetic-age acceleration; IEAA: the Horvath’s clock's measurement of epigenetic-age acceleration; PhenoEAA: the PhenoAge clock's measurement of epigenetic-age acceleration; GrimEAA: the GrimAge clock's measurement of epigenetic-age acceleration; DNAm PAI-1: the DNA methylation proxies for plasminogen activator inhibitor-1; Bold fonts represent violations of the MR assumption (3).  The MR assumption (3) (the exclusion restriction assumption) was tested by regressing the outcome (e.g., HannumEAA) on IV (e.g., CVH-wGRS) while adjusting for the exposure (e.g., the CVH score), chronological age, sex, drinking status, and educational attainment. Beta is the regression coefficient of IV, and P-value is the statistical significance of Beta. | | | | | |

# Table S8. The p-values of testing the associations between sex, DNAmPAI-1, and the 15 SNPs constructing CVH-wGRS originally.

| **SNP** | **Sex** | **DNAmPAI-1** |
| --- | --- | --- |
| rs629301 | 0.7840 ^1^ | 0.9950 ^2^ |
| **rs13306194** | **0.0091** | 0.3799 |
| rs4709395 | 0.6594 | 0.4745 |
| rs73596816 | 0.2813 | 0.0457 |
| rs2908286 | 0.9810 | 0.3694 |
| rs2519093 | 0.8540 | 0.4497 |
| rs72643557 | 0.7384 | 0.9384 |
| **rs662799** | 0.8661 | **0.0008** |
| rs10550903 | 0.5722 | 0.1375 |
| rs72805612 | 0.0694 | 0.0292 |
| rs11082764 | 0.1695 | 0.5024 |
| rs3745683 | 0.4366 | 0.0743 |
| rs12972970 | 0.2846 | 0.0732 |
| rs141622900 | 0.4149 | 0.4596 |
| rs7246757 | 0.4331 | 0.9103 |
| *Note:* Bold fonts represent SNPs that were eliminated from CVH-wGRS. | | |

^1^ 0.7840 was the p-value of Cochran-Armitage test between rs629301 and sex.

^2^ 0.9950 was the p-value of regressing DNAmPAI-1 on rs629301 while adjusting for the CVH score, chronological age, sex, drinking status, and educational attainment.

# Table S9. The p-values of testing the associations between DNAmPAI1 and the 74 SNPs constructing TC-wGRS originally.

| **SNP** | **DNAmPAI1** |
| --- | --- |
| rs6661533 | 0.4736 ^1^ |
| rs2495500 | 0.8410 |
| rs557211 | 0.1984 |
| rs565436 | 0.7776 |
| rs998403 | 0.4603 |
| rs10782936 | 0.7207 |
| rs3832016 | 0.7975 |
| rs867772 | 0.6294 |
| rs486142 | 0.0617 |
| rs57825321 | 0.2295 |
| rs11687710 | 0.2119 |
| rs77401561 | 0.6362 |
| rs6547692 | 0.0347 |
| rs13384733 | 0.5119 |
| rs6727888 | 0.7637 |
| rs10164853 | 0.5055 |
| rs9994887 | 0.0277 |
| **rs35502934** | **0.0032** |
| rs3064191 | 0.8089 |
| rs28735189 | 0.6944 |
| rs9380151 | 0.1005 |
| rs2859078 | 0.9155 |
| rs144194669 | 0.4758 |
| rs73596816 | 0.0950 |
| rs4917129 | 0.2849 |
| rs4921915 | 0.8831 |
| rs112784971 | 0.0237 |
| rs2737246 | 0.5376 |
| **rs6982502** | **0.0114** |
| rs2740480 | 0.1861 |
| rs4149307 | 0.7192 |
| rs11789603 | 0.2609 |
| rs1883025 | 0.6471 |
| rs79638982 | 0.5756 |
| rs13289095 | 0.2256 |
| rs2519093 | 0.4577 |
| rs4919594 | 0.5153 |
| **rs2297991** | **0.0158** |
| rs2001737 | 0.1471 |
| rs2342387 | 0.6010 |
| rs7130876 | 0.7078 |
| rs174570 | 0.2309 |
| **rs662799** | **0.0024** |
| rs12718464 | 0.3767 |
| rs72085277 | 0.4481 |
| rs2373355 | 0.5535 |
| rs11066222 | 0.8459 |
| rs1568428 | 0.3789 |
| rs10846744 | 0.2359 |
| rs9567609 | 0.2694 |
| rs2296651 | 0.5084 |
| rs6493996 | 0.3204 |
| rs261291 | 0.2852 |
| rs2070895 | 0.1595 |
| rs183130 | 0.2676 |
| rs117891475 | 0.1309 |
| rs6499560 | 0.8684 |
| rs201375854 | 0.7441 |
| rs4794047 | 0.4340 |
| rs8077252 | 0.3726 |
| rs11082764 | 0.5458 |
| rs1943973 | 0.8475 |
| rs148673297 | 0.7095 |
| rs143020224 | 0.5484 |
| rs2738464 | 0.1565 |
| **rs2278426** | **0.0165** |
| rs58542926 | 0.4878 |
| rs6857 | 0.1320 |
| rs141622900 | 0.1798 |
| rs4802607 | 0.4943 |
| rs2328223 | 0.6949 |
| rs6129629 | 0.0919 |
| rs6124341 | 0.3345 |
| **rs2106430** | **0.0114** |
| *Note:* Bold fonts represent SNPs that were eliminated from TC-wGRS. | |

^1^ 0.4736 was the p-value of regressing DNAmPAI-1 on rs6661533 while adjusting for the TC score, chronological age, sex, drinking status, and educational attainment.

# Table S10. The p-values of testing the associations between chronological age, DNAmPAI-1 and the 37 SNPs constructing FG-wGRS originally.

| **SNP** | **Chronological age** | **DNAmPAI-1** |
| --- | --- | --- |
| **rs6547692** | **0.0099** ^1^ | 0.0071 ^2^ |
| rs340515 | 0.8566 | 0.5350 |
| rs243021 | 0.2702 | 0.8509 |
| rs75536691 | 0.5160 | 0.7453 |
| **rs1402837** | **0.0400** | 0.4783 |
| rs78910115 | 0.1217 | 0.1840 |
| rs7639266 | 0.0959 | 0.3239 |
| rs140691790 | 0.9463 | 0.6520 |
| rs34782298 | 0.5807 | 0.2183 |
| rs72501962 | 0.3174 | 0.0955 |
| rs465002 | 0.3997 | 0.8333 |
| rs35261542 | 0.4578 | 0.3361 |
| **rs3765467** | **0.0499** | 0.4524 |
| rs17168486 | 0.2660 | 0.2489 |
| **rs6967891** | 0.3650 | **0.0248** |
| rs2191996 | 0.2733 | 0.2134 |
| rs2908290 | 0.7282 | 0.8178 |
| rs2971670 | 0.5555 | 0.1613 |
| rs7778167 | 0.4459 | 0.1460 |
| rs13262861 | 0.7318 | 0.7238 |
| rs35859536 | 0.4378 | 0.8643 |
| rs16920619 | 0.6637 | 0.3293 |
| rs4237150 | 0.0530 | 0.5662 |
| rs10811660 | 0.9275 | 0.1072 |
| rs10906100 | 0.0838 | 0.9903 |
| rs7923442 | 0.4945 | 0.6842 |
| rs10882106 | 0.1264 | 0.5409 |
| rs231361 | 0.0700 | 0.6726 |
| rs2283228 | 0.4070 | 0.4954 |
| rs28456 | 0.7820 | 0.2271 |
| rs17244499 | 0.6308 | 0.5887 |
| rs10830963 | 0.8579 | 0.2326 |
| rs2067296 | 0.5662 | 0.9240 |
| rs8037894 | 0.9532 | 0.4764 |
| rs16968809 | 0.0886 | 0.9365 |
| **rs8063057** | **0.0370** | 0.0401 |
| rs11477526 | 0.5672 | 0.4892 |
| *Note*: Bold fonts represent SNPs that were eliminated from FG-wGRS. | | |

^1^ 0.0099 was the p-value of regressing chronological age on rs6547692.

^2^ 0.0071 was the p-value of regressing DNAmPAI-1 on rs6547692 while adjusting for the FG score, chronological age, sex, drinking status, and educational attainment.

# Table S11. The p-values of testing the associations between educational attainment, HannumEAA and 34 SNPs constructing BMI-wGRS originally.

| **SNP** | **Educational attainment** | **HannumEAA** |
| --- | --- | --- |
| rs543874 | 0.9857 ^1^ | 0.2120 ^2^ |
| rs7585056 | 0.1200 | 0.3179 |
| rs5829949 | 0.0644 | 0.1007 |
| rs7576224 | 0.8862 | 0.7733 |
| rs71417307 | 0.7315 | 0.6229 |
| rs1543683 | 0.4439 | 0.4787 |
| rs13130484 | 0.9155 | 0.8828 |
| rs6839738 | 0.3273 | 0.1498 |
| rs34215404 | 0.4745 | 0.7527 |
| rs10063803 | 0.9817 | 0.1677 |
| **rs67131976** | **0.0192** | 0.1505 |
| rs10807036 | 0.6567 | 0.4529 |
| rs2744475 | 0.8881 | 0.7310 |
| rs10993127 | 0.8944 | 0.1001 |
| rs10816778 | 0.3383 | 0.5754 |
| rs5900713 | 0.6132 | 0.4381 |
| rs9415676 | 0.1877 | 0.8365 |
| rs11191531 | 0.4074 | 0.9805 |
| rs11030099 | 0.6160 | 0.7722 |
| rs7132908 | 0.6575 | 0.3105 |
| **rs4477562** | 0.9471 | **0.0264** |
| rs57933514 | 0.1314 | 0.3505 |
| rs6493202 | 0.0769 | 0.1058 |
| rs144047837 | 0.5458 | 0.8307 |
| rs80032853 | 0.1137 | 0.1836 |
| rs2238435 | 0.2093 | 0.6287 |
| rs12599169 | 0.2710 | 0.2098 |
| **rs1421085** | **0.0246** | 0.2084 |
| rs7223643 | 0.4596 | 0.6985 |
| **rs6567160** | 0.1846 | **0.0402** |
| **rs17066842** | 0.9762 | **0.0430** |
| **rs4808136** | 0.2729 | **0.0047** |
| rs2302382 | 0.9737 | 0.1456 |
| rs13053080 | 0.8184 | 0.9204 |
| *Note*: Bold fonts represent SNPs that were eliminated from BMI-wGRS. | | |

^1^ 0.9857 was the p-value of regressing educational attainment on rs543874.

^2^ 0.2120 was the p-value of regressing HannumEAA on rs543874 while adjusting for the BMI score, chronological age, sex, drinking status, and educational attainment.

# Table S12. Test for heterogeneity and pleiotropy in the causal inference of the CVH score on epigenetic markers in EUR

|  | Pleiotropy test | | Heterogeneity test | |
| --- | --- | --- | --- | --- |
|  | MR-Egger intercept | p-value | Q statistic | p-value |
| CVH score | | | | |
| HannumEAA (years) | -0.412 | 0.304 | 3.927 | 0.951 |
| IEAA (years) | -0.003 | 0.930 | 9.582 | 0.478 |
| PhenoEAA (years) | -0.050 | 0.359 | 11.415 | 0.326 |
| GrimEAA (years) | 0.011 | 0.840 | 17.804 | 0.058 |
| DNAmPAI-1 (pg/ml) | 0.090 | 0.520 | 11.678 | 0.307 |
| CVH score (without CVD) | | | | |
| HannumEAA (years) | -0.038 | 0.286 | 4.059 | 0.945 |
| IEAA (years) | -0.004 | 0.907 | 9.590 | 0.477 |
| PhenoEAA (years) | -0.050 | 0.306 | 11.657 | 0.310 |
| GrimEAA (years) | 0.004 | 0.941 | 17.893 | 0.057 |
| DNAmPAI-1 (pg/ml) | 0.061 | 0.621 | 11.707 | 0.305 |
| *Note:* IEAA: Horvath’s intrinsic epigenetic age acceleration; CVH: cardiovascular health; CVD: cardiovascular disease; Bold font indicates the possible presence of heterogeneity or pleiotropy. | | | | |

# Table S13. Definitions of the CVH factors in the two-sample MR analysis

| **CVH factor** | **GWAS ID** | **Sample size** | **Unit** | **P threshold** | **Population** | **Authors** |
| --- | --- | --- | --- | --- | --- | --- |
| Clinical Factor | | | | | | |
| Diastolic blood pressure | ieu-b-39 | 757,601 | mm Hg | P<5x${10}^{-8}$ | European | Evangelou, E et al.[1] |
| Systolic blood pressure | ieu-b-38 | 757,601 | mm Hg | P<5x${10}^{-8}$ | European | Evangelou, E et al [1] |
| Total cholesterol | ieu-a-301 | 187,365 | SD (mg/dL) | P<5x${10}^{-8}$ | European (82%),  African (9%), Asian (9%) | Willer CJ et al [2] |
| Fasting glucose | ebi-a-GCST90002232 | 200,622 | SD (1mmol/L) | P<5x${10}^{-8}$ | European | Chen J et al [3] |
| Lifestyle Factor | | | | | | |
| Body mass index | ieu-b-40 | 681,275 | SD (kg/m²) | P<5x${10}^{-8}$ | European | Yengo, L et al [4] |
| Smoking status | | | | | | |
| Current | ukb-a-225 | case 33,928  control 302,096 | logOR | P<5x${10}^{-8}$ | European | Neale lab ^1^ |
| Previous | ukb-a-224 | case 118,419  control 217,605 | logOR | P<5x${10}^{-8}$ | European | Neale lab ^1^ |
| Never | ukb-d-20116_0 | case 195,068  control 164,638 | logOR | P<5x${10}^{-8}$ | European | Neale lab ^1^ |
| Amount of smoking | | | | | | |
| Packyears | ukb-b-10831 | 142,387 | SD | P<5x${10}^{-8}$ | European | Ben Elsworth et al [5] |
| Number of days/week of physical activity 10+ minutes | | | | | | |
| Moderate | ukb-b-4710 | 440,266 | SD | P<5x${10}^{-8}$ | European | Ben Elsworth et al [5] |
| Vigorous | ukb-b-151 | 440,512 | SD | P<5x${10}^{-8}$ | European | Ben Elsworth et al [5] |
| Types of physical activity in last 4 weeks | | | | | | |
| Heavy DIY | ukb-b-13184 | case 197,006  control 263,370 | logOR | P<5x${10}^{-8}$ | European | Ben Elsworth et al [5] |
| Light DIY | ukb-b-11495 | case 236,244  control 224,132 | logOR | P<5x${10}^{-8}$ | European | Ben Elsworth et al [5] |
| Strenuous sports | ukb-b-7663 | case 47,468  control 412,908 | logOR | P<5x${10}^{-8}$ | European | Ben Elsworth et al [5] |
| Walking for pleasure | ukb-b-7337 | case 329,755  control 130,621 | logOR | P<5x${10}^{-8}$ | European | Ben Elsworth et al [5] |
| Other physical activity | ukb-b-8764 | case 222,470  control 237,906 | logOR | P<5x${10}^{-8}$ | European | Ben Elsworth et al [5] |
| No physical activity | ukb-b-15869 | case 28,040  control 432,336 | logOR | P<5x${10}^{-8}$ | European | Ben Elsworth et al [5] |
| Food intake | | | | | | |
| Dried fruit | ukb-b-16576 | 421,764 | SD | P<5x${10}^{-8}$ | European | Ben Elsworth et al [5] |
| Fresh fruit | ukb-b-3881 | 446,462 | SD | P<5x${10}^{-8}$ | European | Ben Elsworth et al [5] |
| Salad | ukb-b-1996 | 435,435 | SD | P<5x${10}^{-8}$ | European | Ben Elsworth et al [5] |
| Cooked vegetable | ukb-b-8089 | 448,651 | SD | P<5x${10}^{-8}$ | European | Ben Elsworth et al [5] |
| Oily fish | ukb-b-2209 | 460,443 | SD | P<5x${10}^{-8}$ | European | Ben Elsworth et al [5] |
| Non oily fish | ukb-b-17627 | 460,800 | SD | P<5x${10}^{-8}$ | European | Ben Elsworth et al [5] |
| Cereal | ukb-b-15926 | 441,640 | SD | P<5x${10}^{-8}$ | European | Ben Elsworth et al [5] |
| Bacon | ukb-b-4414 | 64,949 | SD | P<5x${10}^{-8}$ | European | Ben Elsworth et al [5] |
| Processed meat | ukb-b-6324 | 461,981 | SD | P<5x${10}^{-8}$ | European | Ben Elsworth et al [5] |
| *Note:* Heavy DIY = e.g.: weeding, lawn mowing, carpentry, digging; Light DIY = e.g.: pruning, watering the lawn; Walking = Walking for pleasure (not as a means of transport); Other PA = Other physical activity, e.g.: swimming, cycling, keep fit, bowling; No PA = None of the above physical activity.  ^1^ [Rapid GWAS of thousands of phenotypes for 337,000 samples in the UK Biobank — Neale lab](https://www.nealelab.is/blog/2017/7/19/rapid-gwas-of-thousands-of-phenotypes-for-337000-samples-in-the-uk-biobank) | | | | | | |

# Table S14. EUR MR analysis result: the causal effects of the CVH factors on HannumEAA

|  | | | | IVW | | |  | Weighted Median | | |  | MR-Egger | | |  | MR-PRESSO | | | | | |
| --- | --- | --- | --- | --- | --- | --- | --- | --- | --- | --- | --- | --- | --- | --- | --- | --- | --- | --- | --- | --- | --- |
| CVH Factor | No. of SNPs | | F | β (SE), year | p Value | q Value |  | β (SE), year | p Value | q Value |  | β (SE), year | p Value | q Value |  | No. of outliers | | β (SE), years | | p Value | q Value |
| Clinical Factor | | | | | | | | | | | | | | | | | | | | | |
| Total cholesterol (1SD) | 117 | 125 | | -0.083 (0.073) | 2.51E-01 | 5.44E-01 | -0.060 (0.114) | | 5.96E-01 | 8.41E-01 | -0.112 (0.120) | | 3.53E-01 | 8.61E-01 | 2 | | -0.051 (0.065) | | 4.42E-01 | | 7.12E-01 |
| Fasting glucose (1SD) | 85 | 109 | | -0.191 (0.200) | 3.40E-01 | 6.31E-01 | 0.515 (0.319) | | 1.06E-01 | 5.60E-01 | 0.951 (0.372) | | 1.23E-02 | 3.21E-01 | 0 | | 0.191 (0.200) | | 3.42E-01 | | 7.12E-01 |
| DBP (1mmHg) | 793 | 65 | | 0.002 (0.009) | 8.58E-01 | 9.09E-01 | -0.017 (0.013) | | 1.88E-01 | 6.94E-01 | -0.020 (0.022) | | 3.59E-01 | 8.61E-01 | 3 | | 0.004 (0.008) | | 6.24E-01 | | 8.14E-01 |
| SBP (1mmHg) | 755 | 64 | | 0.001 (0.005) | 8.26E-01 | 9.09E-01 | -0.005 (0.008) | | 5.05E-01 | 8.41E-01 | -0.013 (0.013) | | 3.38E-01 | 8.61E-01 | 5 | | -0.001 (0.005) | | 8.73E-01 | | 9.09E-01 |
| Lifestyle Factor |  |  | |  |  |  |  | |  |  |  | |  |  |  | |  | |  | |  |
| BMI (1SD) | 941 | 58 | | 0.200 (0.079) | 1.12E-02 | 2.92E-01 | 0.212 (0.128) | | 9.79E-02 | 5.60E-01 | 0.462 (0.243) | | 5.77E-02 | 7.50E-03 | 2 | | 0.184 (0.078) | | 1.82E-02 | | 2.28E-01 |
| Smoking status | | | | | | | | | | | | | | | | | | | | | |
| Current | 16 | 40 | | 2.016 (1.630) | 2.16E-01 | 5.28E-01 | 3.481 (2.313) | | 1.32E-01 | 5.73E-01 | -5.379 (7.309) | | 4.74E-01 | 8.61E-01 | 0 | | 2.016 (1.517) | | 2.04E-01 | | 5.39E-01 |
| Previous | 20 | 38 | | -0.335 (1.004) | 7.38E-01 | 9.09E-01 | 0.222 (1.322) | | 8.67E-01 | 9.39E-01 | 2.271 (5.885) | | 7.04E-01 | 8.61E-01 | 0 | | -0.335 (1.004) | | 7.42E-01 | | 8.43E-01 |
| Never | 76 | 41 | | 0.205 (0.488) | 6.74E-01 | 9.09E-01 | 0.162 (0.693) | | 8.15E-01 | 9.22E-01 | 1.425 (2.161) | | 5.12E-01 | 8.61E-01 | 0 | | 0.205 (0.488) | | 6.75E-01 | | 8.14E-01 |
| Amount of smoking | | | | | | | | | | | | | | | | | | | | | |
| Packyears (1SD) | 11 | 73 | | -0.067 (0.361) | 8.53E-01 | 9.09E-01 | -0.195 (0.388) | | 6.15E-01 | 8.41E-01 | -0.464 (0.870) | | 6.70E-01 | 8.61E-01 | 0 | | -0.067 (0.361) | | 8.56E-01 | | 9.09E-01 |
| Number of days/week of physical activity 10+ minutes | | | | | | | | | | | | | | | | | | | | | |
| Moderate (1SD) | 16 | 36 | | -0.252 (0.278) | 3.65E-01 | 6.33E-01 | -0.440 (0.364) | | 2.27E-01 | 6.94E-01 | -2.292 (1.737) | | 2.08E-01 | 8.61E-01 | 0 | | -0.252 (0.278) | | 3.80E-01 | | 7.12E-01 |
| Vigorous (1SD) | 11 | 40 | | -0.494 (0.350) | 1.59E-01 | 4.86E-01 | -0.485 (0.430) | | 2.59E-01 | 6.94E-01 | -1.070 (2.798) | | 7.11E-01 | 8.61E-01 | 0 | | -0.494 (0.222) | | 5.03E-02 | | 3.14E-01 |
| Types of physical activity in last 4 weeks | | | | | | | | | | | | | | | | | | | | | |
| Heavy DIY | 19 | 35 | | 1.664 (1.170) | 1.55E-01 | 4.86E-01 | 2.522 (1.567) | | 1.08E-01 | 5.60E-01 | -3.771 (7.623) | | 6.27E-01 | 8.61E-01 | 0 | | 1.664 (1.014) | | 1.18E-01 | | 3.86E-01 |
| Light DIY | 12 | 41 | | -0.930 (1.599) | 5.61E-01 | 8.58E-01 | -1.738 (1.840) | | 3.45E-01 | 7.55E-01 | 3.759 (6.498) | | 5.76E-01 | 8.61E-01 | 0 | | -0.930 (1.599) | | 5.73E-01 | | 7.95E-01 |
| Strenuous sports | 6 | 39 | | -7.449 (5.405) | 1.68E-01 | 4.86E-01 | -5.039 (4.539) | | 2.67E-01 | 6.94E-01 | -9.138 (28.190) | | 7.62E-01 | 8.61E-01 | 1 | | -2.745 (3.169) | | 4.35E-01 | | 7.12E-01 |
| Walking | 21 | 34 | | -1.196 (1.215) | 3.25E-01 | 6.31E-01 | -1.596 (1.703) | | 3.49E-01 | 7.55E-01 | -7.121 (12.611) | | 5.79E-01 | 8.61E-01 | 0 | | -1.196 (0.982) | | 2.37E-01 | | 5.39E-01 |
| Other physical activity | 14 | 38 | | -1.959 (1.239) | 1.14E-01 | 4.86E-01 | -3.043 (1.733) | | 7.91E-02 | 5.60E-01 | -17.217 (9.376) | | 9.12E-02 | 7.90E-01 | 0 | | -1.959 (1.155) | | 1.14E-01 | | 3.86E-01 |
| No physical activity | 5 | 33 | | -18.028 (11.960) | 1.32E-01 | 4.86E-01 | -13.894 (7.124) | | 5.11E-02 | 5.60E-01 | -144.64 (149.341) | | 4.99E-01 | 8.61E-01 | 2 | | **-14.010 (0.154)** | | **1.21E-04** | | **3.04E-03** |
| Food intake | | | | | | | | | | | | | | | | | | | | | |
| Dried fruit (1SD) | 43 | 42 | | -0.595 (0.489) | 2.33E-01 | 5.28E-01 | -0.068 (0.620) | | 9.13E-01 | 9.49E-01 | 0.002 (2.239) | | 9.99E-01 | 9.99E-01 | 0 | | -0.595 (0.489) | | 2.30E-01 | | 5.39E-01 |
| Fresh fruit (1SD) | 56 | 45 | | 0.797 (0.487) | 1.02E-01 | 4.86E-01 | 0.579 (0.748) | | 4.39E-01 | 8.41E-01 | 0.936 (1.814) | | 6.08E-01 | 8.61E-01 | 0 | | 0.797 (0.468) | | 9.39E-02 | | 3.86E-01 |
| Salad (1SD) | 17 | 37 | | -0.199 (1.252) | 8.74E-01 | 9.09E-01 | 0.830 (1.292) | | 5.21E-01 | 8.41E-01 | -1.079 (6.496) | | 8.70E-01 | 9.25E-01 | 1 | | 0.750 (0.980) | | 4.56E-01 | | 7.12E-01 |
| Cooked vegetable (1SD) | 17 | 38 | | 0.561 (0.860) | 5.14E-01 | 8.36E-01 | 0.331 (1.099) | | 7.63E-01 | 9.22E-01 | 3.145 (9.667) | | 7.49E-01 | 8.61E-01 | 0 | | 0.561 (0.860) | | 5.24E-01 | | 7.70E-01 |
| Oily fish (1SD) | 69 | 44 | | -0.454 (0.291) | 1.19E-01 | 4.86E-01 | -0.017 (0.425) | | 9.67E-01 | 9.67E-01 | -1.037 (1.269) | | 4.17E-01 | 8.61E-01 | 0 | | -0.454(0.291) | | 1.24E-01 | | 3.86E-01 |
| Non oily fish (1SD) | 12 | 44 | | -0.073 (0.919) | 9.37E-01 | 9.37E-01 | 0.306 (1.147) | | 7.90E-01 | 9.22E-01 | -2.040 (4.687) | | 6.73E-01 | 8.61E-01 | 0 | | 0.073 (0.919) | | 9.38E-01 | | 9.38E-01 |
| Cereal (1SD) | 39 | 45 | | -0.840 (0.404) | 3.78E-02 | 4.86E-01 | -0.308 (0.595) | | 6.04E-01 | 8.41E-01 | -0.755 (1.742) | | 6.64E-01 | 8.61E-01 | 0 | | -0.840 (0.402) | | 4.34E-02 | | 3.14E-01 |
| Bacon (1SD) | 3 | 32 | | 0.261 (0.805) | 7.46E-01 | 9.09E-01 | 0.304 (0.934) | | 7.45E-01 | 9.22E-01 | -2.929 (1.663) | | 8.89E-01 | 9.25E-01 | - | | - | | - | | - |
| Processed meat (1SD) | 23 | 39 | | 0.196 (0.485) | 6.86E-01 | 9.09E-01 | 0.355 (0.690) | | 6.07E-01 | 8.41E-01 | -0.755 (2.460) | | 7.56E-01 | 8.61E-01 | 0 | | 0.196 (0.475) | | 6.84E-01 | | 8.14E-01 |
| *Note:* DBP: diastolic blood pressure; SBP: systolic blood pressure; BMI: body mass index; Moderate: number of days/week of moderate physical activity 10+ minutes; Vigorous: Number of days/week of vigorous physical activity 10+ minutes; Heavy DIY: e.g., weeding, lawn mowing, carpentry, digging; Light DIY: e.g., pruning, watering the lawn; Walking: walking for pleasure (not as a means of transport); Bold font indicates that the MR results are significant after FDR correction (q<0.05). | | | | | | | | | | | | | | | | | | | | | |

# Table S15. EUR MR analysis result: the causal effects of the CVH factors on IEAA

|  | | | IVW | | |  | Weighted Median | | | | | |  | MR-Egger | | | | | |  | MR-PRESSO | | | | | |
| --- | --- | --- | --- | --- | --- | --- | --- | --- | --- | --- | --- | --- | --- | --- | --- | --- | --- | --- | --- | --- | --- | --- | --- | --- | --- | --- |
| CVH Factor | No. of SNPs | F | β (SE), year | p Value | q Value |  | β (SE), year | | p Value | | q Value | |  | β (SE), year | | p Value | | q Value | |  | No. of outliers | | β (SE), years | | p Value | q Value |
| Clinical factor | | | | | | | | | | | | | | | | | | | | | | | | | | |
| Total cholesterol (1SD) | 117 | 125 | -0.036 (0.066) | 5.85E-01 | 9.50E-01 | 0.005 (0.121) | | 9.69E-01 | | 9.89E-01 | | 0.020 (0.108) | | | 8.53E-01 | | 9.55E-01 | | 0 | | | -0.036 (0.061) | | 5.61E-01 | | 9.36E-01 |
| Fasting glucose (1SD) | 85 | 109 | 0.026 (0.191) | 8.91E-01 | 9.58E-01 | -0.020 (0.291) | | 9.46E-01 | | 9.89E-01 | | 0.759 (0.360) | | | 3.83E-02 | | 5.38E-01 | | 0 | | | 0.026 (0.191) | | 8.91E-01 | | 9.58E-01 |
| DBP (1mmHg) | 793 | 65 | 0.008 (0.009) | 3.68E-01 | 8.25E-01 | 0.002 (0.012) | | 8.73E-01 | | 9.89E-01 | | -0.023 (0.023) | | | 3.20E-01 | | 8.33E-01 | | 7 | | | 0.012 (0.009) | | 1.65E-01 | | 9.36E-01 |
| SBP (1mmHg) | 756 | 64 | 0.004 (0.005) | 4.15E-01 | 8.25E-01 | -0.005 (0.008) | | 4.98E-01 | | 9.89E-01 | | 0.001 (0.014) | | | 9.17E-01 | | 9.55E-01 | | 5 | | | 0.003 (0.005) | | 5.49E-01 | | 9.36E-01 |
| Lifestyle factor |  |  |  |  |  |  | |  | |  | |  | | |  | |  | |  | | |  | |  | |  |
| BMI (1SD) | 941 | 58 | **0.258 (0.083)** | **1.83E-03** | **4.77E-02** | 0.144 (0.127) | | 2.58E-01 | | 9.89E-01 | | 0.270 (0.256) | | | 2.29E-01 | | 8.33E-01 | | 3 | | | 0.250 (0.082) | | 2.29E-03 | | 5.74E-02 |
| Smoking status | | | | | | | | | | | | | | | | | | | | | | | | | | |
| Current | 16 | 40 | 0.896 (1.877) | 6.33E-01 | 9.58E-01 | -0.542 (2.189) | | 8.04E-01 | | 9.89E-01 | | -3.762 (8.678) | | | 6.71E-01 | | 9.55E-01 | | 0 | | | 0.896 (1.887) | | 6.40E-01 | | 9.58E-01 |
| Previous | 20 | 38 | -1.443 (1.013) | 1.54E-01 | 8.25E-01 | -1.853 (1.371) | | 1.76E-01 | | 9.89E-01 | | 2.514 (5.924) | | | 6.76E-01 | | 9.55E-01 | | 0 | | | -1.443 (1.013) | | 1.71E-01 | | 9.36E-01 |
| Never | 76 | 41 | 0.773 (0.507) | 1.48E-01 | 8.25E-01 | 0.890 (0.736) | | 2.26E-01 | | 9.89E-01 | | 1.255 (2.254) | | | 5.79E-01 | | 9.55E-01 | | 0 | | | 0.733 (0.507) | | 1.52E-01 | | 9.36E-01 |
| Amount of smoking | | | | | | | | | | | | | | | | | | | | | | | | | | |
| Packyears (1SD) | 11 | 73 | 0.467 (0.436) | 2.84E-01 | 8.25E-01 | 0.259 (0.437) | | 5.53E-01 | | 9.89E-01 | | 0.370 (1.065) | | | 7.36E-01 | | 9.55E-01 | | 0 | | | 0.467 (0.436) | | 3.09E-01 | | 9.36E-01 |
| Number of days/week of physical activity 10+ minutes | | | | | | | | | | | | | | | | | | | | | | | | | | |
| Moderate (1SD) | 16 | 36 | 0.113 (0.319) | 7.23E-01 | 9.58E-01 | 0.061 (0.361) | | 8.66E-01 | | 9.89E-01 | | 1.244 (2.070) | | | 5.57E-01 | | 9.55E-01 | | 0 | | | 0.113 (0.319) | | 7.28E-01 | | 9.58E-01 |
| Vigorous (1SD) | 11 | 40 | -0.502 (0.458) | 2.73E-01 | 8.25E-01 | -0.526 (0.527) | | 3.18E-01 | | 9.89E-01 | | 2.438 (3.753) | | | 5.32E-01 | | 9.55E-01 | | 0 | | | -0.502 (0.458) | | 2.99E-01 | | 9.36E-01 |
| Types of physical activity in last 4 weeks | | | | | | | | | | | | | | | | | | | | | | | | | | |
| Heavy DIY | 19 | 35 | 0.113 (1.342) | 9.33E-01 | 9.58E-01 | -0.113 (1.749) | | 9.49E-01 | | 9.89E-01 | | 2.547 (8.981) | | | 7.80E-01 | | 9.55E-01 | | 0 | | | 0.113 (1.342) | | 9.34E-01 | | 9.58E-01 |
| Light DIY | 13 | 40 | 0.179 (1.349) | 8.95E-01 | 9.58E-01 | -0.434 (1.808) | | 8.10E-01 | | 9.89E-01 | | -3.401 (5.482) | | | 5.48E-01 | | 9.55E-01 | | 0 | | | 0.179 (1.254) | | 8.89E-01 | | 9.58E-01 |
| Strenuous sports | 6 | 39 | 2.469 (3.706) | 5.05E-01 | 8.76E-01 | 3.738 (3.960) | | 3.45E-01 | | 9.89E-01 | | 7.074 (19.262) | | | 7.32E-01 | | 9.55E-01 | | 0 | | | 2.469 (3.706) | | 5.35E-01 | | 9.36E-01 |
| Walking | 21 | 34 | -1.202 (1.252) | 3.37E-01 | 8.25E-01 | -0.808 (1.746) | | 6.37E-01 | | 9.89E-01 | | -18.275 (13.021) | | | 1.77E-01 | | 7.65E-01 | | 0 | | | -1.202 (1.220) | | 3.36E-01 | | 9.36E-01 |
| Other physical activity | 14 | 38 | -1.058 (1.275) | 4.07E-01 | 8.25E-01 | -1.716 (1.784) | | 3.36E-01 | | 9.89E-01 | | -2.005 (9.629) | | | 8.39E-01 | | 9.55E-01 | | 0 | | | -1.058 (1.183) | | 3.87E-01 | | 9.36E-01 |
| No physical activity | 5 | 33 | -0.937 (4.966) | 8.05E-01 | 9.58E-01 | 0.645 (6.163) | | 9.17E-01 | | 9.89E-01 | | -75.506 (55.251) | | | 2.65E-01 | | 8.33E-01 | | 0 | | | -0.937 (4.966) | | 8.59E-01 | | 9.58E-01 |
| Food intake | | | | | | | | | | | | | | | | | | | | | | | | | | |
| Dried fruit (1SD) | 43 | 42 | -0.349 (0.433) | 4.31E-01 | 8.25E-01 | -0.726 (0.623) | | 2.44E-01 | | 9.89E-01 | | -3.288 (1.975) | | | 1.04E-01 | | 5.38E-01 | | 0 | | | -0.349 (0.443) | | 4.35E-01 | | 9.36E-01 |
| Fresh fruit (1SD) | 56 | 45 | 0.453 (0.552) | 4.12E-01 | 8.25E-01 | 0.191 (0.797) | | 8.11E-01 | | 9.89E-01 | | 2.509 (2.053) | | | 2.27E-01 | | 8.33E-01 | | 0 | | | 0.453 (0.552) | | 4.16E-01 | | 9.36E-01 |
| Salad (1SD) | 17 | 37 | -0.233 (0.892) | 8.02E-01 | 9.58E-01 | -0.268 (1.211) | | 8.25E-01 | | 9.89E-01 | | -0.001 (4.642) | | | 9.99E-01 | | 9.99E-01 | | 0 | | | -0.223 (0.892) | | 8.06E-01 | | 9.58E-01 |
| Cooked vegetable (1SD) | 17 | 38 | 0.146 (0.792) | 8.54E-01 | 9.58E-01 | 0.791 (1.134) | | 4.86E-01 | | 9.89E-01 | | -17.119 (8.516) | | | 6.28E-02 | | 5.38E-01 | | 0 | | | 0.146 (0.792) | | 8.56E-01 | | 9.58E-01 |
| Oily fish (1SD) | 69 | 44 | -0.087 (0.338) | 7.97E-01 | 9.58E-01 | 0.006 (0.442) | | 9.89E-01 | | 9.89E-01 | | -2.842 (1.436) | | | 5.19E-02 | | 5.38E-01 | | 0 | | | -0.087 (0.338) | | 7.98E-01 | | 9.58E-01 |
| Non oily fish (1SD) | 12 | 44 | -0.760 (0.993) | 4.44E-01 | 8.25E-01 | -0.959 (1.171) | | 4.13E-01 | | 9.89E-01 | | -0.538 (5.111) | | | 9.18E-01 | | 9.55E-01 | | 0 | | | -0.760 (0.993) | | 4.60E-01 | | 9.36E-01 |
| Cereal (1SD) | 39 | 45 | 0.457 (0.428) | 2.86E-01 | 8.25E-01 | -0.144 (0.620) | | 8.17E-01 | | 9.89E-01 | | -0.936 (1.822) | | | 6.10E-01 | | 9.55E-01 | | 0 | | | 0.457 (0.428) | | 2.93E-01 | | 9.36E-01 |
| Bacon (1SD) | 3 | 32 | -1.783 (0.835) | 3.27E-02 | 4.26E-01 | -1.550 (1.043) | | 1.38E-01 | | 9.89E-01 | | -0.524 (1.729) | | | 8.13E-01 | | 9.55E-01 | | - | | | - | | - | | - |
| Processed meat (1SD) | 23 | 39 | -0.032 (0.596) | 9.58E-01 | 9.58E-01 | -0.486 (0.700) | | 4.87E-01 | | 9.89E-01 | | 5.010 (2.895) | | | 9.82E-02 | | 5.38E-01 | | 0 | | | -0.032 (0.596) | | 9.58E-01 | | 9.58E-01 |
| *Note:* DBP: diastolic blood pressure; SBP: systolic blood pressure; BMI: body mass index; Moderate: number of days/week of moderate physical activity 10+ minutes; Vigorous: Number of days/week of vigorous physical activity 10+ minutes; Heavy DIY: e.g., weeding, lawn mowing, carpentry, digging; Light DIY: e.g., pruning, watering the lawn; Walking: walking for pleasure (not as a means of transport); Bold font indicates that the MR results are significant after FDR correction (q<0.05). | | | | | | | | | | | | | | | | | | | | | | | | | | |

# Table S16. EUR MR analysis result: the causal effects of the CVH factors on PhenoEAA

|  | | | | IVW | | |  | Weighted Median | | |  | MR-Egger | | |  | MR-PRESSO | | | | | |
| --- | --- | --- | --- | --- | --- | --- | --- | --- | --- | --- | --- | --- | --- | --- | --- | --- | --- | --- | --- | --- | --- |
| CVH Factor | No. of SNPs | | F | β (SE), year | p Value | q Value |  | β (SE), year | p Value | q Value |  | β (SE), year | p Value | q Value |  | No. of outliers | | β (SE), years | | p Value | q Value |
| Clinical Factor | | | | | | | | | | | | | | | | | | | | | |
| Total cholesterol (1SD) | 117 | 125 | | -0.038 (0.092) | 6.81E-01 | 7.76E-01 | -0.013 (0.135) | | 9.25E-01 | 9.56E-01 | -0.010 (0.152) | | 9.74E-01 | 9.85E-01 | 2 | | -0.034 (0.087) | | 6.92E-01 | | 7.86E-01 |
| Fasting glucose (1SD) | 85 | 109 | | -0.224 (0.235) | 3.41E-01 | 6.69E-01 | 0.245 (0.380) | | 5.19E-01 | 9.16E-01 | 1.002 (0.451) | | 2.90E-02 | 6.61E-01 | 0 | | -0.224 (0.234) | | 3.40E-01 | | 6.54E-01 |
| DBP (1mmHg) | 793 | 65 | | **0.035 (0.010)** | **6.77E-04** | **8.80E-03** | 0.030 (0.016) | | 6.17E-02 | 4.69E-01 | 0.039 (0.027) | | 1.45E-01 | 6.61E-01 | 2 | | **0.036 (0.010)** | | **4.45E-04** | | **5.56E-03** |
| SBP (1mmHg) | 756 | 64 | | 0.014 (0.006) | 2.65E-02 | 1.38E-01 | 0.010 (0.010) | | 2.83E-01 | 7.36E-01 | 0.025 (0.017) | | 1.45E-01 | 6.61E-01 | 3 | | 0.015 (0.006) | | 1.90E-02 | | 1.03E-01 |
| Lifestyle Factor |  |  | |  |  |  |  | |  |  |  | |  |  |  | |  | |  | |  |
| BMI (1SD) | 941 | 58 | | **0.604 (0.104)** | **5.86E-09** | **1.52E-07** | **0.745 (0.157)** | | **2.00E-06** | **5.20E-05** | 0.616 (0.321) | | 5.54E-02 | 6.61E-01 | 3 | | **0.617 (0.102)** | | **2.02E-09** | | **5.05E-08** |
| Smoking status | | | | | | | | | | | | | | | | | | | | | |
| Current | 16 | 40 | | 0.964 (2.097) | 6.46E-01 | 7.76E-01 | 1.754 (2.937) | | 5.50E-01 | 9.16E-01 | -6.623 (9.495) | | 4.97E-01 | 8.05E-01 | 0 | | 0.964 (2.097) | | 6.52E-01 | | 7.86E-01 |
| Previous | 20 | 38 | | -0.190 (1.571) | 9.04E-01 | 9.73E-01 | -0.126 (1.889) | | 9.47E-01 | 9.56E-01 | 6.692 (9.091) | | 4.71E-01 | 8.05E-01 | 0 | | -0.190 (1.571) | | 9.05E-01 | | 9.43E-01 |
| Never | 76 | 41 | | -1.089 (0.766) | 1.55E-01 | 4.46E-01 | -0.768 (0.906) | | 3.97E-01 | 9.16E-01 | 2.125 (3.389) | | 5.33E-01 | 8.05E-01 | 2 | | -1.227 (0.690) | | 7.96E-02 | | 2.84E-01 |
| Amount of smoking | | | | | | | | | | | | | | | | | | | | | |
| Packyears (1SD) | 11 | 73 | | 0.596 (0.396) | 1.32E-01 | 4.46E-01 | 0.304 (0.511) | | 5.52E-01 | 9.16E-01 | 0.217 (0.916) | | 8.18E-01 | 9.51E-01 | 0 | | 0.596 (0.378) | | 1.46E-01 | | 4.40E-01 |
| Number of days/week of physical activity 10+ minutes | | | | | | | | | | | | | | | | | | | | | |
| Moderate (1SD) | 16 | 36 | | 0.132 (0.326) | 6.87E-01 | 7.76E-01 | -0.117 (0.411) | | 7.75E-01 | 9.16E-01 | 0.325 (2.067) | | 8.77E-01 | 9.51E-01 | 0 | | 0.132 (0.228) | | 5.72E-01 | | 7.86E-01 |
| Vigorous (1SD) | 11 | 40 | | -0.451 (0.644) | 4.84E-01 | 6.99E-01 | -0.212 (0.607) | | 7.27E-01 | 9.16E-01 | -0.884 (5.440) | | 8.74E-01 | 9.51E-01 | 0 | | -0.113 (0.567) | | 8.46E-01 | | 9.20E-01 |
| Types of physical activity in last 4 weeks | | | | | | | | | | | | | | | | | | | | | |
| Heavy DIY | 19 | 35 | | 1.285 (1.503) | 3.39E-01 | 6.99E-01 | 0.665 (2.114) | | 7.53E-01 | 9.16E-01 | 13.948 (9.837) | | 1.74E-01 | 6.61E-01 | 2 | | 1.285 (1.501) | | 4.03E-01 | | 6.62E-01 |
| Light DIY | 13 | 40 | | -2.283 (1.735) | 1.88E-01 | 4.46E-01 | -0.597 (2.488) | | 8.11E-01 | 9.16E-01 | 2.955 (7.260) | | 6.92E-01 | 9.47E-01 | 0 | | -2.283 (1.735) | | 2.13E-01 | | 5.32E-01 |
| Strenuous sports | 6 | 39 | | 0.619 (7.681) | 9.36E-01 | 9.73E-01 | 2.726 (5.795) | | 6.38E-01 | 9.16E-01 | 56.448 (28.439) | | 1.18E-01 | 6.61E-01 | 0 | | 1.850 (3.510) | | 6.35E-01 | | 7.86E-01 |
| Walking | 21 | 34 | | -2.222 (1.559) | 1.54E-01 | 4.46E-01 | -3.603 (2.199) | | 1.01E-01 | 4.69E-01 | 13.664 (16.227) | | 4.10E-01 | 8.05E-01 | 0 | | -2.222 (1.516) | | 1.58E-01 | | 4.40E-01 |
| Other physical activity | 14 | 38 | | -0.766 (1.716) | 6.55E-01 | 7.76E-01 | 1.052 (2.250) | | 6.40E-01 | 9.16E-01 | -9.080 (13.276) | | 5.07E-01 | 8.05E-01 | 0 | | -0.766 (1.716) | | 6.63E-01 | | 7.86E-01 |
| No physical activity | 5 | 33 | | 8.220 (6.255) | 1.89E-01 | 4.46E-01 | 5.694 (8.203) | | 4.88E-01 | 9.16E-01 | -64.740 (71.843) | | 4.34E-01 | 8.05E-01 | 0 | | 8.220 (6.255) | | 2.59E-01 | | 5.40E-01 |
| Food intake | | | | | | | | | | | | | | | | | | | | | |
| Dried fruit (1SD) | 43 | 42 | | -1.636 (0.596) | 6.10E-03 | 5.28E-02 | -0.923 (0.084) | | 2.51E-01 | 7.24E-01 | -2.388 (2.725) | | 3.86E-01 | 8.05E-01 | 0 | | -1.636 (0.596) | | 8.92E-03 | | 7.43E-02 |
| Fresh fruit (1SD) | 56 | 45 | | 0.526 (0.625) | 4.00E-01 | 6.69E-01 | 1.209 (0.910) | | 1.84E-01 | 6.66E-01 | 1.459 (2.321) | | 5.32E-01 | 8.05E-01 | 0 | | 0.526 (0.601) | | 3.85E-01 | | 6.62E-01 |
| Salad (1SD) | 17 | 37 | | -0.911 (1.110) | 4.12E-01 | 6.68E-01 | -0.479 (1.517) | | 7.52E-01 | 9.16E-01 | -3.498 (5.832) | | 5.58E-01 | 8.05E-01 | 0 | | -0.911 (1.110) | | 4.24E-01 | | 6.62E-01 |
| Cooked vegetable (1SD) | 17 | 38 | | 1.477 (1.211) | 2.22E-01 | 4.82E-01 | 0.078 (1.419) | | 9.56E-01 | 9.56E-01 | -4.784 (13.539) | | 7.29E-01 | 9.47E-01 | 0 | | 1.477 (1.211) | | 2.40E-01 | | 5.40E-01 |
| Oily fish (1SD) | 69 | 44 | | -0.946 (0.399) | 1.77E-02 | 1.15E-01 | -0.964 (0.548) | | 7.85E-02 | 4.69E-01 | -2.367 (1.739) | | 1.78E-01 | 6.61E-01 | 0 | | -0.946 (0.399) | | 2.05E-02 | | 1.03E-01 |
| Non oily fish (1SD) | 12 | 44 | | 1.656 (1.038) | 1.10E-01 | 4.46E-01 | 2.309 (1.365) | | 9.06E-02 | 4.69E-01 | -4.311 (5.102) | | 4.18E-01 | 8.05E-01 | 0 | | 1.656 (0.842) | | 7.48E-02 | | 2.84E-01 |
| Cereal (1SD) | 39 | 45 | | 0.014 (0.518) | 9.78E-01 | 9.78E-01 | -0.242 (0.717) | | 7.35E-01 | 9.16E-01 | -1.480 (2.194) | | 5.04E-01 | 8.05E-01 | 0 | | 0.014 (0.418) | | 9.73E-01 | | 9.73E-01 |
| Bacon (1SD) | 3 | 32 | | -1.025 (1.645) | 5.33E-01 | 7.30E-01 | -2.222 (1.383) | | 1.08E-01 | 4.69E-01 | 1.068 (4.335) | | 8.46E-01 | 9.51E-01 | - | | - | | - | | - |
| Processed meat (1SD) | 23 | 39 | | -0.525 (0.691) | 4.47E-01 | 6.84E-01 | 1.177 (0.928) | | 2.05E-01 | 6.66E-01 | 0.027 (3.653) | | 9.94E-01 | 9.41E-01 | 0 | | -0.525 (0.691) | | 4.55E-01 | | 6.69E-01 |
| *Note:* DBP: diastolic blood pressure; SBP: systolic blood pressure; BMI: body mass index; Moderate: number of days/week of moderate physical activity 10+ minutes; Vigorous: Number of days/week of vigorous physical activity 10+ minutes; Heavy DIY: e.g., weeding, lawn mowing, carpentry, digging; Light DIY: e.g., pruning, watering the lawn; Walking: walking for pleasure (not as a means of transport); Bold font indicates that the MR results are significant after FDR correction (q<0.05). | | | | | | | | | | | | | | | | | | | | | |

# Table S17. EUR MR analysis result: the causal effects of the CVH factors on DNAmPAI-1

|  | | | IVW | | | | | | |  | | Weighted Median | | | | | |  | | MR-Egger | | | | | |  | | | | MR-PRESSO | | | | | |  |
| --- | --- | --- | --- | --- | --- | --- | --- | --- | --- | --- | --- | --- | --- | --- | --- | --- | --- | --- | --- | --- | --- | --- | --- | --- | --- | --- | --- | --- | --- | --- | --- | --- | --- | --- | --- | --- |
| CVH Factor | No. of SNPs | | | F | β (SE), year | | p Value | | q Value | |  | | β (SE), year | | p Value | | q Value | |  | | β (SE), year | | p Value | | q Value | | No. of outliers | | β (SE), years | | | p Value |  | | q Value | |
| Clinical Factor | | | | | | | | | | | | | | | | | | | | | | | | | | | | | | | | | | | |  |
| Total cholesterol (1SD) | 117 | 125 | -0.126 (0.159) | | | 4.28E-01 | | 8.59E-01 | | -0.074 (0.271) | | | | 7.86E-01 | | 9.70E-01 | | 0.092 (0.261) | | | | 7.26E-01 | | 7.83E-01 | | 0 | | -0.126 (0.140) | | | 3.85E-01 | | | 7.62E-01 | |  |
| Fasting glucose (1SD) | 83 | 111 | -0.068 (0.469) | | | 8.85E-01 | | 9.20E-01 | | 0.691 (0.735) | | | | 3.47E-01 | | 9.70E-01 | | 1.565 (0.913) | | | | 9.05E-02 | | 7.28E-01 | | 0 | | -0.068 (0.440) | | | 8.79E-01 | | | 9.16E-01 | |  |
| DBP (1mmHg) | 791 | 65 | 0.022 (0.019) | | | 2.41E-01 | | 8.59E-01 | | 0.011 (0.032) | | | | 7.39E-01 | | 9.70E-01 | | -0.018 (0.051) | | | | 7.29E-01 | | 7.83E-01 | | 0 | | 0.022 (0.019) | | | 2.37E-01 | | | 7.62E-01 | |  |
| SBP (1mmHg) | 753 | 64 | 0.009 (0.012) | | | 4.17E-01 | | 8.59E-01 | | 0.005 (0.018) | | | | 7.96E-01 | | 9.70E-01 | | -0.011 (0.032) | | | | 7.25E-01 | | 7.83E-01 | | 0 | | 0.009 (0.011) | | | 4.17E-01 | | | 7.62E-01 | |  |
| Lifestyle Factor |  |  |  | | |  | |  | |  | | | |  | |  | |  | | | |  | |  | |  | |  | | |  | | |  | |  |
| BMI (1SD) | 937 | 58 | 0.566 (0.192) | | | 3.21E-03 | | 8.34E-02 | | 0.367 (0.309) | | | | 2.35E-01 | | 9.70E-01 | | 0.940 (0.599) | | | | 1.17E-01 | | 7.28E-01 | | 0 | | 0.566 (0.187) | | | 3.29E-03 | | | 8.22E-01 | |  |
| Smoking status | | | | | | | | | | | | | | | | | | | | | | | | | | | | | | | | | | | |  |
| Current | 16 | 40 | 1.805 (3.987) | | | 6.51E-01 | | 8.59E-01 | | 0.620 (5.663) | | | | 9.13E-01 | | 9.70E-01 | | 25.371 (17.470) | | | | 1.68E-01 | | 7.28E-01 | | 0 | | 1.805 (3.980) | | | 6.57E-01 | | | 7.82E-01 | |  |
| Previous | 20 | 38 | -2.384 (2.372) | | | 3.15E-01 | | 8.59E-01 | | -0.704 (3.173) | | | | 8.24E-01 | | 9.70E-01 | | 19.724 (13.017) | | | | 1.47E-01 | | 7.28E-01 | | 0 | | -2.684 (2.243) | | | 3.28E-01 | | | 7.62E-01 | |  |
| Never | 75 | 41 | -0.778 (1.284) | | | 5.45E-01 | | 8.59E-01 | | -1.203 (1.816) | | | | 5.08E-01 | | 9.70E-01 | | 4.465 (5.753) | | | | 4.40E-01 | | 7.83E-01 | | 0 | | -0.778 (1.240) | | | 5.46E-01 | | | 7.62E-01 | |  |
| Amount of smoking | | | | | | | | | | | | | | | | | | | | | | | | | | | | | | | | | | | |  |
| Packyears (1SD) | 11 | 73 | 0.569 (0.756) | | | 4.52E-01 | | 8.59E-01 | | -0.279 (1.006) | | | | 7.82E-01 | | 9.70E-01 | | 0.133 (1.736) | | | | 9.41E-01 | | 9.41E-01 | | 0 | | 0.569 (0.672) | | | 4.32E-01 | | | 7.62E-01 | |  |
| Number of days/week of physical activity 10+ minutes | | | | | | | | | | | | | | | | | | | | | | | | | | | | | | | | | | | |  |
| Moderate (1SD) | 16 | 36 | -0.099 (0.624) | | | 8.74E-01 | | 9.20E-01 | | 0.081 (0.792) | | | | 9.19E-01 | | 9.70E-01 | | -1.262 (3.933) | | | | 7.53E-01 | | 7.83E-01 | | 0 | | -0.099 (0.550) | | | 8.60E-01 | | | 9.16E-01 | |  |
| Vigorous (1SD) | 11 | 40 | 0.791 (0.863) | | | 3.59E-01 | | 8.59E-01 | | 0.653 (1.141) | | | | 5.67E-01 | | 9.70E-01 | | -4.534 (6.752) | | | | 5.19E-01 | | 7.83E-01 | | 0 | | 0.791 (0.734) | | | 3.06E-01 | | | 7.62E-01 | |  |
| Types of physical activity in last 4 weeks | | | | | | | | | | | | | | | | | | | | | | | | | | | | | | | | | | | |  |
| Heavy DIY | 19 | 35 | 1.599 (2.924) | | | 5.84E-01 | | 8.59E-01 | | 2.528 (3.826) | | | | 5.09E-01 | | 9.70E-01 | | 26.939 (19.163) | | | | 1.78E-01 | | 7.28E-01 | | 0 | | 1.599 (2.205) | | | 4.78E-01 | | | 7.62E-01 | |  |
| Light DIY | 13 | 40 | -1.894 (4.807) | | | 6.94E-01 | | 8.59E-01 | | -3.211 (4.555) | | | | 4.81E-01 | | 9.70E-01 | | -13.300 (22.486) | | | | 5.66E-01 | | 7.83E-01 | | 2 | | -1.847 (3.180) | | | 5.74E-01 | | | 7.62E-01 | |  |
| Strenuous sports | 6 | 39 | 5.162 (8.105) | | | 5.24E-01 | | 8.59E-01 | | 2.686 (10.011) | | | | 7.88E-01 | | 9.70E-01 | | 32.040 (40.159) | | | | 4.70E-01 | | 7.83E-01 | | 0 | | 5.162 (5.418) | | | 3.84E-01 | | | 7.62E-01 | |  |
| Walking | 21 | 34 | -0.590 (3.240) | | | 8.55E-01 | | 9.20E-01 | | -1.901 (4.311) | | | | 6.59E-01 | | 9.70E-01 | | -15.067 (34.654) | | | | 6.69E-01 | | 7.83E-01 | | 0 | | -0.590 (3.240) | | | 8.57E-01 | | | 9.16E-01 | |  |
| Other physical activity | 14 | 38 | -5.706 (3.029) | | | 5.96E-02 | | 4.87E-01 | | -6.677 (4.041) | | | | 9.85E-02 | | 9.70E-01 | | 8.952 (22.536) | | | | 6.98E-01 | | 7.83E-01 | | 0 | | -5.706 (1.899) | | | 1.48E-02 | | | 1.85E-01 | |  |
| No physical activity | 5 | 33 | -4.694 (11.453) | | | 6.82E-01 | | 8.59E-01 | | -0.992 (14.351) | | | | 9.45E-01 | | 9.70E-01 | | 61.318 (131.547) | | | | 6.73E-01 | | 7.83E-01 | | 0 | | -4.694 (7.783) | | | 5.79E-01 | | | 7.62E-01 | |  |
| Food intake | | | | | | | | | | | | | | | | | | | | | | | | | | | | | | | | | | | |  |
| Dried fruit (1SD) | 43 | 42 | -2.320 (1.080) | | | 3.16E-02 | | 4.11E-01 | | -2.326 (1.463) | | | | 1.12E-01 | | 9.70E-01 | | -3.966 (4.842) | | | | 4.18E-01 | | 7.83E-01 | | 0 | | -2.320 (0.596) | | | 3.74E-02 | | | 3.12E-01 | |  |
| Fresh fruit (1SD) | 56 | 45 | 0.736 (1.198) | | | 5.39E-01 | | 8.59E-01 | | 0.344 (1.789) | | | | 8.48E-01 | | 9.70E-01 | | 1.926 (4.417) | | | | 6.65E-01 | | 7.83E-01 | | 0 | | 0.736 (0.601) | | | 5.38E-01 | | | 7.62E-01 | |  |
| Salad (1SD) | 17 | 37 | -1.821 (2.206) | | | 4.09E-01 | | 8.59E-01 | | -0.117 (3.087) | | | | 9.70E-01 | | 9.70E-01 | | -14.302 (13.193) | | | | 2.95E-01 | | 7.68E-01 | | 0 | | -1.821 (1.110) | | | 3.99E-01 | | | 7.62E-01 | |  |
| Cooked vegetable (1SD) | 17 | 38 | 1.838 (1.885) | | | 3.29E-01 | | 8.59E-01 | | 2.212 (2.654) | | | | 4.05E-01 | | 9.70E-01 | | 26.004 (20.504) | | | | 2.24E-01 | | 7.28E-01 | | 0 | | 1.838 (1.211) | | | 2.90E-01 | | | 7.62E-01 | |  |
| Oily fish (1SD) | 69 | 44 | 0.054 (0.728) | | | 9.41E-01 | | 9.41E-01 | | 0.536 (1.027) | | | | 6.01E-02 | | 9.70E-01 | | 4.060 (3.260) | | | | 2.17E-01 | | 7.28E-01 | | 0 | | 0.054 (0.399) | | | 9.41E-01 | | | 9.41E-01 | |  |
| Non oily fish (1SD) | 12 | 44 | 3.052 (2.010) | | | 1.29E-01 | | 6.70E-01 | | 2.845 (2.746) | | | | 3.00E-02 | | 9.70E-01 | | 4.223 (9.955) | | | | 6.80E-01 | | 7.83E-01 | | 0 | | 3.052 (0.842) | | | 6.77E-02 | | | 4.15E-01 | |  |
| Cereal (1SD) | 39 | 45 | 1.896 (1.065) | | | 7.50E-02 | | 4.87E-01 | | 1.808 (1.399) | | | | 1.96E-01 | | 9.70E-01 | | -8.921 (4.156) | | | | 3.85E-02 | | 7.28E-01 | | 0 | | 1.896 (0.418) | | | 8.30E-02 | | | 4.15E-01 | |  |
| Bacon (1SD) | 3 | 32 | 1.372 (4.944) | | | 7.81E-01 | | 9.20E-01 | | 1.382 (3.282) | | | | 6.74E-01 | | 9.70E-01 | | -2223.237 (996.878) | | | | 2.68E-01 | | 7.68E-01 | | - | | - | | | - | | | - | |  |
| Processed meat (1SD) | 23 | 39 | 0.559 (1.220) | | | 6.47E-01 | | 8.59E-01 | | 1.596 (1.744) | | | | 3.60E-01 | | 9.70E-01 | | -2.286 (7.168) | | | | 7.53E-01 | | 7.83E-01 | | 0 | | 0.559 (0.691) | | | 6.47E-01 | | | 7.82E-01 | |  |
| *Note:* DBP: diastolic blood pressure; SBP: systolic blood pressure; BMI: body mass index; Moderate: number of days/week of moderate physical activity 10+ minutes; Vigorous: Number of days/week of vigorous physical activity 10+ minutes; Heavy DIY: e.g., weeding, lawn mowing, carpentry, digging; Light DIY: e.g., pruning, watering the lawn; Walking: walking for pleasure (not as a means of transport); Bold font indicates that the MR results are significant after FDR correction (q < 0.05). | | | | | | | | | | | | | | | | | | | | | | | | | | | | | | | | | | | |  |

# Table S18. Test for heterogeneity and pleiotropy in the causal inference of the CVH factors on HannumEAA in EUR

|  | Pleiotropy test | | Heterogeneity test | |
| --- | --- | --- | --- | --- |
|  | MR-Egger intercept | p-value | Cochran’s Q statistic | p-value |
| Clinical Factor | | | | |
| TC (1SD) | 0.002 | 0.765 | 151.490 | **0.015** |
| FG (1SD) | -0.020 | **0.019** | 100.579 | 0.105 |
| DBP (1mmHg) | 0.004 | 0.284 | 1015.977 | **1.07E-07** |
| SBP (1mmHg) | 0.004 | 0.261 | 960.581 | **4.41E-07** |
| Lifestyle Factor | | | | |
| BMI (1SD) | -0.004 | 0.254 | 1017.274 | **0.040** |
| Smoking status | | | | |
| Current | 0.039 | 0.317 | 13.000 | 0.602 |
| Previous | -0.021 | 0.658 | 22.084 | 0.280 |
| Never | -0.011 | 0.564 | 82.292 | 0.264 |
| Amount of smoking | | | | |
| Packyears (1SD) | 0.015 | 0.625 | 13.710 | 0.187 |
| Number of days/week of physical activity 10+ minutes | | | | |
| Moderate (1SD) | 0.068 | 0.254 | 18.009 | 0.262 |
| Vigorous (1SD) | 0.017 | 0.840 | 4.016 | 0.947 |
| Types of physical activity in last 4 weeks | | | | |
| Heavy DIY | 0.037 | 0.480 | 13.529 | 0.759 |
| Light DIY | -0.037 | 0.473 | 15.800 | 0.149 |
| Strenuous sports | 0.008 | 0.954 | 14.915 | **0.011** |
| Walking | 0.035 | 0.642 | 13.075 | 0.874 |
| Other PA | 0.115 | 0.127 | 11.303 | 0.585 |
| No PA | 0.295 | 0.562 | 26.409 | **2.62E-05** |
| Food intake | | | | |
| Dried fruit (1SD) | -0.007 | 0.786 | 56.475 | 0.067 |
| Fresh fruit (1SD) | -0.001 | 0.937 | 50.600 | 0.643 |
| Salad (1SD) | 0.009 | 0.892 | 33.915 | **0.006** |
| Cooked vegetable (1SD) | -0.027 | 0.792 | 20.542 | 0.197 |
| Oily fish (1SD) | 0.009 | 0.638 | 69.343 | 0.432 |
| Non oily fish (1SD) | 0.026 | 0.655 | 14.261 | 0.219 |
| Cereal (1SD) | -0.001 | 0.960 | 37.549 | 0.490 |
| Bacon (1SD) | 0.022 | 0.769 | 0.192 | 0.908 |
| Processed meat (1SD) | -0.009 | 0.816 | 21.159 | 0.511 |
| *Note:* TC: total cholesterol; FG: fasting glucose; DBP: diastolic blood pressure; SBP: systolic blood pressure; BMI: body mass index; Moderate: number of days/week of moderate physical activity 10+ minutes; Vigorous: Number of days/week of vigorous physical activity 10+ minutes; Heavy DIY: e.g., weeding, lawn mowing, carpentry, digging; Light DIY: e.g., pruning, watering the lawn; Walking: walking for pleasure (not as a means of transport); Other PA: other physical activity, e.g., swimming, cycling, keep fit, bowling; No PA: none of the above physical activity; Bold font indicates the possible presence of heterogeneity or pleiotropy. | | | | |

# Table S19. Test for heterogeneity and pleiotropy in the causal inference of the CVH factors on IEAA in EUR

|  | Pleiotropy test | | Heterogeneity test | |
| --- | --- | --- | --- | --- |
|  | MR-Egger intercept | p-value | Cochran’s Q statistic | p-value |
| Clinical Factor | | | | |
| TC (1SD) | -0.004 | 0.515 | 102.077 | 0.818 |
| FG (1SD) | -0.019 | **0.019** | 86.303 | 0.410 |
| DBP (1mmHg) | 0.006 | 0.145 | 1065.003 | **2.55E-10** |
| SBP (1mmHg) | 0.001 | 0.825 | 1024.396 | **1.89E-10** |
| Lifestyle Factor | | | | |
| BMI (1SD) | 0.000 | 0.960 | 1058.960 | **0.004** |
| Smoking status | | | | |
| Current | 0.024 | 0.591 | 18.794 | 0.223 |
| Previous | -0.031 | 0.506 | 21.135 | 0.329 |
| Never | -0.005 | 0.813 | 83.757 | 0.229 |
| Amount of smoking | | | | |
| Packyears (1SD) | 0.004 | 0.921 | 18.798 | **0.043** |
| Number of days/week of physical activity 10+ minutes | | | | |
| Moderate (1SD) | -0.038 | 0.589 | 22.323 | 0.100 |
| Vigorous (1SD) | -0.085 | 0.450 | 16.150 | 0.095 |
| Types of physical activity in last 4 weeks | | | | |
| Heavy DIY | -0.016 | 0.787 | 22.321 | 0.218 |
| Light DIY | 0.028 | 0.514 | 10.369 | 0.584 |
| Strenuous sports | -0.022 | 0.819 | 6.585 | 0.253 |
| Walking | 0.102 | 0.203 | 19.001 | 0.522 |
| Other PA | 0.007 | 0.923 | 11.184 | 0.595 |
| No PA | 0.228 | 0.269 | 4.320 | 0.364 |
| Mineral and other dietary supplements | | | | |
| Dried fruit (1SD) | 0.037 | 0.135 | 43.594 | 0.403 |
| Fresh fruit (1SD) | -0.019 | 0.303 | 66.545 | 0.137 |
| Salad (1SD) | -0.002 | 0.962 | 16.451 | 0.434 |
| Cooked vegetable (1SD) | 0.178 | 0.060 | 88.324 | 0.422 |
| Oily fish (1SD) | 0.041 | 0.053 | 15.696 | **0.049** |
| Non oily fish (1SD) | -0.003 | 0.966 | 40.257 | 0.153 |
| Cereal (1SD) | 0.020 | 0.436 | 0.714 | 0.371 |
| Bacon (1SD) | -0.050 | 0.558 | 31.342 | 0.700 |
| Processed meat (1SD) | -0.076 | 0.090 | 8.981 | 0.089 |
| *Note:* TC: total cholesterol; FG: fasting glucose; DBP: diastolic blood pressure; SBP: systolic blood pressure; BMI: body mass index; Moderate: number of days/week of moderate physical activity 10+ minutes; Vigorous: Number of days/week of vigorous physical activity 10+ minutes; Heavy DIY: e.g., weeding, lawn mowing, carpentry, digging; Light DIY: e.g., pruning, watering the lawn; Walking: walking for pleasure (not as a means of transport); Other PA: other physical activity, e.g., swimming, cycling, keep fit, bowling; No PA: none of the above physical activity; Bold font indicates the possible presence of heterogeneity or pleiotropy. | | | | |

# Table S20. Test for heterogeneity and pleiotropy in the causal inference of the CVH factors on PhenoEAA in EUR

|  | Pleiotropy test | | Heterogeneity test | |
| --- | --- | --- | --- | --- |
|  | MR-Egger intercept | p-value | Cochran’s Q statistic | p-value |
| Clinical Factor | | | | |
| TC (1SD) | -0.002 | 0.818 | 147.736 | **0.025** |
| FG (1SD) | -0.031 | **0.002** | 82.846 | 0.515 |
| DBP (1mmHg) | -0.001 | 0.875 | 872.917 | **0.024** |
| SBP (1mmHg) | -0.003 | 0.502 | 938.414 | **5.39E-06** |
| Lifestyle Factor | | | | |
| BMI (1SD) | 0.000 | 0.969 | 1070.162 | **0.002** |
| Smoking status | | | | |
| Current | 0.040 | 0.426 | 15.158 | 0.440 |
| Previous | -0.054 | 0.452 | 32.770 | **0.026** |
| Never | -0.028 | 0.334 | 122.942 | **4.04E-04** |
| Amount of smoking | | | | |
| Packyears (1SD) | 0.014 | 0.657 | 9.118 | 0.521 |
| Number of days/week of physical activity 10+ minutes | | | | |
| Moderate (1SD) | -0.006 | 0.926 | 7.312 | 0.948 |
| Vigorous (1SD) | 0.013 | 0.938 | 20.597 | **0.024** |
| Types of physical activity in last 4 weeks | | | | |
| Heavy DIY | -0.085 | 0.210 | 17.942 | 0.459 |
| Light DIY | -0.041 | 0.473 | 12.798 | 0.384 |
| Strenuous sports | -0.266 | 0.115 | 18.139 | **0.003** |
| Walking | -0.095 | 0.338 | 18.894 | 0.529 |
| Other PA | 0.063 | 0.539 | 15.192 | 0.296 |
| No PA | 0.223 | 0.383 | 4.404 | 0.354 |
| Mineral and other dietary supplements | | | | |
| Dried fruit (1SD) | 0.009 | 0.779 | 51.142 | 0.158 |
| Fresh fruit (1SD) | -0.009 | 0.678 | 50.786 | 0.636 |
| Salad (1SD) | 0.027 | 0.658 | 16.087 | 0.447 |
| Cooked vegetable (1SD) | 0.064 | 0.649 | 24.672 | 0.076 |
| Oily fish (1SD) | 0.021 | 0.404 | 78.992 | 0.170 |
| Non oily fish (1SD) | 0.074 | 0.260 | 7.235 | 0.780 |
| Cereal (1SD) | 0.022 | 0.488 | 24.733 | 0.952 |
| Bacon (1SD) | -0.080 | 0.681 | 4.623 | 0.099 |
| Processed meat (1SD) | -0.008 | 0.879 | 26.892 | 0.215 |
| *Note:* TC: total cholesterol; FG: fasting glucose; DBP: diastolic blood pressure; SBP: systolic blood pressure; BMI: body mass index; Moderate: number of days/week of moderate physical activity 10+ minutes; Vigorous: Number of days/week of vigorous physical activity 10+ minutes; Heavy DIY: e.g., weeding, lawn mowing, carpentry, digging; Light DIY: e.g., pruning, watering the lawn; Walking: walking for pleasure (not as a means of transport); Other PA: other physical activity, e.g., swimming, cycling, keep fit, bowling; No PA: none of the above physical activity; Bold font indicates the possible presence of heterogeneity or pleiotropy. | | | | |

# Table S21. Test for heterogeneity and pleiotropy in the causal inference of the CVH factors on GrimEAA in EUR

|  | Pleiotropy test | | Heterogeneity test | |
| --- | --- | --- | --- | --- |
|  | MR-Egger intercept | p-value | Cochran’s Q statistic | p-value |
| Clinical Factor | | | | |
| TC (1SD) | -0.007 | 0.347 | 196.015 | **4.96E-06** |
| FG (1SD) | -0.013 | 0.165 | 124.245 | **0.003** |
| DBP (1mmHg) | 0.002 | 0.579 | 918.263 | **0.001** |
| SBP (1mmHg) | 0.005 | 0.150 | 860.464 | **0.004** |
| Lifestyle Factor | | | | |
| BMI (1SD) | -0.005 | 0.183 | 1036.000 | **0.015** |
| Smoking status | | | | |
| Current | -0.017 | 0.664 | 12.140 | 0.668 |
| Previous | -0.131 | **0.028** | 38.896 | **0.005** |
| Never | 0.033 | 0.094 | 91.801 | 0.091 |
| Amount of smoking | | | | |
| Packyears (1SD) | -0.035 | 0.311 | 17.166 | 0.071 |
| Number of days/week of physical activity 10+ minutes | | | | |
| Moderate (1SD) | 0.037 | 0.546 | 17.193 | 0.307 |
| Vigorous (1SD) | 0.074 | 0.497 | 15.189 | 0.125 |
| Types of physical activity in last 4 weeks | | | | |
| Heavy DIY | 0.011 | 0.838 | 14.007 | 0.729 |
| Light DIY | -0.107 | 0.080 | 26.896 | **0.008** |
| Strenuous sports | -0.028 | 0.822 | 11.404 | **0.044** |
| Walking | -0.037 | 0.637 | 19.408 | 0.495 |
| Other PA | -0.001 | 0.989 | 15.738 | 0.264 |
| No PA | 0.225 | 0.300 | 5.327 | 0.255 |
| Mineral and other dietary supplements | | | | |
| Dried fruit (1SD) | 0.020 | 0.473 | 58.237 | **0.049** |
| Fresh fruit (1SD) | -0.019 | 0.420 | 80.505 | **0.014** |
| Salad (1SD) | -0.047 | 0.477 | 31.079 | **0.013** |
| Cooked vegetable (1SD) | 0.008 | 0.949 | 30.351 | **0.016** |
| Oily fish (1SD) | -0.001 | 0.971 | 77.300 | 0.206 |
| Non oily fish (1SD) | 0.062 | 0.235 | 10.123 | 0.519 |
| Cereal (1SD) | 0.033 | 0.190 | 34.750 | 0.621 |
| Bacon (1SD) | -0.016 | 0.833 | 0.635 | 0.728 |
| Processed meat (1SD) | 0.020 | 0.616 | 24.278 | 0.333 |
| *Note:* TC: total cholesterol; FG: fasting glucose; DBP: diastolic blood pressure; SBP: systolic blood pressure; BMI: body mass index; Moderate: number of days/week of moderate physical activity 10+ minutes; Vigorous: Number of days/week of vigorous physical activity 10+ minutes; Heavy DIY: e.g., weeding, lawn mowing, carpentry, digging; Light DIY: e.g., pruning, watering the lawn; Walking: walking for pleasure (not as a means of transport); Other PA: other physical activity, e.g., swimming, cycling, keep fit, bowling; No PA: none of the above physical activity; Bold font indicates the possible presence of heterogeneity or pleiotropy. | | | | |

# Table S22. Test for heterogeneity and pleiotropy in the causal inference of the CVH factors on DNAmPAI-1 in EUR

|  | Pleiotropy test | | Heterogeneity test | |
| --- | --- | --- | --- | --- |
|  | MR-Egger intercept | p-value | Cochran’s Q statistic | p-value |
| Clinical Factor | | | | |
| TC (1SD) | -0.014 | 0.296 | 52.436 | 0.942 |
| FG (1SD) | -0.041 | **0.040** | 74.663 | 0.705 |
| DBP (1mmHg) | 0.007 | 0.394 | 773.885 | 0.652 |
| SBP (1mmHg) | 0.006 | 0.486 | 765.188 | 0.361 |
| Lifestyle Factor | | | | |
| BMI (1SD) | -0.006 | 0.509 | 966.846 | 0.236 |
| Smoking status | | | | |
| Current | -0.125 | 0.188 | 14.944 | 0.455 |
| Previous | -0.175 | 0.102 | 20.151 | 0.386 |
| Never | -0.046 | 0.353 | 89.515 | 0.106 |
| Amount of smoking | | | | |
| Packyears (1SD) | 0.017 | 0.787 | 8.447 | 0.585 |
| Number of days/week of physical activity 10+ minutes | | | | |
| Moderate (1SD) | 0.039 | 0.769 | 11.639 | 0.706 |
| Vigorous (1SD) | 0.155 | 0.447 | 7.239 | 0.703 |
| Types of physical activity in last 4 weeks | | | | |
| Heavy DIY | -0.169 | 0.199 | 10.237 | 0.924 |
| Light DIY | 0.086 | 0.613 | 26.416 | **0.009** |
| Strenuous sports | -0.126 | 0.532 | 2.234 | 0.816 |
| Walking | 0.086 | 0.679 | 23.172 | 0.280 |
| Other PA | -0.111 | 0.524 | 5.849 | 0.951 |
| No PA | -0.202 | 0.649 | 1.847 | 0.764 |
| Mineral and other dietary supplements | | | | |
| Dried fruit (1SD) | 0.021 | 0.729 | 46.232 | 0.302 |
| Fresh fruit (1SD) | -0.011 | 0.781 | 54.111 | 0.509 |
| Salad (1SD) | 0.128 | 0.352 | 14.520 | 0.560 |
| Cooked vegetable (1SD) | -0.249 | 0.255 | 12.700 | 0.695 |
| Oily fish (1SD) | -0.059 | 0.212 | 69.216 | 0.436 |
| Non oily fish (1SD) | -0.014 | 0.907 | 6.175 | 0.861 |
| Cereal (1SD) | 0.158 | 0.011 | 44.307 | 0.223 |
| Bacon (1SD) | 54.070 | 0.268 | 5.259 | 0.072 |
| Processed meat (1SD) | 0.042 | 0.691 | 21.348 | 0.499 |
| *Note:* TC: total cholesterol; FG: fasting glucose; DBP: diastolic blood pressure; SBP: systolic blood pressure; BMI: body mass index; Moderate: number of days/week of moderate physical activity 10+ minutes; Vigorous: Number of days/week of vigorous physical activity 10+ minutes; Heavy DIY: e.g., weeding, lawn mowing, carpentry, digging; Light DIY: e.g., pruning, watering the lawn; Walking: walking for pleasure (not as a means of transport); Other PA: other physical activity, e.g., swimming, cycling, keep fit, bowling; No PA: none of the above physical activity; Bold font indicates the possible presence of heterogeneity or pleiotropy. | | | | |


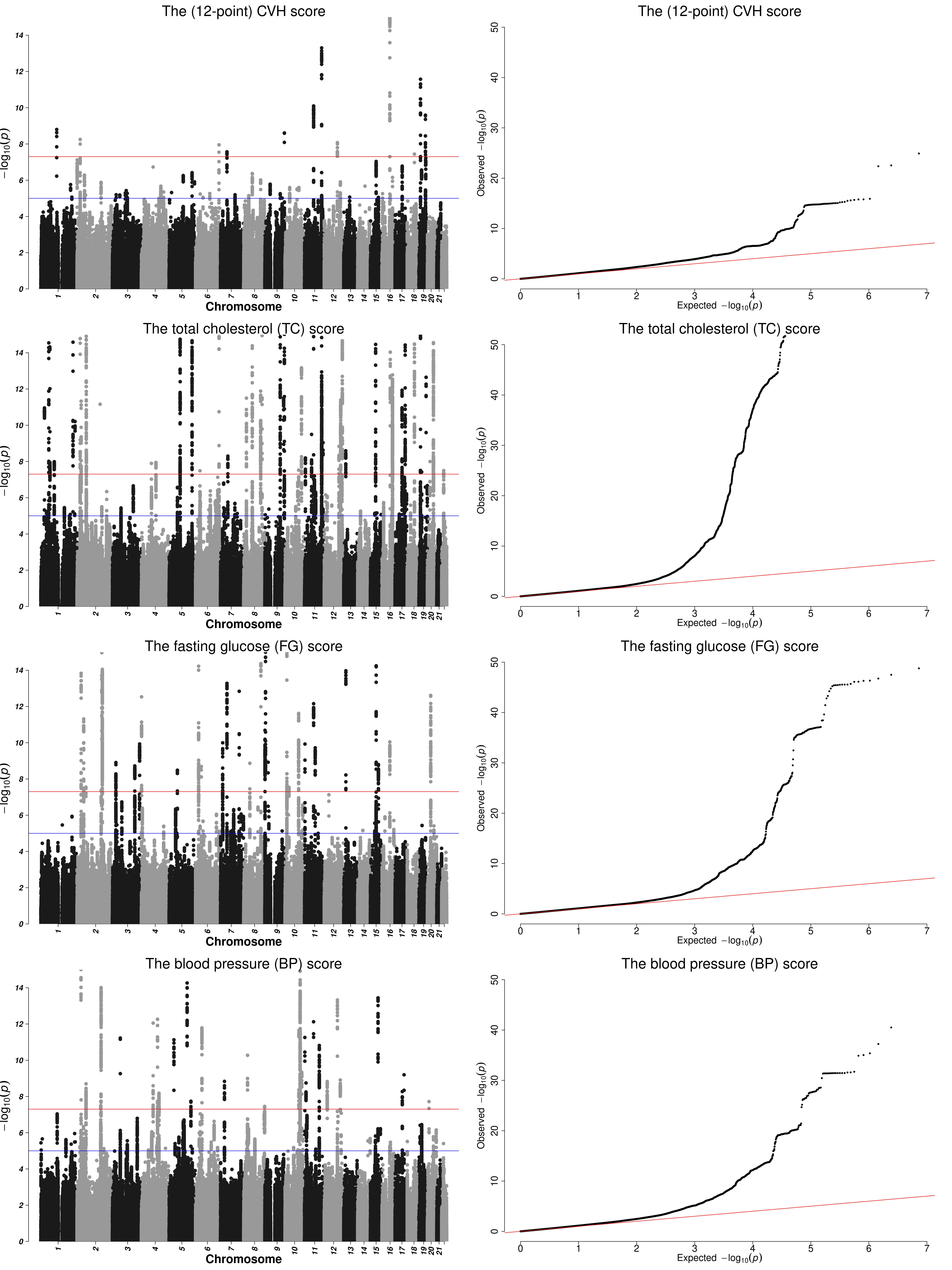


# Figure S1. The Manhattan plots and the quantile-quantile (Q-Q) plots of the (12-point) CVH score, the total cholesterol (TC) score, the fasting glucose (FG) score, and the blood pressure (BP) score

In the Manhattan plots (left column), the horizontal red and blue lines mark the genome-wide significance level (5E-8) and the suggestive significance level (1E-5). The red lines in the Q-Q plots (right column) depict that the observed p-values match the expected p-values.


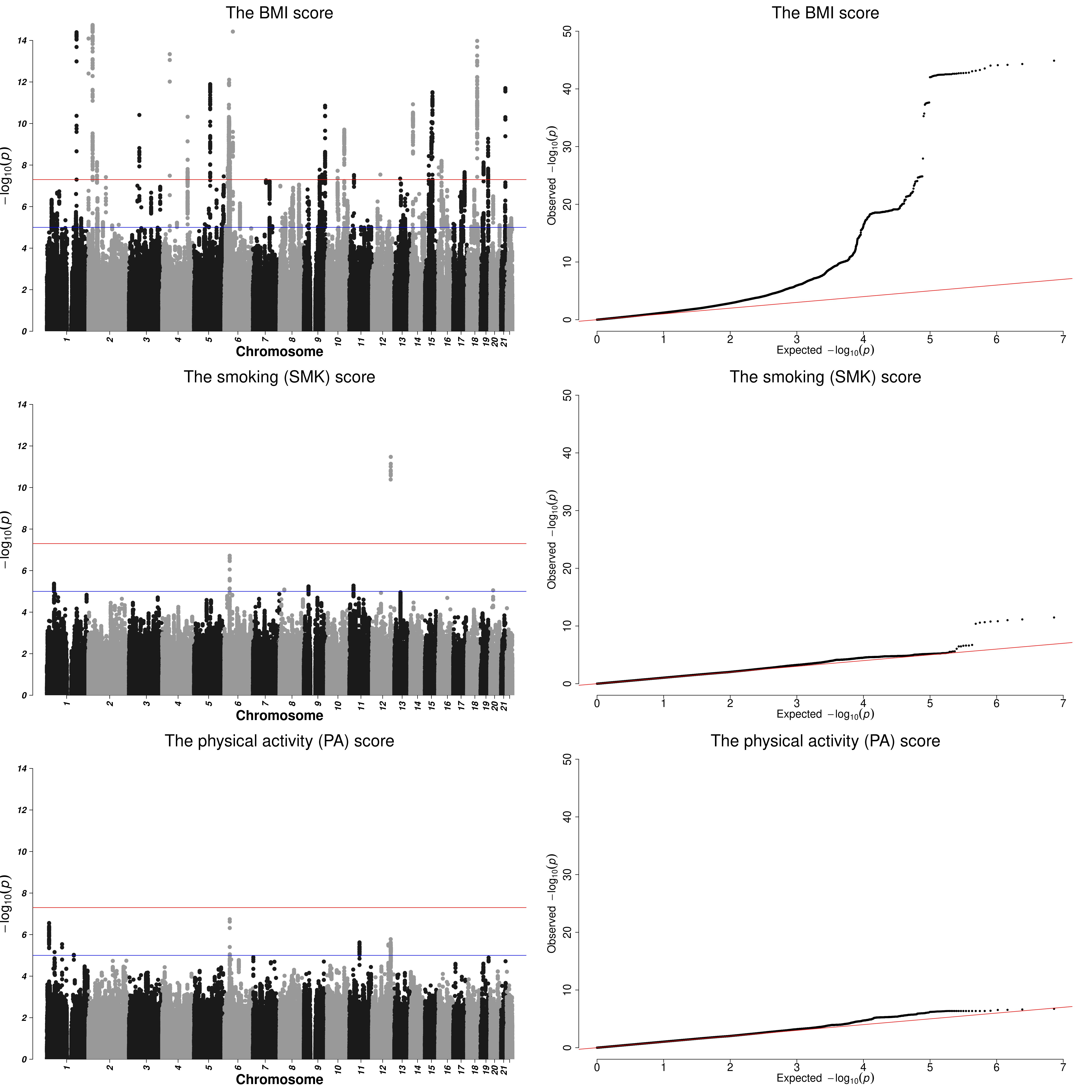


# Figure S2. The Manhattan plots and the quantile-quantile (Q-Q) plots of the BMI score, the smoking (SMK) score, and the physical activity (PA) score

In the Manhattan plots (left column), the horizontal red and blue lines mark the genome-wide significance level (5E-8) and the suggestive significance level (1E-5). The red lines in the Q-Q plots (right column) depict that the observed p-values match the expected p-values.


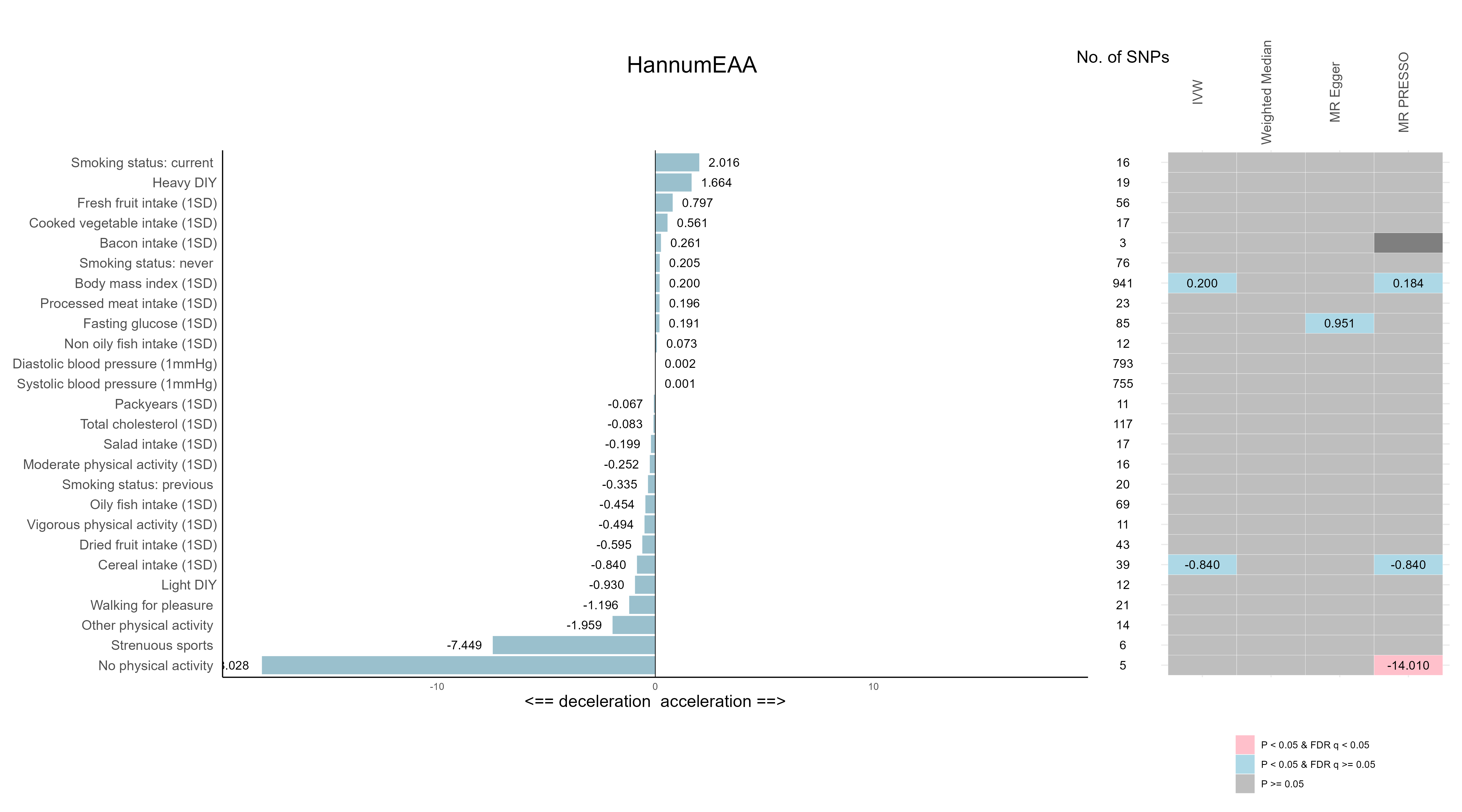


# Figure S3. The causal effects of CVH factors on HannumEAA in EUR.

For the plot in the left panel, the numbers shown around the blue bars are the causal effect sizes. The number of SNPs between the two-panel plots represents the number of IVs. For the plot in the right panel, red boxes indicate significant causal effects (p-value < 0.05 & FDR q-value < 0.05). Blue boxes indicate suggestive causal effects (p-value < 0.05 & FDR q-value ≥ 0.05). Gray boxes indicate insignificant causal effects (p-value ≥ 0.05). The dark gray box indicates insufficient IVs for the MR analysis. The box numbers represent the causal effect sizes achieving significant or suggestive associations.


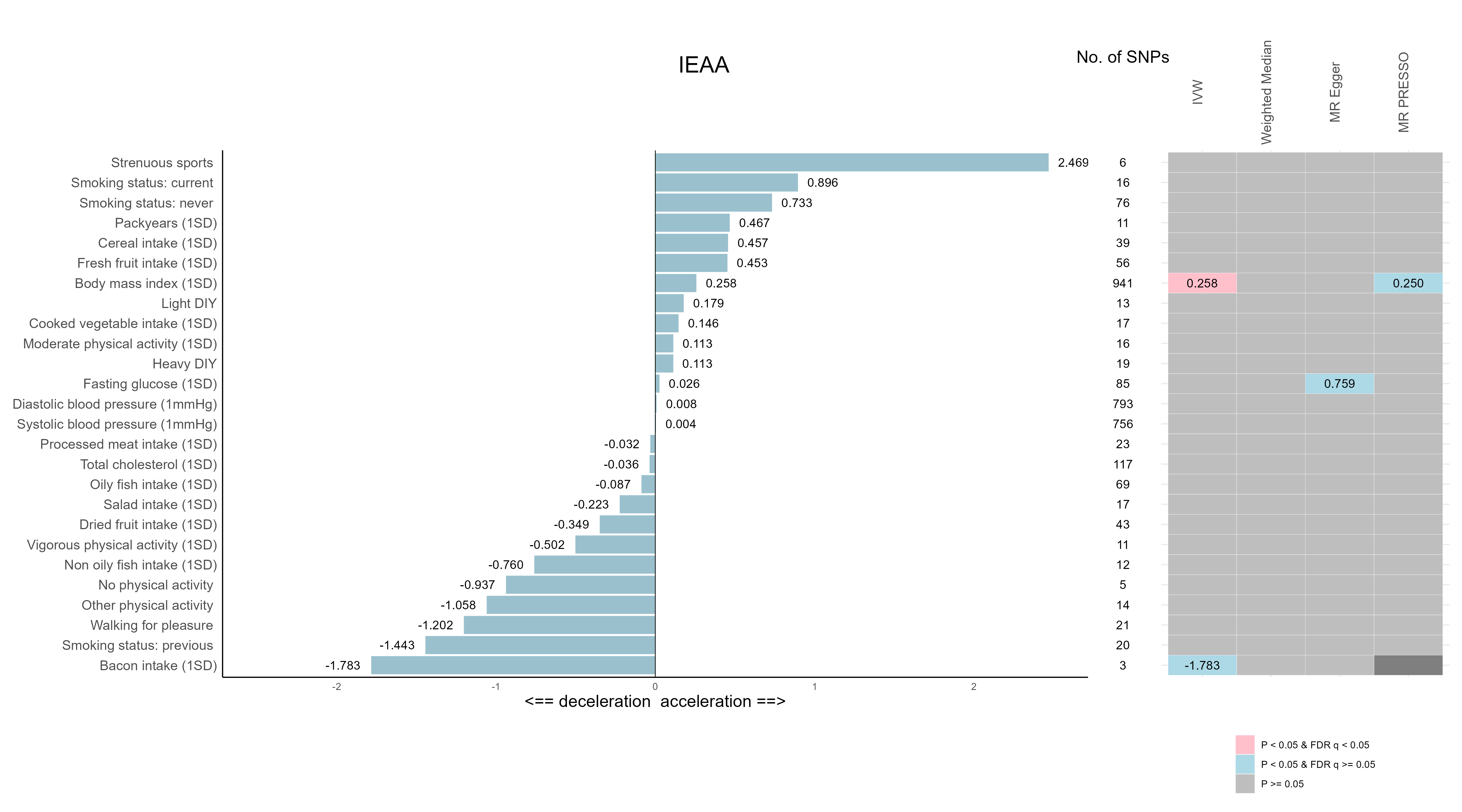


# Figure S4. The causal effects of CVH factors on IEAA in EUR.

For the plot in the left panel, the numbers shown around the blue bars are the causal effect sizes. The number of SNPs between the two-panel plots represents the number of IVs. For the plot in the right panel, red boxes indicate significant causal effects (p-value < 0.05 & FDR q-value < 0.05). Blue boxes indicate suggestive causal effects (p-value < 0.05 & FDR q-value ≥ 0.05). Gray boxes indicate insignificant causal effects (p-value ≥ 0.05). The dark gray box indicates insufficient IVs for the MR analysis. The box numbers represent the causal effect sizes achieving significant or suggestive associations.


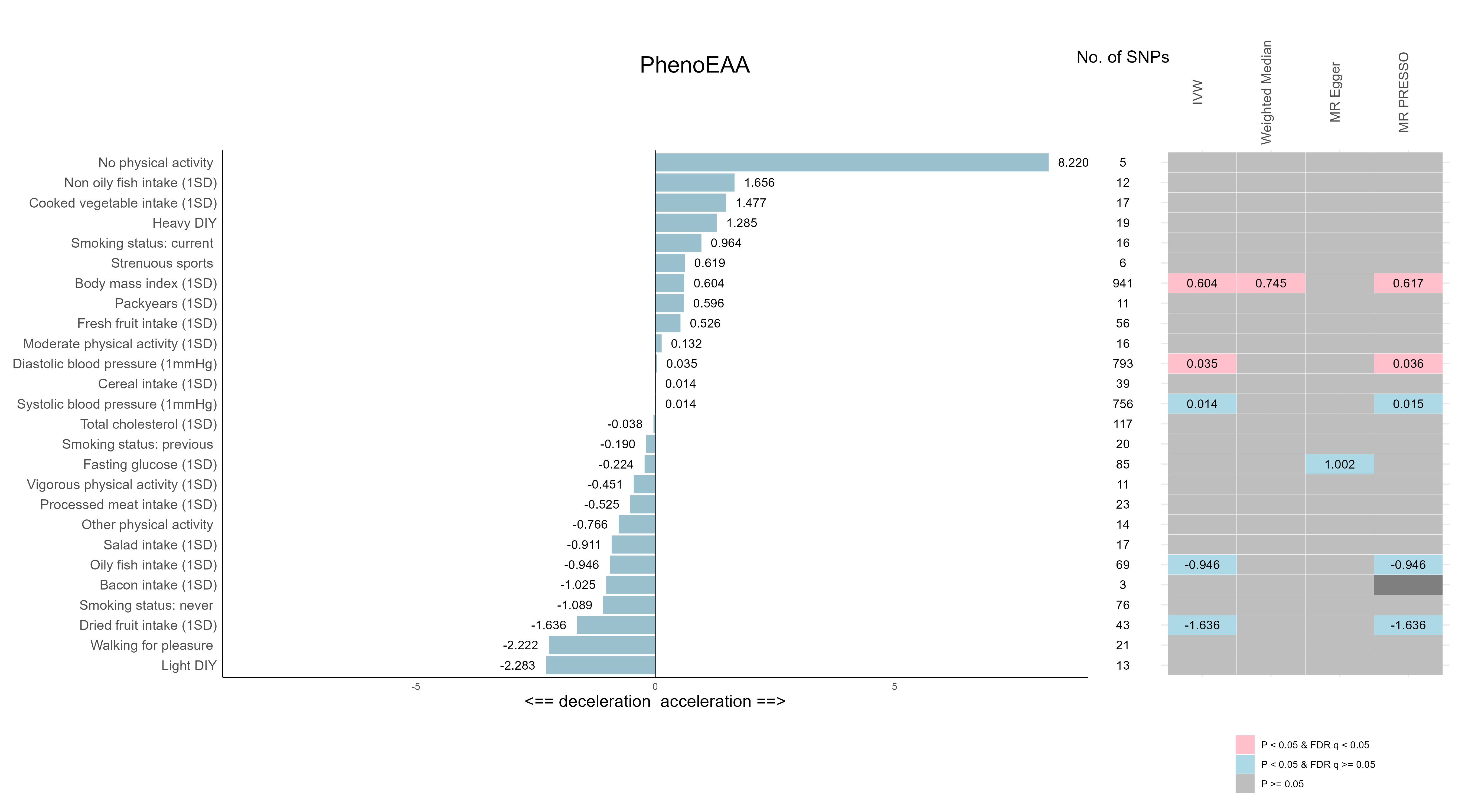


# Figure S5. The causal effects of CVH factors on PhenoEAA in EUR.

For the plot in the left panel, the numbers shown around the blue bars are the causal effect sizes. The number of SNPs between the two-panel plots represents the number of IVs. For the plot in the right panel, red boxes indicate significant causal effects (p-value < 0.05 & FDR q-value < 0.05). Blue boxes indicate suggestive causal effects (p-value < 0.05 & FDR q-value ≥ 0.05). Gray boxes indicate insignificant causal effects (p-value ≥ 0.05). The dark gray box indicates insufficient IVs for the MR analysis. The box numbers represent the causal effect sizes achieving significant or suggestive associations.


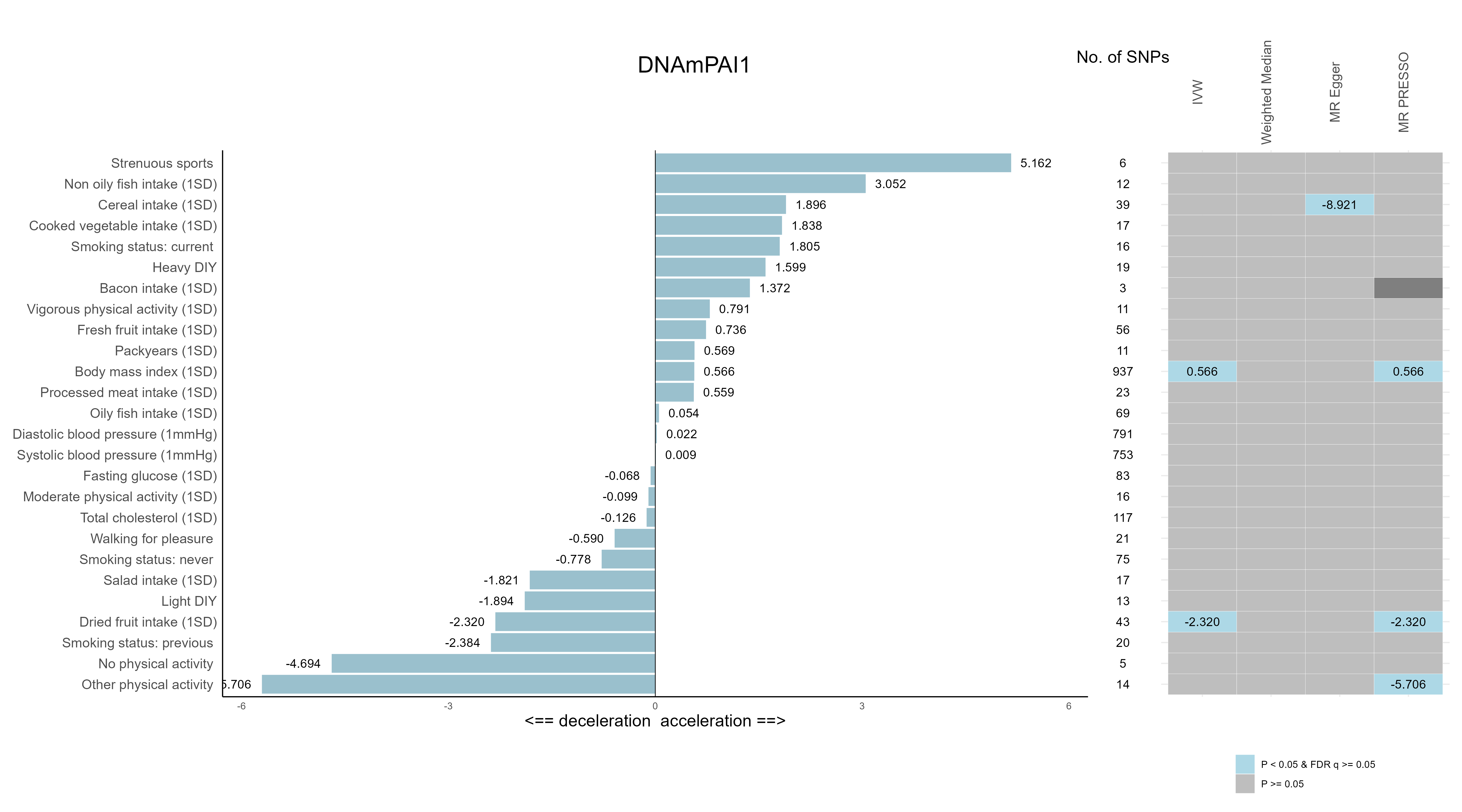


# Figure S6. The causal effects of CVH factors on DNAmPAI-1 in EUR.

For the plot in the left panel, the numbers shown around the blue bars are the causal effect sizes. The number of SNPs between the two-panel plots represents the number of IVs. For the plot in the right panel, red boxes indicate significant causal effects (p-value < 0.05 & FDR q-value < 0.05). Blue boxes indicate suggestive causal effects (p-value < 0.05 & FDR q-value ≥ 0.05). Gray boxes indicate insignificant causal effects (p-value ≥ 0.05). The dark gray box indicates insufficient IVs for the MR analysis. The box numbers represent the causal effect sizes achieving significant or suggestive associations.

# Reference

1. Evangelou E, Warren HR, Mosen-Ansorena D, Mifsud B, Pazoki R, Gao H, Ntritsos G, Dimou N, Cabrera CP, Karaman I *et al*: **Genetic analysis of over 1 million people identifies 535 new loci associated with blood pressure traits**. *Nat Genet* 2018, **50**(10):1412-1425.

2. Willer CJ, Schmidt EM, Sengupta S, Peloso GM, Gustafsson S, Kanoni S, Ganna A, Chen J, Buchkovich ML, Mora S *et al*: **Discovery and refinement of loci associated with lipid levels**. *Nat Genet* 2013, **45**(11):1274-1283.

3. Chen J, Spracklen CN, Marenne G, Varshney A, Corbin LJ, Luan J, Willems SM, Wu Y, Zhang X, Horikoshi M *et al*: **The trans-ancestral genomic architecture of glycemic traits**. *Nat Genet* 2021, **53**(6):840-860.

4. Yengo L, Sidorenko J, Kemper KE, Zheng Z, Wood AR, Weedon MN, Frayling TM, Hirschhorn J, Yang J, Visscher PM *et al*: **Meta-analysis of genome-wide association studies for height and body mass index in approximately 700000 individuals of European ancestry**. *Hum Mol Genet* 2018, **27**(20):3641-3649.

5. Elsworth B, Mitchell R, Raistrick C, Paternoster L, Hemani G, Gaunt T: **MRC IEU UK Biobank GWAS pipeline version 2**. In*.*: University of Bristol; 2019.
